# How Communities of Marine Stramenopiles Varied with Environmental and Biological Variables in the Subtropical Northwestern Pacific Ocean

**DOI:** 10.1007/s00248-021-01788-7

**Published:** 2021-07-16

**Authors:** Yun-Chi Lin, Chien-Pang Chin, Jinny Wu Yang, Kuo-Ping Chiang, Chih-hao Hsieh, Gwo-Ching Gong, Chi-Yu Shih, Szu-Ying Chen

**Affiliations:** 1 Institute of Marine Environment and Ecology, National Taiwan Ocean University, Keelung, Taiwan; 2 Fishery Research Institute, Keelung, Taiwan; 3 Ecology and Evolutionary Biology, University of Michigan, Ann Arbor, USA; 4 Institute of Oceanography, National Taiwan University, Taipei, Taiwan; 5 Center of Excellence for the Oceans, National Taiwan Ocean University, Keelung, Taiwan; 6 Institute of Marine Biology, National Taiwan Ocean University, Keelung, Taiwan

**Keywords:** 18S rDNA V4 amplicon, Ecological distribution, Heterotrophic protists

## Abstract

MArine STramenopiles (MASTs) have been recognized as parts of heterotrophic protists and contribute substantially to protist abundances in the ocean. However, little is known about their spatiotemporal variations with respect to environmental and biological factors. The objectives of this study are to use canonical correspondence analysis to investigate how MASTs communities are shaped by environmental variables, and co-occurrence networks to examine their potential interactions with prokaryotic communities. Our dataset came from the southern East China Sea (sECS) in the subtropical northwestern Pacific, and involved 14 cruises along a coastal-oceanic transect, each of which sampled surface water from 4 to 7 stations. MASTs communities were revealed by metabarcoding of 18S rDNA V4 region. Most notably, MAST-9 had a high representation in warm waters in terms of read number and diversity. Subclades of MAST-9C and -9D showed slightly different niches, with MAST-9D dominating in more coastal waters where concentrations of nitrite and *Synechococcus* were higher. MAST-1C was a common component of colder water during spring. Overall, canonical correspondence analysis showed that MASTs communities were significantly influenced by temperature, nitrite and *Synechococcus* concentrations. The co-occurrence networks showed that certain other minor prokaryotic taxa can influence MAST communities. This study provides insight into how MASTs communities varied with environmental and biological variables.

**Supplementary Information:**

The online version contains supplementary material available at 10.1007/s00248-021-01788-7.

## Introduction

Heterotrophic protists are ubiquitous in the oceans, with an abundance ranging from 10^2^ to 10^4^ cells ml^−1^ [1]. Their great metabolic versatility enables them to perform multiple functions in marine ecosystems [2, 3]. They act as consumers of prokaryotic and eukaryotic picoplankton [4, 5], and some can parasitize and symbiose with different microbes [6, 7]. They are a crucial trophic link in microbial food webs, and influence the taxonomic composition and physiological status of microbial communities [8]. This group influences recycling of carbon and nutrients in marine ecosystems. However, unlike photosynthetic protists, heterotrophic protists have received relatively little attention, due mainly to their lack of cultures and remarkable morphological features [2]. So far, little information is available about their ecological distribution within higher taxonomic levels in marine ecosystems.

Heterotrophic protists comprise bicosoecids, chanoflagellates, cercozoa, dinoflagellates, diplomonads, MArine STramenopiles (MASTs), Katablepharidophyta, Telonemia, and some heterotrophic chrysophytes. MASTs are essentially unknown, but represent a substantial part of the diversity and abundance among heterotrophic protists [9–11]. Studying their ecologic distribution would help to elucidate their functional roles. Previous studies have used 18S rRNA gene sequencing to establish 18 MAST clades (MAST-1 to -4, -6 to -12, -16, -20 to -25), numbered according to when they were discovered [9, 11]. Sequence data have shown that MAST-1, -3, -4, and -7 are the most common in ocean surface water [9, 12]. QPCR and FISH methods have been used to quantify MAST-1, -4 and -7, suggesting that they contribute significantly to abundances of heterotrophic protists [12–14]. MAST-1 (mostly -1C), -4, and -7 contribute a relatively stable percentage in marine ecosystems, and account for 10–20% of heterotrophic protists [12, 14–16]. In terms of abundance, the aforementioned MASTs contribute a total of around one hundred cells per milliliter at the surface of the world’s oceans [12, 14–16]. Due to their extremely diverse 18S rDNA, few studies have been able to quantify MAST-3 abundance. Moreover, there has been little research on the ecological distribution of MASTs communities with respect to environmental variables. To the best of our knowledge, only Massana et al. [11] have studied the distribution of MASTs in the world’s oceans, but subtropical-tropical regions have been poorly investigated. Our previous studies indicated that MASTs represent a substantial proportion of heterotrophic protist abundance in subtropical-tropical regions, and maximum cell numbers of MAST-4 and MAST-1C reached approximately 2000 and 500 cells ml^−1^, respectively, in the East China Sea [15]. Additionally, MASTs have been reported to play multiple roles in microbial food webs, for example as active grazers on bacteria and algae [4, 15, 17], and as symbionts with diatoms and cyanobacteria [7]. Co-occurrence relationships may exist between MASTs and prokaryotic communities, but so far few studies have discussed these interactions.

Our study hypothesizes a co-occurrence relationship between MASTs and prokaryotic communities, e.g. prey-predator, parasite-host, substrate provider and shared habitat. We studied how the community composition of MASTs varied with environmental factors, and also performed co-occurrence network analysis with prokaryotes under different hydrographic conditions. This study took place over four years, in a coastal-offshore transect. Sampling in such heterogeneous environments could provide useful information about how MASTs communities vary with the environment. The objective of this study is to provide insight into the composition of MASTs in the subtropical Pacific Ocean, and describe their relationships with environmental and biological variables.

## Materials and methods

### Sample collection and processing

Sampling was conducted along a coastal-oceanic transect on the marginal seas of the western Pacific Ocean in the southern East China Sea (sECS) from April, 2014 to September, 2017 (Fig. S1). Samples were taken at 4–7 stations on each of 14 cruises, resulting in a total of 90 samples. The sampling transect extended from the Min river plume to the oceanic water of the Kuroshio current (Fig. S1). Water samples were collected using Go-Flo bottles mounted on a CTD rosette (conductivity, temperature, and depth) (Sea-Bird Electronics, USA) at the surface layer. For DNA samples, ~ 20 l of surface water (3 m depth) was pre-filtered through a 20 µm nylon net, then subsequently filtered through 1.2-µm and 0.2-µm pore size polycarbonate membranes (142 mm diameter, Millipore, USA) with a peristaltic pump. The filters were immediately preserved in liquid nitrogen and stored at − 80 °C until DNA extraction. Picoplankton cell numbers (heterotrophic bacteria, *Prochlorococcus*, *Synechococcus* and photosynthetic picoeukaryotes) were counted by flow cytometry. An aliquot of 2 ml seawater was preserved with paraformaldehyde (0.2% final concentration), then frozen in liquid nitrogen and stored at − 80 °C until processed. Pigmented picoplankton were discriminated without staining, by orange and red fluorescence, while heterotrophic bacteria were discriminated by red and green fluorescence after staining with SYBR Green 1/10,000 [19]. Temperature and salinity were measured using a CTD profiler. NO_2_, NO_3_, PO_4_ and SiO_3_ were measured according to standard methods used in previous studies [18].

### PCR amplification of 18S rDNA V4 region and 16S rDNA V5-V6 region

Total DNA for 18S and 16S was extracted from size-fractionated samples of 1.2–20 µm and 0.2–1.2 µm, respectively, using a PowerWater DNA isolation kit (Qiagen), in accordance with the manufacturer’s guidelines. PCR for 18S rDNA V4 region were amplified using eukaryotic universal primers set TAReuk454FWD1 (5’-[illumina adaptor]- CCAGCASCYGCGGTAATTCC-3’) and TAReukREV3 (5’- [illumina adaptor]- ACTTTCGTTCTTGATYRA-3’) [20]. The PCR conditions for 18S were an initial denaturation at 95 °C for 3 min; 30 cycles at 94 °C for 30 s, 47 °C for 45 s, 72 °C for 30 s, and a final extension at 72 °C for 2 min. PCR primers for 16S rDNA V5-V6 region used prokaryotic universal primers set 787F (5’-[illumina adaptor]- ATTAGATACCCNGGTAG-3’) and 1046R (5’-[illumina adaptor]- CGACAGCCATGCANCACCT-3’) [21]. The PCR conditions for 16S were an initial denaturation at 94 °C for 3 min; 25 cycles at 94 °C for 30 s, 55 °C for 45 s, 72 °C for 1 min, and a final extension at 72 °C for 2 min. PCR mixtures contained GoTaq DNA polymerase (Promega), 2 ng of template DNA and a final concentration of 200 µM dNTPs, 2.5 mM MgCl_2_ and 0.2 µM primers. PCR products were purified with Agencourt AMPure XP PCR purification (Beckman Coulter) and followed by an additional PCR for ligating unique dual-indexes (with S5 and N7 indexes as primers, Nextera Index Kit) for each sample. This additional PCR condition was an initial denaturation at 94 °C for 3 min; 6 cycles at 94 °C for 30 s, 55 °C for 45 s, 72 °C for 1 min; and a final extension at 72 °C for 2 min. Purified DNA with unique dual-indexes were pooled with approximately the same concentration and sent for amplicon sequencing, carried out on an Illumina MiSeq platform (2 × 300 bp paired-end run).

### Quality control of amplicon dataset

The analysis of the reads of 18S rDNA V4 region and 16 rDNA V5-V6 region was done by standard pipeline DADA2 package version 1.12.1 [22] under R 3.6.1. DADA 2 uses exact amplicon sequence variants (ASVs) instead of operational taxonomic units (OTUs) (supplementary files 1 and 2 for 18S and 16S, respectively). Prior to DADA2 pipeline, we trimmed the primers with Cutadapt [23]. The 18S amplicon raw data of OSD2014 was downloaded from the website (http://mb3is.megx.net/osd-files?path=/2014/datasets/raw). A total of 155 LGC samples were analyzed (library preparation and sequencing were performed at LGC genomics, formerly the Laboratory of the Government Chemist). The taxonomy assignments for 16S and 18S were done with the SILVA 132 database and PR2 version 4.12.0 database, respectively. To study the phylogenetic relationships of MAST-9, the 10 most abundant ASVs from the OSD and sECS data were used to analyze the phylogeny of 18S rDNA V4 region. The raw sequences have been deposited in the sequence read archive of GenBank under accession numbers PRJNA662424 for 16S and 18S.

### Analysis of MASTs communities, prokaryotic communities and environmental parameters

MAST amplicons were screened using the search keyword, “MAST” from an original 18S ASV table. Samples with total MAST reads < 200 were excluded from further analysis. Subsequently, 63 out of 90 MAST samples were used, and randomly subsampled to 209 for 100 times, using a rarefy function in vegan [24]. To simplify the taxonomic composition, ASVs were collapsed using subclade level (“order” taxonomic ranks in PR2) (supplementary file 3). If the ASVs at the subclade level were unassigned, we merged them based on clade level (“class” taxonomic ranks in PR2). This generated 49 MAST taxa. For 16S amplicon, we subsampled the minimum reads of 37,548, then collapsed the ASVs using “class” level in SILVA (supplementary file 4).

Canonical correspondence analysis (CCA) was used to determine the environmental factors influencing MASTs communities, including temperature, salinity, nitrite (NO_2_), nitrate (NO_3_), phosphate (PO_4_), silicate (SiO_3_), abundances of bacteria, *Synechococcus* and photosynthetic picoeukaryotes. The significance of their contribution was tested with ANOVA using the Vegan package. Automatic forward selection with significance tests of Monte Carlo permutations were used to build optimal models. Additionally, we did a PERMANOVA test to check the importance of various environmental factors in affecting community composition. To explore how the dynamics of MAST abundances varied with environmental variables, we performed a generalized additive model (GAM) to determine the most suitable habitats of the 10 most abundant MAST taxa using a mgvc package. Taxa abundances were natural log transformed prior to GAM modeling. The co-occurrence between the communities of MASTs and prokaryotes was analyzed with an sPLS regression model implemented in the mixOmics R package [25]. The subsampled data were used to analyze the spatial distribution of MASTs, CCA, clustering by MAST composition, co-occurrence networks, and GAM modeling. All statistical analyses were done with R 3.6.1.

## Results

### Hydrographic conditions

A coastal-offshore transect was studied where Station (St) 1 was closest to the coast, and St 12 was farthest away (Fig. S1). The Min River inputs (characterized by a salinity of < 31) may bring nutrients into the coastal region, which is also influenced by the cold China Coastal Current that flows southward along the coast during spring (Fig. S1). The Taiwan Warm Current flows from south of the Taiwan Strait into the continental shelf of the sECS (Sts 3 and 5). It is characterized by medium-salinity and high-temperature. The oligotrophic Kuroshio flows northward across the offshore area, causing upwelling events at Sts 9 and 11 as the deeper water of the mid-layer Kuroshio brings cooler and saltier water to the surface [26]. During the study period, salinity varied from 28.1 to 34.6, with a median of 33.86 at the surface while temperature ranged from 19.5 (April, 2014) to 30.0 °C (September, 2014) (supplementary file 5). The study region was a relatively warm water system compared to other global studies.

### Amplicon information

Following DADA2 pipeline, the sECS dataset consisted of 7,199,876 and 6,967,240 quality reads of 16S and 18S, respectively. For 16S amplicon, the median read number was 74,425 with a minimum and maximum of 15,896 and 188,456, respectively. For 18S amplicon, the median read number was 78,467, with a minimum and maximum of 10,011 to 165,335. A total of 162,769 reads were assigned to MASTs, comprising 434 ASVs (supplementary file 6). Overall, MASTs amplicons contributed an average of 2.0% of total reads in all samples. After removing samples with low MAST reads (< 200 reads in a sample), a subset of 157,197 reads from 63 samples were used for further analysis. This dataset involved comprehensive sampling carried out between spring and autumn, and from coast to offshore (Fig. S2). Data in winter were not available, due to rough weather conditions.

### The spatial distribution of the common MASTs in the sECS

**Fig. 1:**
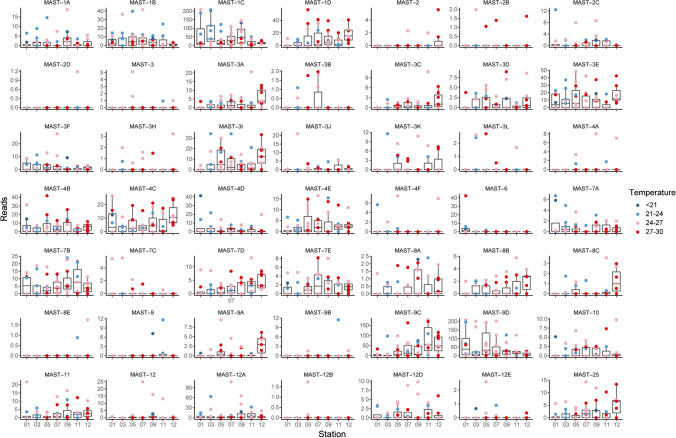
Spatial variation of MAST reads (49 taxa) at stations, with each dot representing one sample, and the colors blue, sky blue, pink and red, corresponding to temperature levels of < 21 °C, 21–24 °C, 24–27 °C, and 27–30 °C, respectively. Boxplots show the median values as horizontal lines, and interquartile ranges as boxes with whiskers extending to 1.5 times the interquartile range. Note that 209 MASTs reads were subsampled for 100 times in the samples

MAST-1, -3, -4, and -7 have been known as the common MASTs in the oceans [9, 12], and they contributed > 1% of total MAST reads after subsampling in the sECS (Fig. S3). MAST-1 to -4, -6 to -12, and -25 were detected in the surface sECS. Notably, MAST-9D contributed the most abundant reads here, followed by MAST-1C (Fig. S3). Among MAST-1 taxa, MAST-1C contributed the highest reads and the greatest diversity, with 22 ASVs out of a total of 51 ASVs (supplementary file 6). In the sECS, MAST-1C appears to be more abundant in continental-shelf waters, upwelling waters and occasionally in coastal water where the temperature ranged from 21 to 27 °C (Fig. 1 and supplementary file 7). MAST-1D was the second most abundant taxon (Fig. S3), contributing 17 ASVs and inhabiting warm offshore waters (Fig. 1). MAST-1B abundance peaked around 25 °C (supplementary file 7), while MAST-1A occasionally peaked when seawater was cooler (< 24 °C) (Fig. 1). MAST-3 were the most diverse group, with 135 ASVs in this study (supplementary file 6). *Solenicola setigera* and *Incisomonas marina* have been identified as belonging to MAST-3I and 3 J, respectively, and *Solenicola setigera* was recognized as the diatom symbiont [7, 27]. In the sECS, MAST-3E contributed the maximum reads in MAST-3 (Fig. S3), whereas MAST-3I provided higher diversity (32 ASVs vs. 19 ASVs for MAST-3E, supplementary file 6). These two taxa had low abundances in more eutrophic coastal water (St 1) and upwelling water (Sts 9 and 11) (Fig. 1). Our observations show that MAST-3 is relatively less abundant in coastal waters, and appears to prefer mesotrophic to oligotrophic waters (Fig. 1). In this study, MAST-4 comprised 26 ASVs (supplementary file 6), with MAST-4C contributing the most abundant reads. MAST-4 subclades contributed similar numbers, except for MAST-4A and -4F, which were particularly low in this study (Fig. S3). GAM modeling showed that MAST-4C increased with temperature (supplementary file 6). Overall, the distribution pattern of MAST-4 displayed a broad salinity tolerance, thriving from brackish to oceanic water (Fig. S4). In the sECS, MAST-7 comprised 45 ASVs (supplementary file 6), and was dominated by MAST-7B on read numbers (Fig. S3). MAST-7B had low reads at Sts 3 and 5 in continental shelf water that was frequently influenced by the Taiwan Warm Current (Fig. 1). Overall, MAST-7 showed a preference for warm offshore waters, except for the minor taxon MAST-7A, which occasionally peaked in cold coastal waters (Fig. 1).

**Fig. 2:**
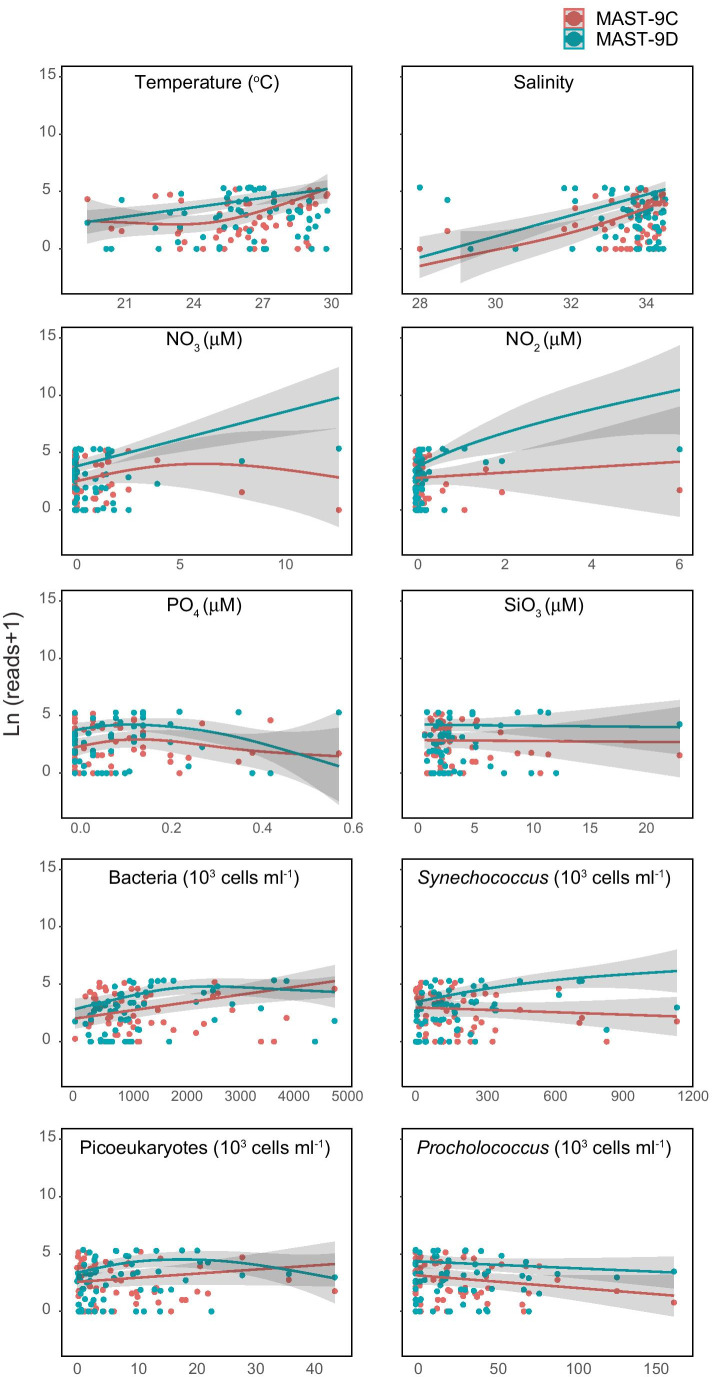
Prediction curves (colored lines) and 95% confidence intervals (gray shading) of the distribution of MAST-9C (pink) and 9D (green) based on generalized additive models (GAM) with respect to temperature (^o^C), salinity, nutrients (NO_3_, NO_2_, PO_4_ and SiO_3_ [µM]), picoplankton abundance (heterotrophic bacteria, *Synechococcus*, *Prochlorococcus*, and photosynthetic picoeukaryotes [10^3^ cells ml^−1^]). Note that MAST reads were transferred by Ln (reads + 1)

MAST-9C and -9D showed a preference for warm waters (Fig. 1), but each was represented in different water characteristics. We illustrated their environmental niches using GAM modeling (Fig. 2). Both MAST-9C and -9D were found to increase with temperature and salinity, and MAST-9C showed a lower abundance in temperatures between 23 and 26 °C (Fig. 2). Although there was a greater abundance of MAST-9C in offshore Kuroshio water (Sts 11 and 12) (Fig. 1), in terms of salinity, the MAST 9C and 9D niches overlapped at 95% confidence intervals (Fig. 2). With respect to other variables, MAST-9D preferred more coastal water (Fig. 1) containing higher concentrations of *Synechococcus* and NO_2_ (Fig. 2).

### The spatial distribution of minor MASTs in the sECS

Apart from MAST-12A, MAST-2, -6, -8, -10, -11, -12 and -25 were minor components of MASTs in the sECS (< 1% of total MAST reads after subsampling, Fig. S3). In the sECS, MAST-2 comprised 13 ASVs in 4 taxa (-2, -2B, -2C and -2D, supplementary file 6), and had a broad distribution. MAST-2C peaked in cold coastal waters, while MAST-2, -2B and -2D peaked in warm offshore waters (Fig. 1). No subclades were defined in MAST-6 [11] and it was sporadically abundant in fresher coastal water (Fig. S4).

MAST-8, -10, -11, and -25 tended to be found more often in warm offshore Kuroshio water (Fig. 1). MAST-10 exhibited no clear distribution pattern, peaking in both coastal waters and offshore waters (Fig. S4). MAST-12 comprises 5 subclades, and has been recovered from diverse environments, from fresh water to ocean water, plankton to sediment, and oxic to anoxic water [11]. In the sECS, MAST-12A dominated, and was generally more abundant in saline shelf water (Fig. S4). It appeared in a moderate number of samples (37/90, Fig. S3), but suddenly peaked in certain samples, for example at St 3 in May and at St 7 in June of 2017 (Fig. 1).

### How environments drive the MASTs communities

**Fig. 3:**
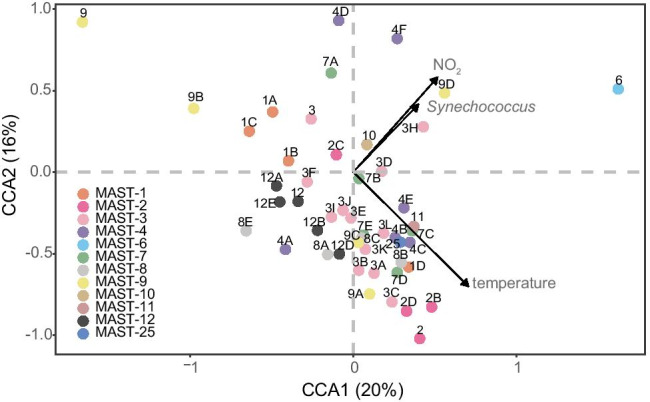
Canonical correspondence analysis (CCA) biplot illustrating the relationship between environmental variables and MASTs communities. The arrows represent significant environmental variables and each dot represents a MAST taxon. The results show that temperature, nitrite and *Synechococcus* concentrations are significant factors driving MASTs community composition

Results of canonical correspondence analysis (CCA) show how whole MASTs communities are influenced by environmental variables. In our study, temperature, nitrite and *Synechococcus* concentrations were significant variables, responsible for 36% of total variance in MAST composition (Fig. 3). Subclades are scattered far apart, indicating the diverse niches within this dynamic hydrographic system. Apart from MAST-3H, -4F, -6 and -9D, which preferred environments with high NO_2_ and high *Synechococcus* concentrations, temperature was the key driver for the majority of MASTs (Fig. 3). However, MAST-6 was not as common as MAST-9D, and occasionally appeared in coastal waters (Fig. 1). With respect to temperature, MAST-1A, -1B, -1C, -4A -4D and -7A preferred cooler waters, whereas MAST-1D, -4B, -4C, -4E, -7C, -7D, -8B, -9C and -11 preferred relatively warm waters (Fig. 3). Overall, the majority of MASTs taxa were more abundant in offshore waters, with only a few taxa peaking in fresher coastal waters (salinity < 32.5), including MAST-1C, -4D, -6, -7B, and -9D (Fig. S4).

### Interactions between MASTs communities and prokaryotes in the sECS

**Fig. 4:**
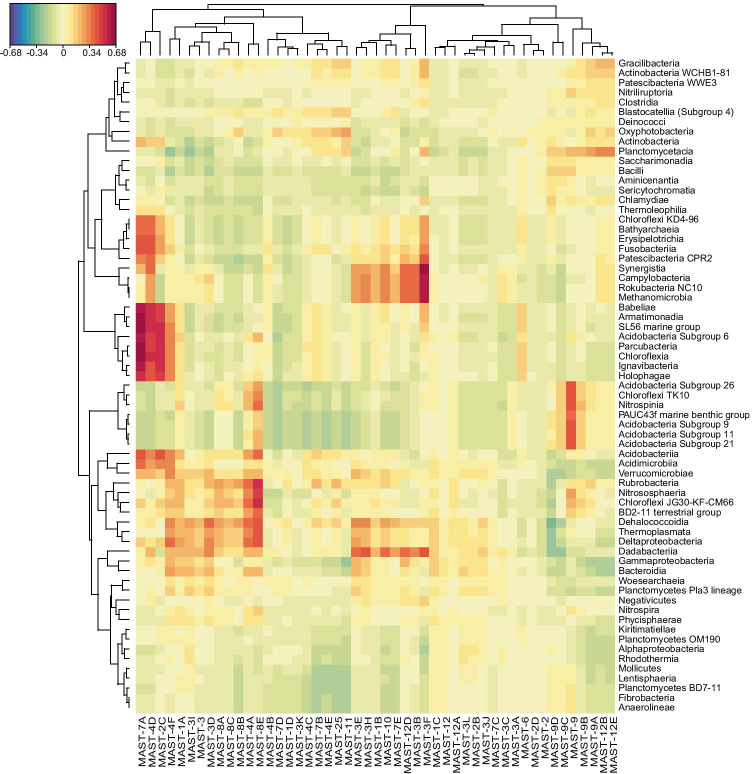
Heatmap showing co-occurrences between the taxonomic groups of MAST and prokaryotic communities based on a sPLS function using relative correlations (mixOmics). The heatmap colors indicate the correlation coefficients. Missing data for one sample for the prokaryotic community (Or2057St01) was due to PCR failure

Apart from physiochemical parameters, prokaryotes play an important role in influencing protist communities, perhaps through endosymbiosis, parasitism, substrate provision, and as prey. Notably, co-occurrence between MASTs and prokaryotes may reflect shared environmental preferences, as well as biological interactions. Prokaryotic communities were dominated by Alphaproteobacteria, Gammaproteobacteria, Planctomycetacia, Acidimicrobiia and Oxyphotobacteria, accounting for 29%, 19%, 18%, 14% and 10%, respectively, of total 16S reads after subsampling. We examined the co-occurrence between MASTs and prokaryotes communities to analyze the networks in microbial food webs. High positive correlations were found between MAST-9 and certain minor prokaryotes, such as *Chloroflexi* TK10, Acidobacteria (subgroup 9, 11, 21, and 26), and Nitrospinia nitrite-oxidizing bacteria (NOB) (Fig. 4). Both MAST-9 and prokaryotes occasionally appeared together in upwelling regions, and this correlation implies specific functional roles. As mentioned above, MAST-9D shows a preference for higher NO_2_ and *Synechococcus* concentrations, and MAST-9 may therefore play an unknown role in the nitrogen cycle. MAST-2C, -4D and -7A, which generally peaked in fresher coastal water (Fig. S4), were positively correlated with Ignavibacteria, SL56, and Acidobacteria subgroup 6 (Fig. 4), reflecting a shared habitat. These findings may be useful for future research into the interaction between MASTs and prokaryotes.

### MASTs communities in distinct hydrographic regions

**Fig. 5:**
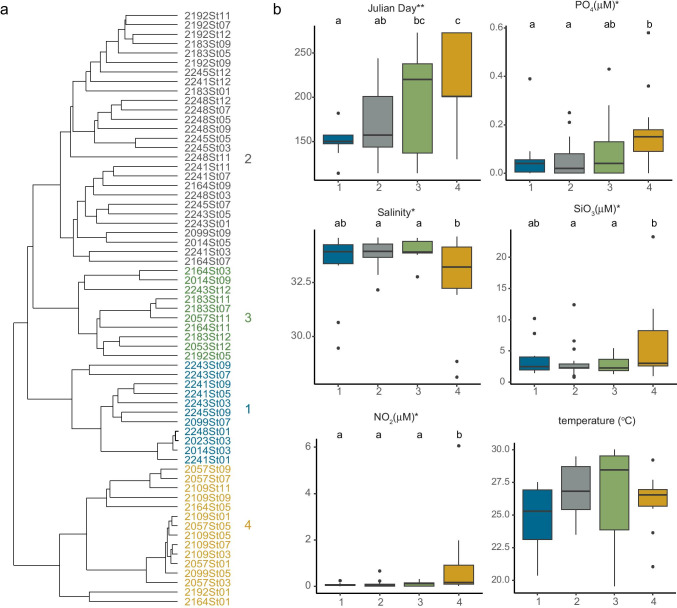
(**a**) MAST composition in four clusters based on hierarchical clustering of ASV counts. (**b**) Distribution patterns of Julian day, temperature, salinity, NO_2_, PO_4_ and SiO_3_ concentrations in these four clusters. Boxplots show the median values as horizontal lines, and interquartile ranges as boxes with whiskers extending to 1.5 times the interquartile range. Dots indicate the outliers. Significant differences between clusters shown with ANOVA (*P < 0.05; ***P < 0.001) and median sharing a different letter are significantly different according to Tukey’s post hoc test. The fraction of the MASTs taxa from the average of each cluster is shown in Fig. S5. There was no significant temperature difference between these four clusters, but it was an important factor driving MASTs communities in CCA

In this transect of the sECS, the hydrographic conditions showed a strong spatial–temporal variability [18]. To study how the MASTs community varied with environments, we clustered datasets based on taxonomic composition and then inspected the environmental variables in each cluster. Having delineated four clusters (Fig. 5), we used ANOVA to look for significant environmental variables (temperature, salinity, Julian day, NO_2_, PO_4_, SiO_3_, and *Prochlorococcus* concentrations). Cluster 1 was dominated by MAST-1C (Fig. S5) and showed a relatively high occurrence of MAST-12A. It appeared during spring in waters with lower-temperature and salinity (Fig. 5a and b) resembling the characteristics of the China Coastal Current. These samples were distributed in the western part of the sECS (Sts. 1, 3, 5, 7 and 9). Clusters 3 and 4 were dominated by MAST-9C and MAST-9D, respectively (Fig. S5). In cluster 3, 6 out of 10 samples were taken from offshore water at Sts. 11 and 12 (Fig. 5a), so MAST-9C was more significant in offshore Kuroshio water. Compared with MAST-9C, MAST-9D was more significant in coastal regions with higher concentrations of PO_4_, SiO_3_ and NO_2_ and lower salinity (Fig. 5b). Overall, MAST-9 showed a preference for warmer waters (generally > 25 °C) from summer through autumn (Fig. 5). Cluster 2 had a high diversity of MASTs, with greater numbers of MAST-1C, -1D, -3E, -3I, -4B, -4C, -7B, -10, -11, and -25, than other clusters (Fig. S5). Cluster 2 mostly occurred in water with low nutrients and high salinity from spring to early summer (Fig. 5).

## Discussion

MAST-9 was generally found to be a minor member in MASTs communities [11], but may contribute a significant portion of MAST reads in the sECS (Fig. 5). To investigate the importance of MAST-9 in marine water, we therefore inspected the MASTs community composition in the dataset of Ocean Sampling Day (OSD) 2014, and then focused on the global distribution of MAST-9. In the OSD 2014 dataset MASTs amplicons contributed ~ 2% of total reads (90,616/4,350,002), similar to the figure obtained from the sECS. Prior to subsampling, the OSD dataset comprised 493 ASVs, slightly higher than the number in the sECS (434 ASVs). We excluded OSD data with low reads of MASTs (< 100 reads in a sample), then subsampled 100 reads for 100 times, resulting in 113 samples (out of 155) for further analysis. In the OSD data, MAST-1C was the most abundant read followed by MAST-12A, whereas MAST-9 contributed a relatively low proportion (Fig. S3). The overall MASTs composition in the OSD resembled that in cluster 1, reflecting a typical MAST composition in coastal waters (Fig. S5).

**Fig. 6:**
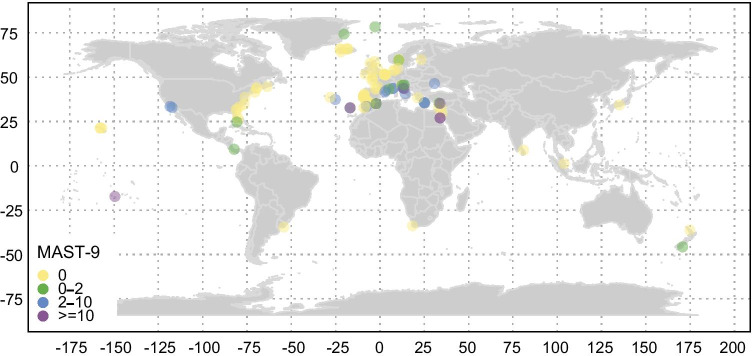
Global distribution of MAST-9 ASVs on OSD 2014. The dot colors represent the percentage levels of MAST-9 ASV reads relative to the total MASTs reads. In the OSD, a subset of 113 samples (from a total of 155 LGC samples) after subsampling 100 reads for 100 times were then used to visualize the MAST-9 percentage contribution. The global distributions of individual subclades of MAST-9 (MAST-9A, -9C and -9D) are provided in Fig. S6

**Fig. 7:**
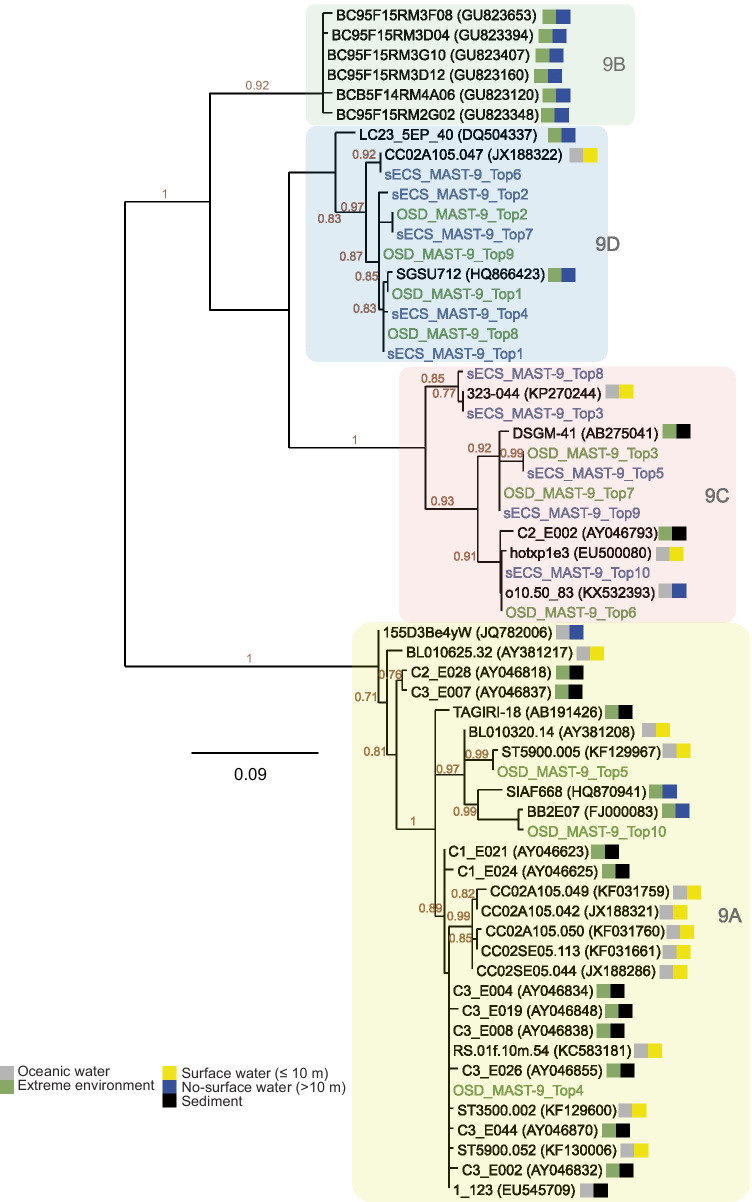
Phylogenetic relationships of MAST-9 based on 18S rDNA V4 region. The 10 most abundant MAST-9 ASVs in the sECS and OSD are indicated in blue and green, respectively. This tree was constructed using an alignment of 289 bp under PhyML. Bootstrap values are shown when > 0.7. The color in the left square indicate the types of sampling water, with gray representing oceanic water and green representing extreme environments, including anoxic water, hydrothermal water and methane cold seep; the color in the right square indicate the sampling depths with yellow representing surface water (≤ 10 m), blue representing non- surface water and black representing sediment

It is noteworthy that MAST-9 reads were detected during almost the entire sampling period in the sECS (Fig. S3). In addition, in a large number of the sECS samples (25/63), MAST communities were dominated by MAST-9C and 9D (clusters 3 and 4 in Fig. 5) with highly diverse ribotypes, composed of 52 ASVs (supplementary file 6). The size fractions collected in this study (1.2 µm -20 µm) were different from other studies on small protists (< 3 µm or < 5 µm fraction). OSD samples were not analyzed for size-fraction. In the OSD, few samples (7/113 samples) showed MAST-9 contributing > 10% of total MAST reads after subsampling. These sites were mostly located in middle and low latitude regions of the Mediterranean Sea (OSD19), Black Sea (OSD25 and OSD78), Red Sea (OSD52 and OSD53), Moorea in French Polynesia (OSD7), and the North Atlantic Ocean (OSD103) (Fig. 6). MASTs communities consisted largely of MAST-9C and -9D in the OSD 7, which is made up of a coral reef ecosystem. MAST-9 sequences have frequently been associated with extreme environments, e.g., anoxic waters [28], hydrothermal vents [29], and methane cold seep [30] (Fig. 7). In the review paper, MAST-9C was better represented in sediments, and MAST-9D in anoxic waters [11]. Our finding that MAST-9C and -9D dominated in oceanic water is therefore intriguing. In the OSD, MAST-9 consisted of 26 ASVs, just half the number of ribotypes found in the sECS. To sum up, MAST-9 had both high read numbers and great diversity in the sECS, and were the key component in MASTs communities in oxic-surface waters. Overall, MAST-9 appeared to prefer warm water (Fig. 1 and Fig. 5). Although two OSD samples in the Greenland Sea (OSD80 and OSD146) at high latitudes showed very low reads of MAST-9D (~ 2% of total MASTs, Fig. 6), so far few studies have reported the occurrence of MAST-9 in polar regions. Therefore, we hypothesize that MAST-9 is more important at low-latitudes.

Different subclades of MAST-9 showed different niche partitioning. In the OSD, read numbers of MAST-9 were dominated by MAST-9D (Fig. S3). MAST-9A was in low abundance (Fig. S3), but occur widely in many oceanic regions based on the OSD data and NCBI database (Fig. 7 and S3), e.g., the South China Sea, the Mediterranean Sea, the Red Sea, and the Northeastern Pacific Ocean (Fig. 7). Although the South China Sea is adjacent to the sECS, the dominant subclades among MAST-9 were distinct, with MAST-9A dominant in the former, and MAST-9C and -9D dominant in the latter (Fig. 7). Past studies have found MAST-9B sequences in anoxic Caribbean water [28], but they are nearly absent in the sECS and OSD data. MAST-9C dominated MASTs communities in the sECS, but appeared at only a few stations in the OSD (> 2% of MASTs reads), e.g., the Red Sea (OSD52 and 53) and Moorea (OSD7) (Fig. 6). A phylogenetic analysis of 18S rDNA V4 region revealed that the dominant MAST-9C in offshore Kuroshio water clustered with a sequence retrieved from the Sargasso Sea (323–044, KP270244) with 99% identity (350/352). The MAST-9C sequences in the OSD were identical with those in the sECS, but there was greater diversity in the latter (Fig. 7). This suggests that MAST-9C may be restricted to warm oligotrophic environments, frequently occurring alongside MAST-9D (Fig. S6). MAST-9D was comparatively common (Fig. S6), and phylogenetic analysis showed their reads from the sECS and OSD to be clustered together (Fig. 7). MAST-9D is therefore an important constituent of MASTs communities globally, something that has been overlooked in previous studies.

Temperature, salinity and O_2_ concentration have been reported as crucial factors influencing protist communities [31–33]. This study found temperature to be the most important factor overall. A thermal niche differentiation between MAST-1 subclades was observed. MAST-1D inhabit warm offshore waters, which is consistent with previous observations in Kuroshio water [34]. Also, our previous study revealed that the majority of MAST-1D sequences were retrieved from lower latitudinal regions [15]. By contrast, MAST-1A and -1C prefer to inhabit cooler waters (Fig. 3). This may be connected to the fact that the phylogenetic distribution of MAST-1 is related to latitudinal gradients, as observed in previous research [15]. Previous studies have indicated that the distribution pattern of distinct MAST-4 subclades is clearly temperature-related [3, 35], and we observed that MAST-4C reads increased with temperature based on GAM modeling (supplementary file 6). Since MAST-4C possess rhodopsins, which act as light-driven proton pumps, sufficient irradiance can support ATP generations [3]. In line with previous studies, our research found that MAST-4B, -4C and -4E prefer warm waters whereas MAST-4A and -4D prefer cooler waters (Fig. 3, supplementary file 7). A notable difference here is that a previous study showed MAST-4E to be abundant in low temperature waters at high-latitudes [3]. Our finding implies that another warm-adapted ecotype may exist within MAST-4E.

Dissolved oxygen plays an important role in shaping protist and MASTs communities [11, 32]. Apart from areas of upwelling water, dissolved oxygen was nearly saturated in surface water in the sECS [36]. Due to this limited range of values, we did not focus on exploring the influence of dissolved oxygen on MASTs distribution. We did, however, observe that MAST-9C and -9D thrived in oxic surface waters. Previous studies showed MAST-9 to be significant in anoxic waters and sediments [10, 11]. It appears that MAST-9, which comprise several species, may possess different metabolic strategies to adapt to large variations in dissolved oxygen levels. In particular, we observed that MAST-9D prefers high NO_2_ environments in coastal waters. NO_2_ can act as an alternative electron accepter where O_2_ concentration is low.

Some MASTs can be found in freshwater and ocean water, and salinity is not a factor restricting their distribution, e.g., MAST-2 and -12 [11, 37]. MAST-2 generally contribute low read numbers in many marine systems, including in this study [11], but increase in polar regions [38, 39]. Few studies have addressed the ecological distribution of MAST-12, which comprises 5 subclades and has been recovered from diverse environments, from fresh water to ocean water, plankton to sediment, and oxic to anoxic water [11]. This read was affiliated with a sequence retrieved from a volcano lake in Asia with 99% identity (386/387). In the OSD, MAST-12A had the second most abundant reads and appeared in more than half of all samples (98/155), suggesting a high contribution to MASTs communities in global coastal regions (Fig. S3). This taxon can be found from fresh to marine water. Overall, CCA ordination indicated that the distribution of MAST-12 subclades was governed by temperature (Fig. 3).

MAST-6 was sporadically abundant in coastal water (Fig. S4), and its distribution appeared to be influenced by NO_2_ and *Synechococcus* concentrations, rather than by temperature (Fig. 3). Previous studies have reported MAST-6 sequences in sediments [17], and anaerobic water [40]. Additionally, MAST-6 is an important grazer on algae and bacteria in brackish waters of the Baltic Sea, with large seasonal fluctuations in abundance [17]. In the OSD, MAST-6 contributed the third most abundant reads following MAST-1C and MAST-12A (Fig. S3), revealing that they are important constituents of protists in global coastal waters. Although some MASTs can be found in fresh water or can thrive in coastal water, the majority of MASTs are distributed in marine environments, rather than in brackish or freshwaters, which is consistent with previous studies [11].

## Conclusion

Due to their high abundance and diversity in the oceans, MASTs can serve as models of evolution and microbial biogeographic distribution. This study used spatiotemporal data to study heterotrophic protist communities against a variety of environmental and biological parameters in the subtropical Northwestern Pacific. Unlike surveys in other ocean areas, we found a dominance of MAST-9C and -9D in surface warm water, with slightly different niche partitioning. Since MAST-9 can be an important constituent of MASTs communities at low latitudes, their abundance, appearance and ecological functions should be the focus of future studies.

## Supplementary Information

Below is the link to the electronic supplementary material.

Supplementary file1 (TXT 7038 KB)

Supplementary file2 (TXT 1944 KB)

Supplementary file3 (CSV 10 KB)

Supplementary file4 (CSV 15 KB)

Supplementary file5 (XLSX 17 KB)

Supplementary file6 (XLSX 12 KB)

Supplementary file7 (PDF 1.78 MB)

Supplementary file8 (PDF 17144 KB)

## Data Availability

The code in this study is available from the first author.
