# Supplementary material for: How Communities of Marine Stramenopiles Varied with Environmental and Biological Variables in the Subtropical Northwestern Pacific Ocean
Source: Microb Ecol. 2021 Jul 16;83(4):916–28. doi: 10.1007/s00248-021-01788-7 (PMC9015972; doi:10.1007/s00248-021-01788-7)
Supplement: Supplementary file 7 — Supplementary file7 (PDF 1.78 MB) [file 248_2021_1788_MOESM7_ESM.pdf]

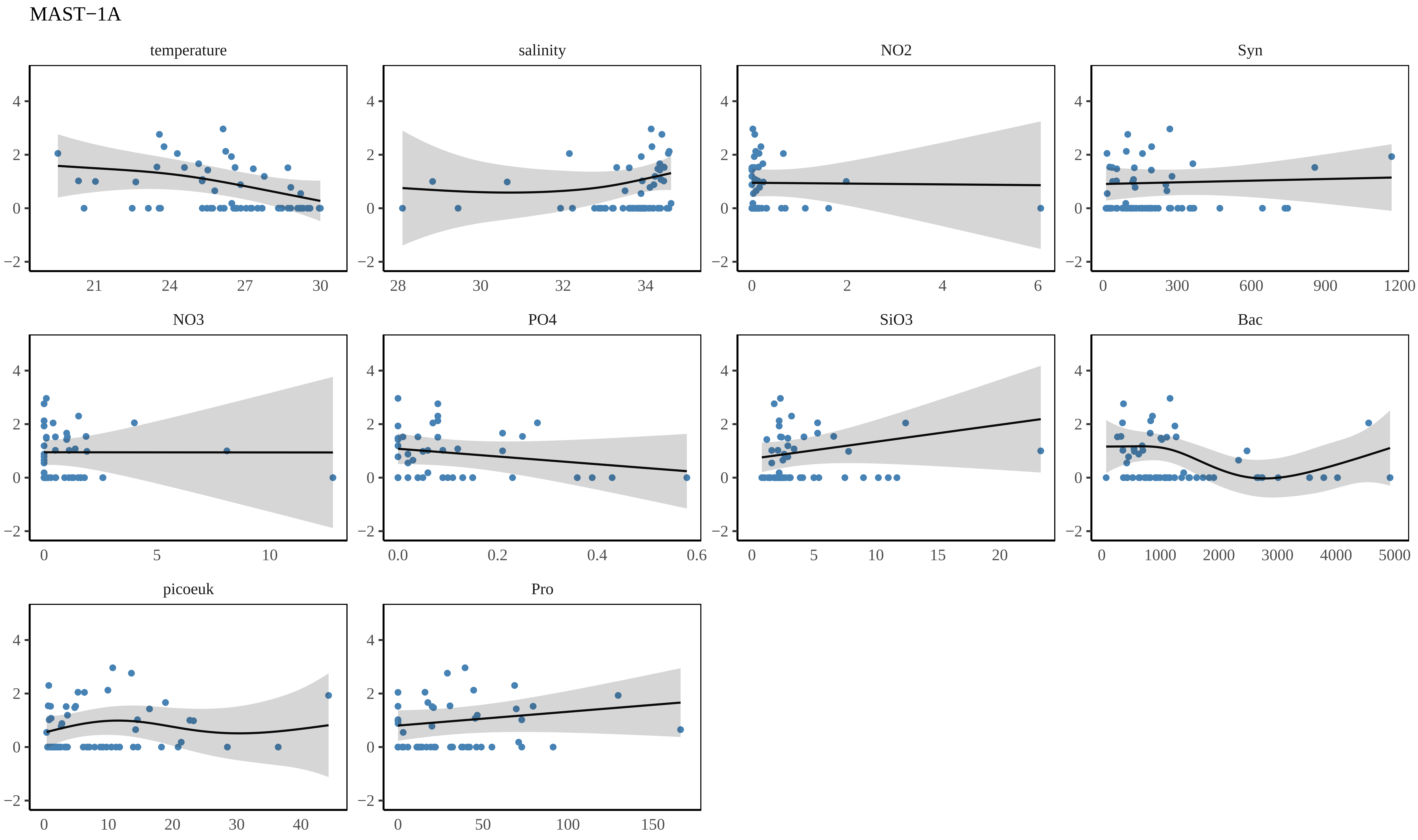

MAST−1A
temperature
salinity
NO2
Syn
NO3
PO4
SiO3
Bac
picoeuk
Pro

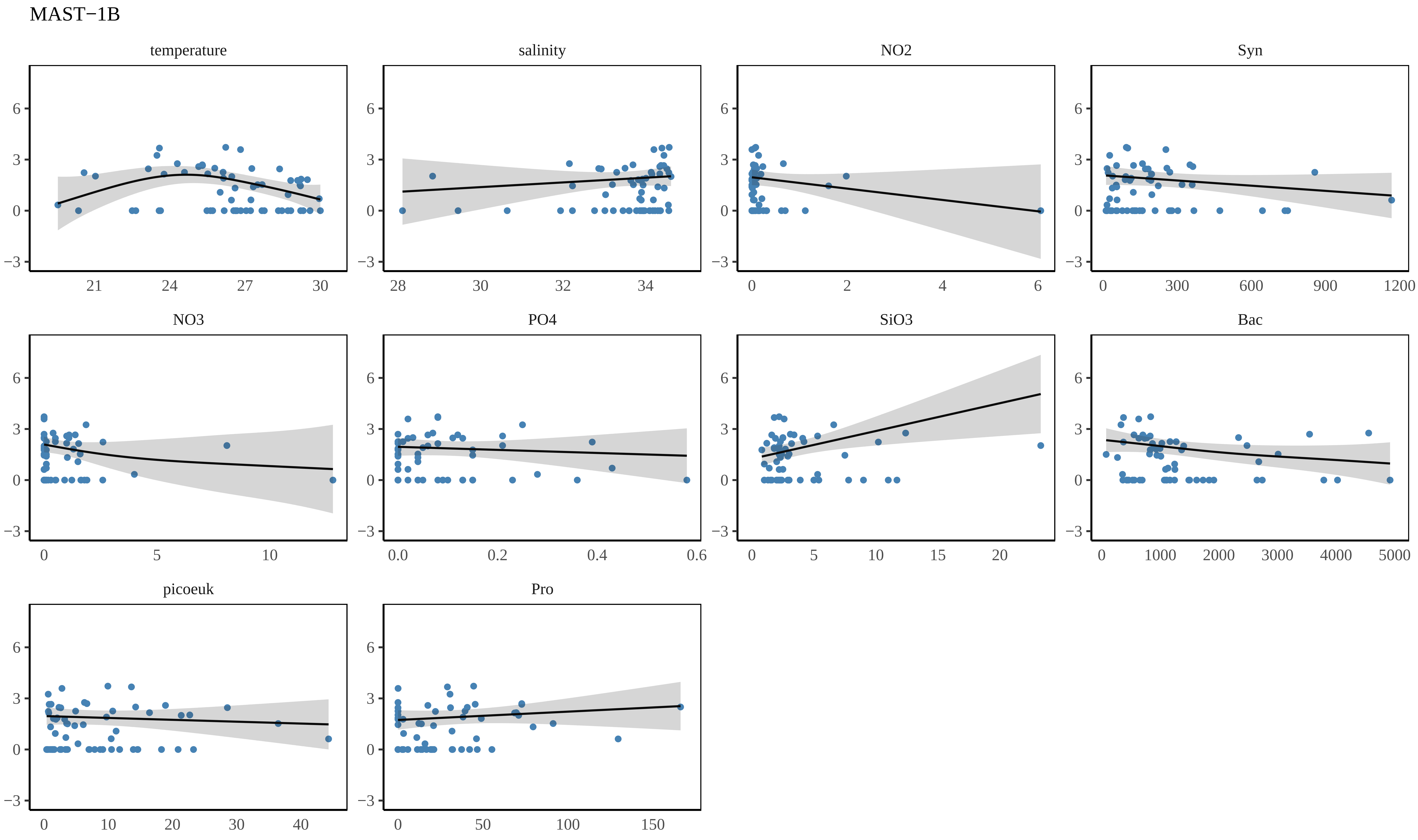
MAST−1B
temperature
salinity
NO2
Syn
NO3
PO4
SiO3
Bac
picoeuk
Pro

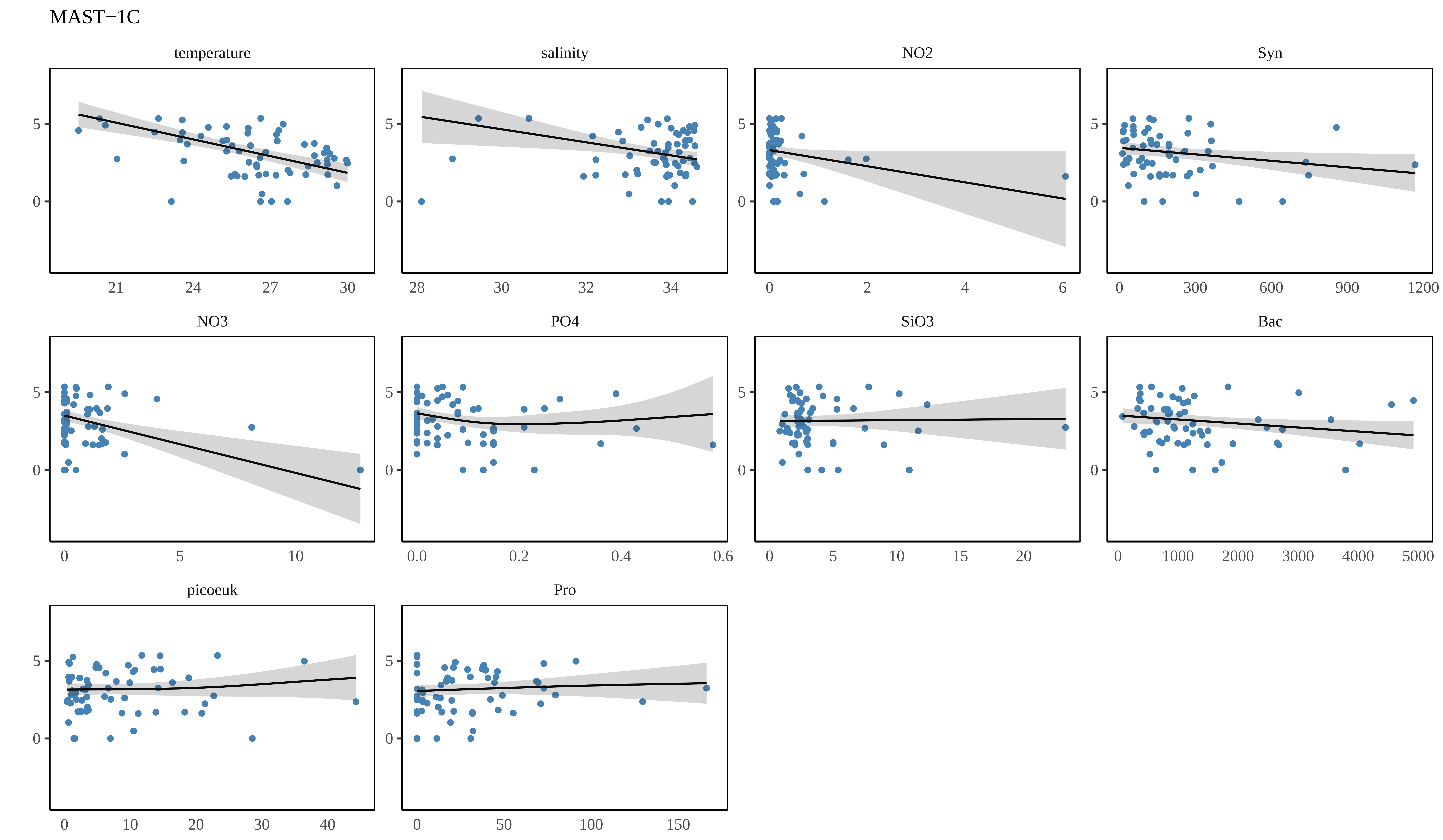
MAST−1C
temperature
salinity
NO2
Syn
NO3
PO4
SiO3
Bac
picoeuk
Pro
0
5
21
24
27
30
28
30
32
34
0
2
4
6
0
300
600
900
1200
0
5
10
0.0
0.2
0.4
0.6
0
5
10
15
20
0
1000
2000
3000
4000
5000
0
10
20
30
40
0
50
100
150

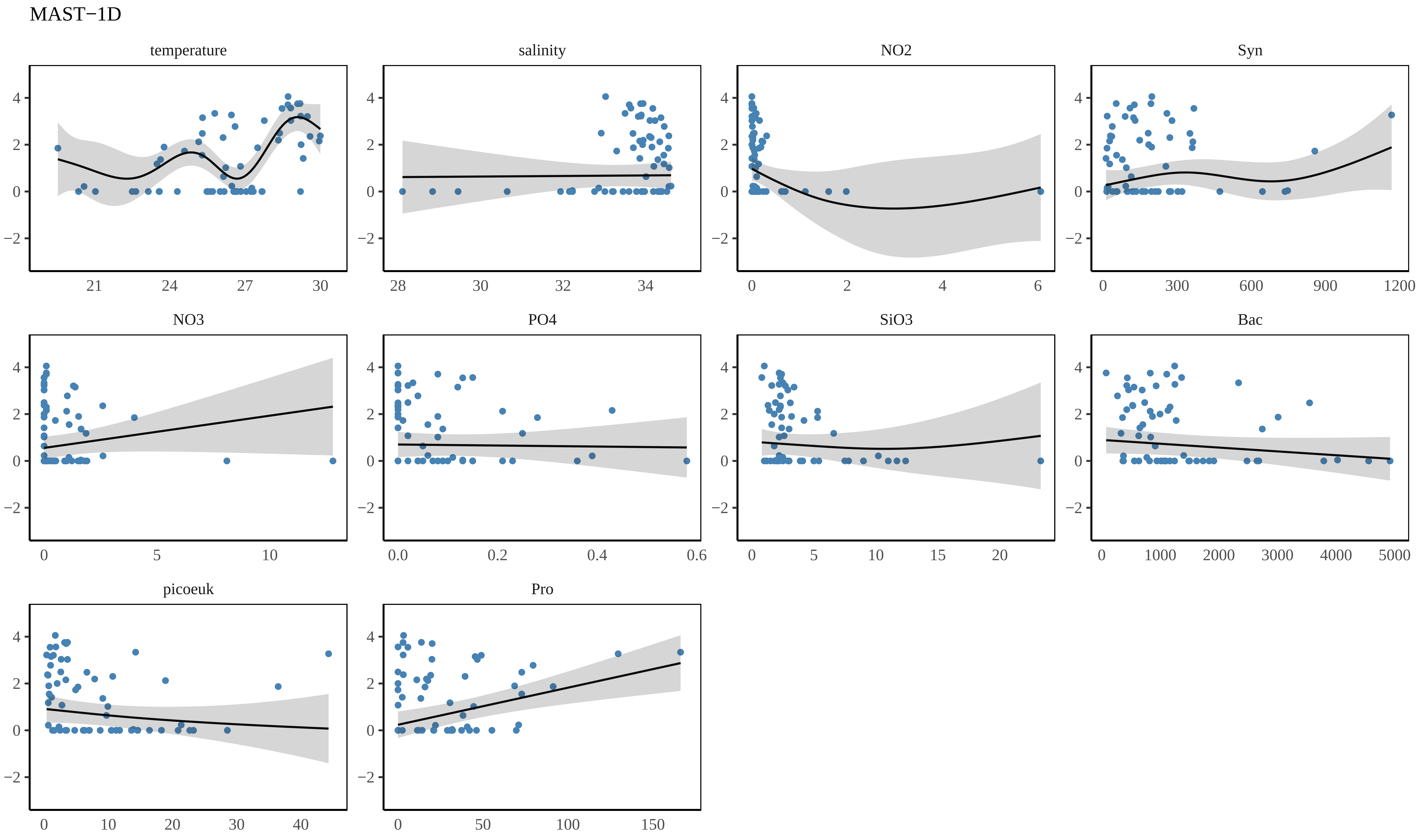

MAST−1D
temperature
salinity
NO2
Syn
NO3
PO4
SiO3
Bac
picoeuk
Pro
4
2
0
−2
21
24
27
30
28
30
32
34
0
2
4
6
0
300
600
900
1200
0
5
10
0.0
0.2
0.4
0.6
0
5
10
15
20
0
1000
2000
3000
4000
5000
0
10
20
30
40
0
50
100
150

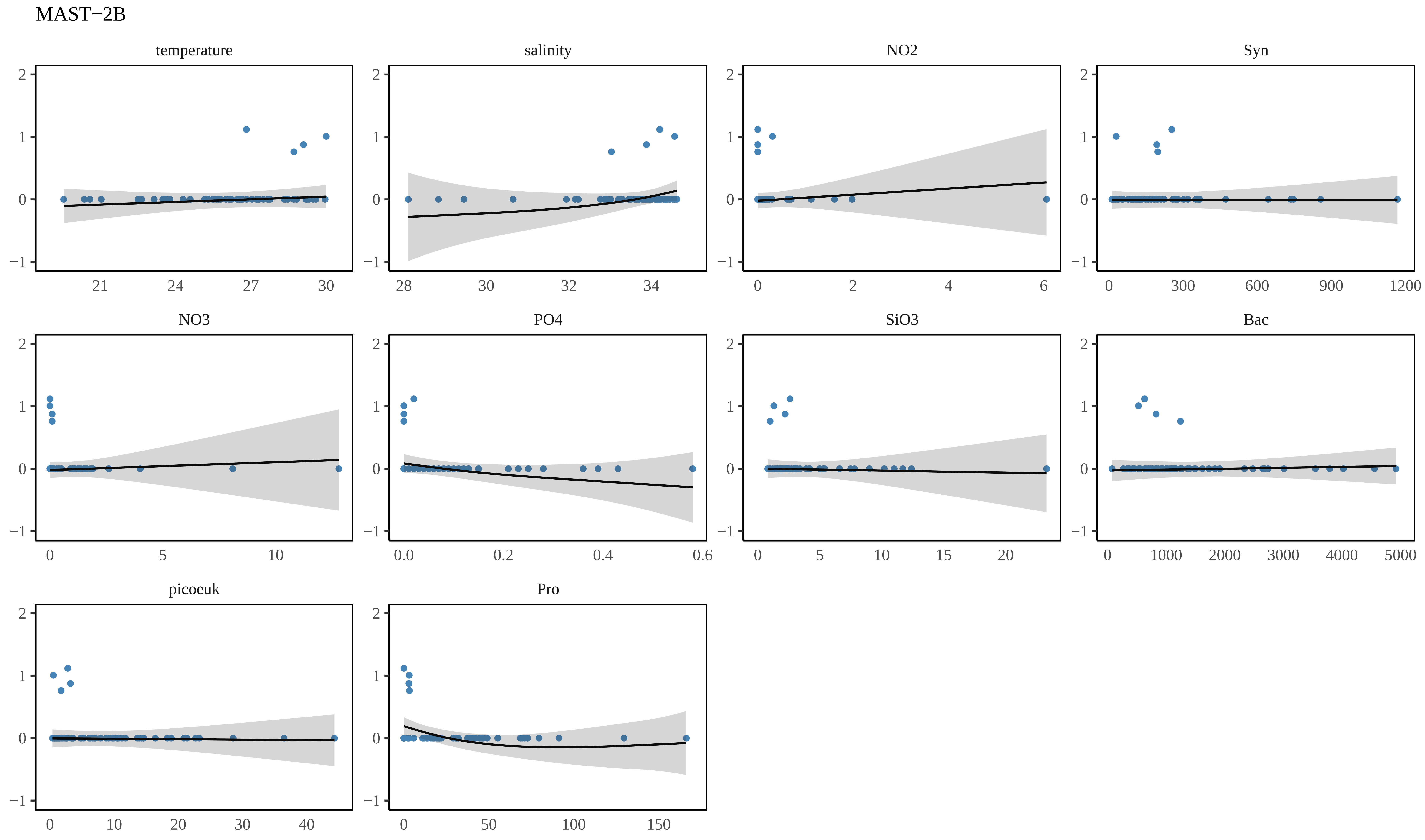

MAST−2B
temperature
salinity
NO2
Syn
NO3
PO4
SiO3
Bac
picoeuk
Pro
2
1
0
−1
21
24
27
30
28
32
34
0
2
4
6
300
600
900
1200
5
10
0.0
0.2
0.4
0.6
15
20
1000
2000
3000
4000
5000
40
50
100
150

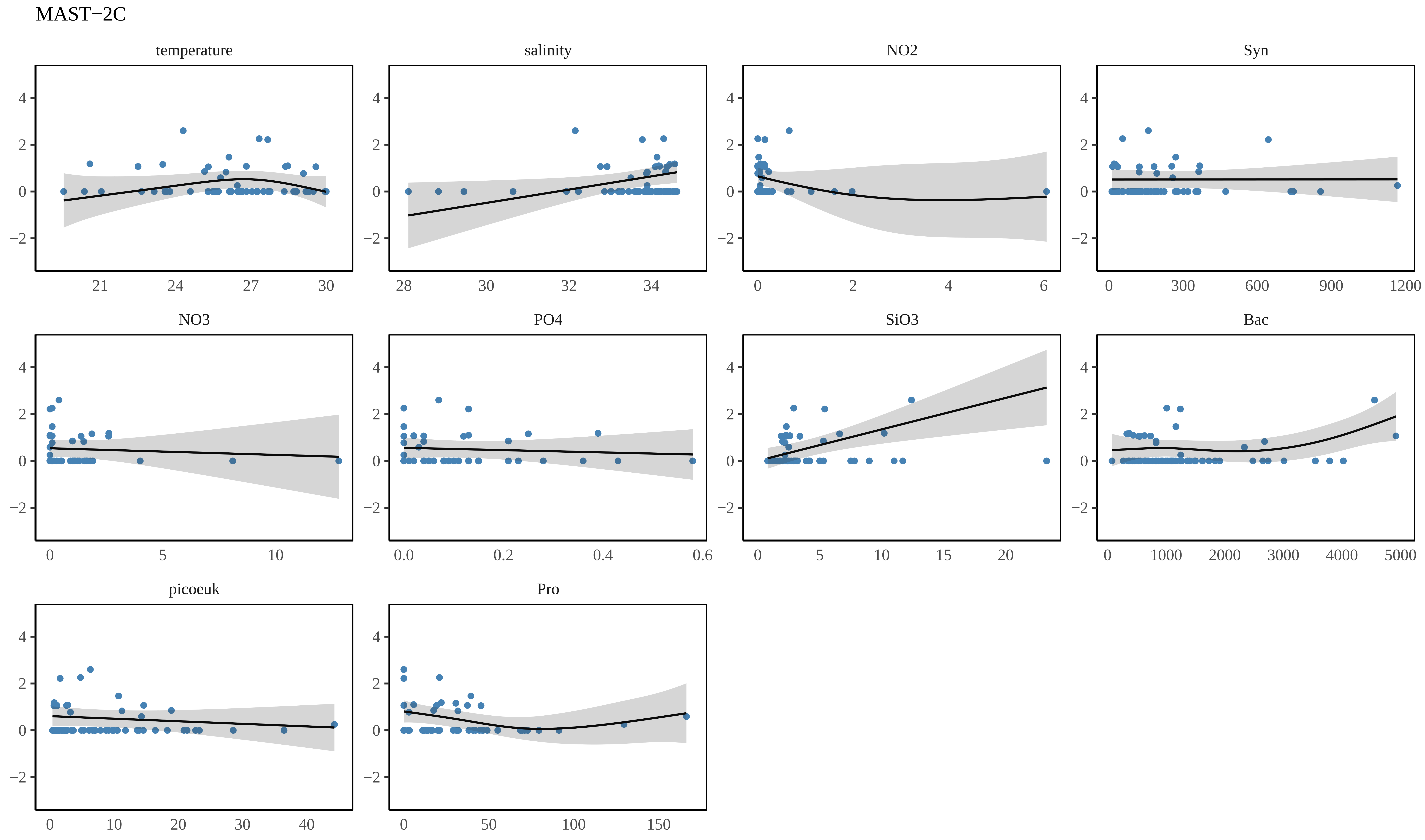
MAST−2C
temperature
salinity
NO2
Syn
NO3
PO4
SiO3
Bac
picoeuk
Pro
4
2
0
−2
21
24
27
30
28
30
32
34
0
2
4
6
0
300
600
900
1200
0
5
10
0.0
0.2
0.4
0.6
0
5
10
15
20
0
1000
2000
3000
4000
5000
0
10
20
30
40
0
50
100
150

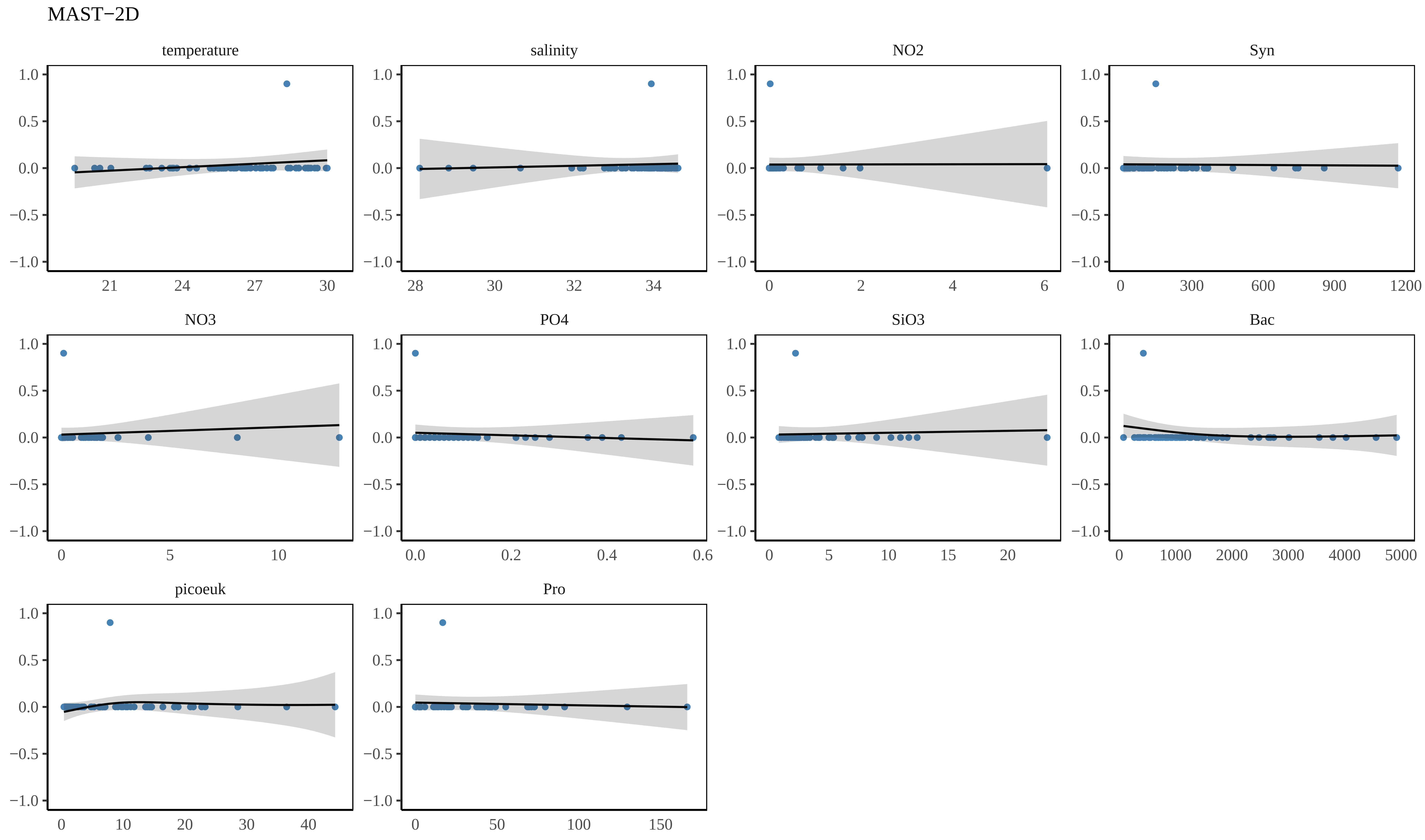
MAST−2D
temperature
salinity
NO2
Syn
NO3
PO4
SiO3
Bac
picoeuk
Pro
1.0
0.5
0.0
−0.5
−1.0
21
24
27
30
28
30
32
34
0
2
4
6
0
300
600
900
1200
0
5
10
0.0
0.2
0.4
0.6
0
5
10
15
20
0
1000
2000
3000
4000
5000
0
10
20
30
40
0
50
100
150

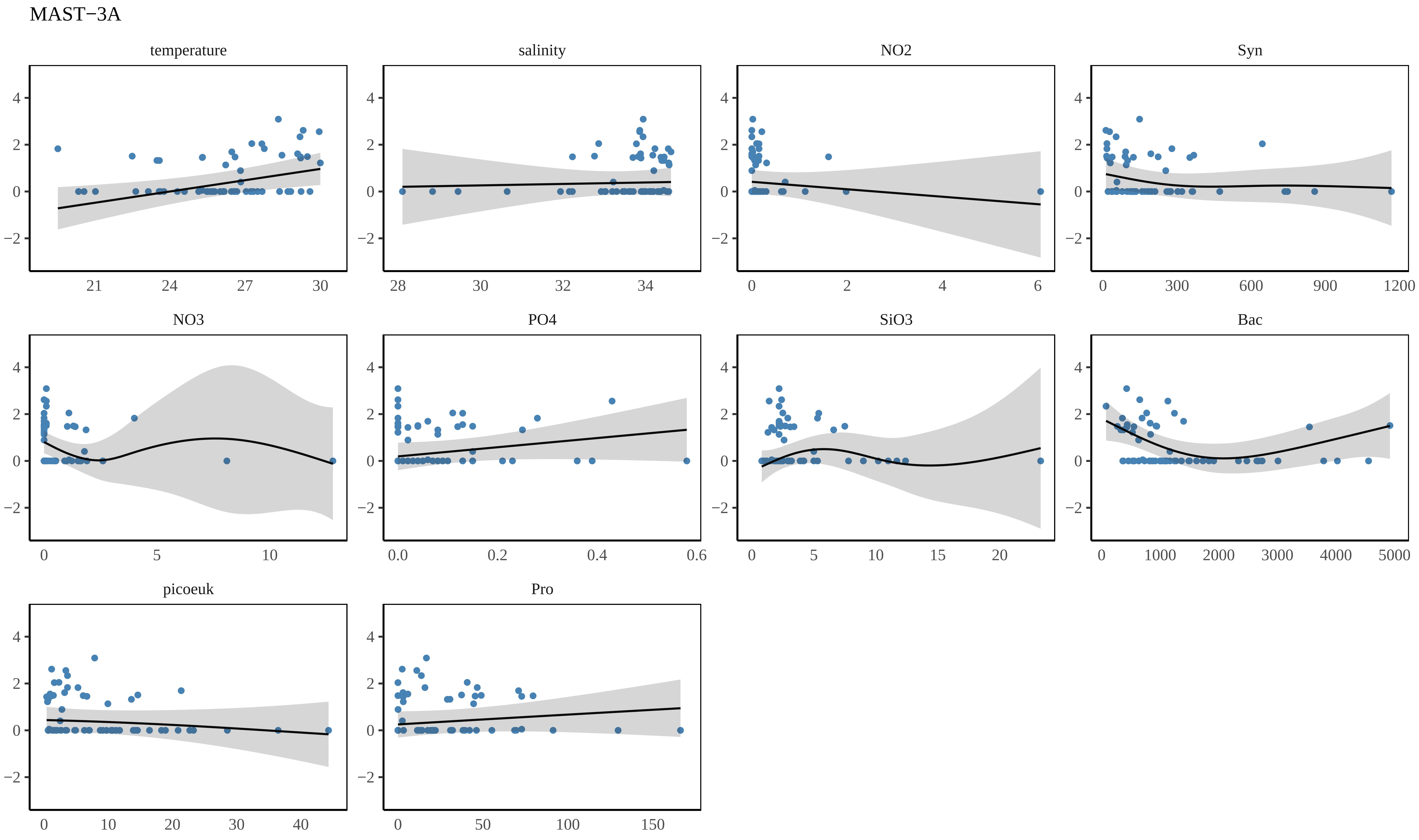

MAST−3A
temperature
salinity
NO2
Syn
NO3
PO4
SiO3
Bac
picoeuk
Pro

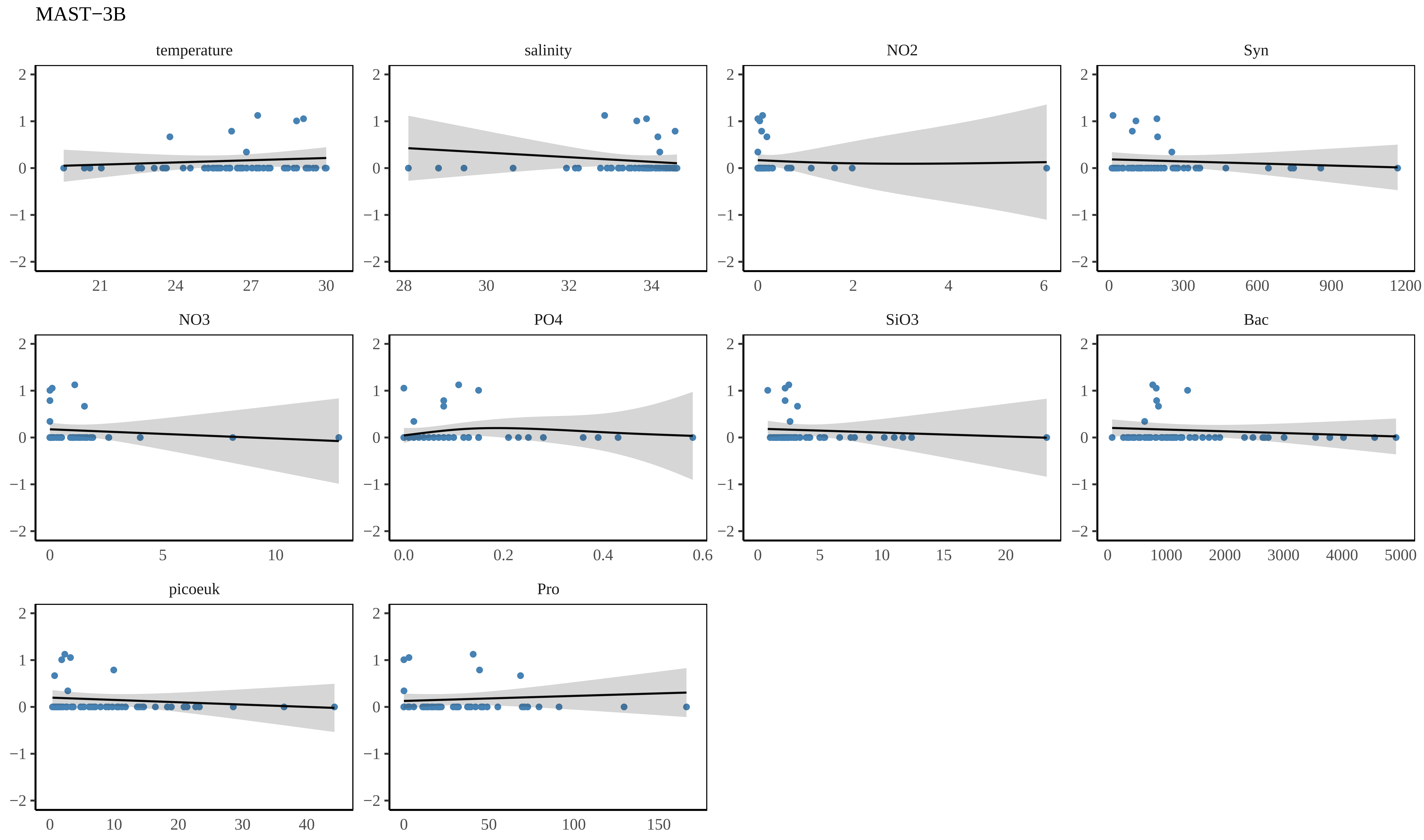

MAST−3B
temperature
salinity
NO2
Syn
NO3
PO4
SiO3
Bac
picoeuk
Pro
2
1
0
−1
−2
21
24
27
30
28
30
32
34
0
2
4
6
0
300
600
900
1200
0
5
10
0.0
0.2
0.4
0.6
0
5
10
15
20
0
1000
2000
3000
4000
5000
0
10
20
30
40
0
50
100
150

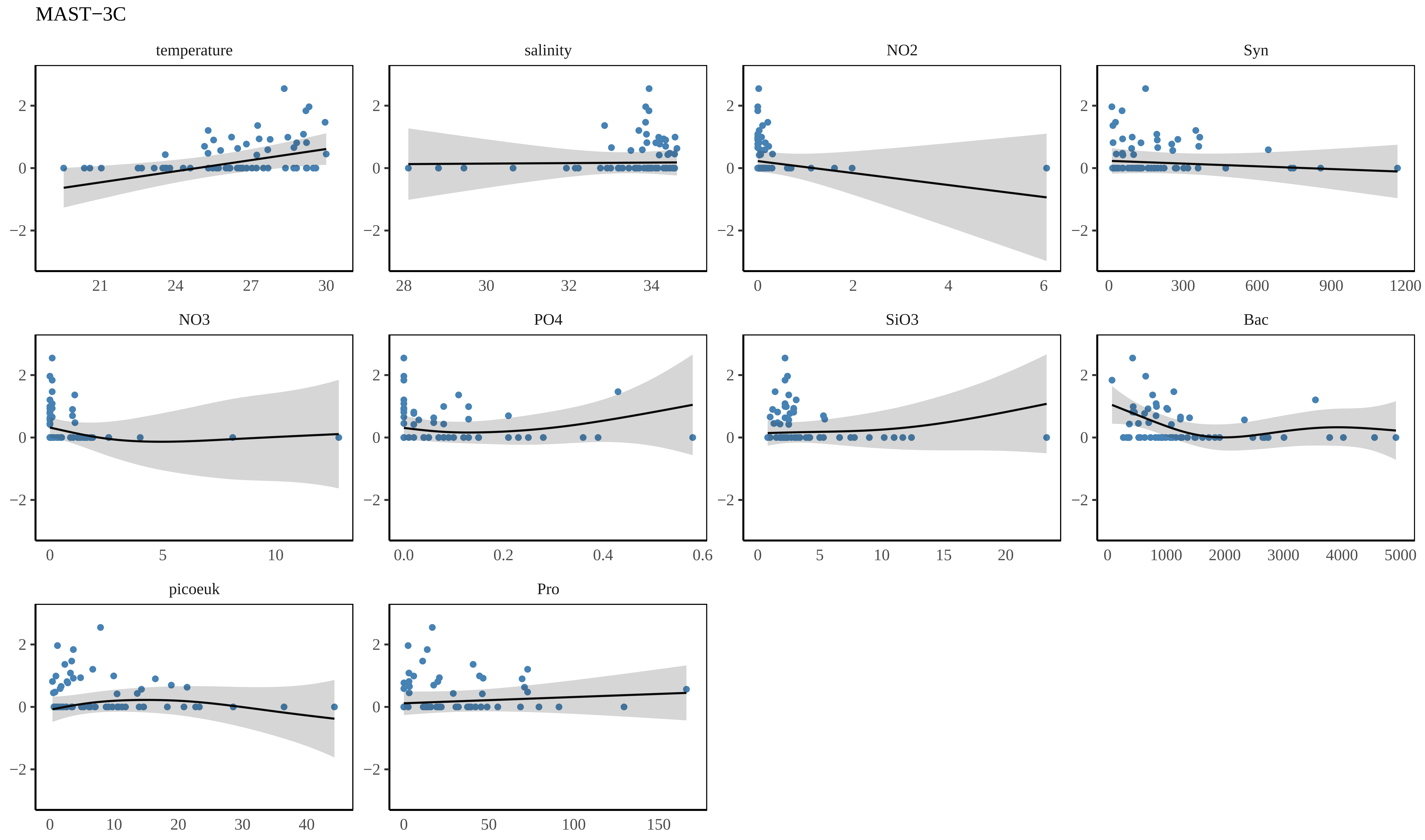

MAST−3C
temperature
salinity
NO2
Syn
NO3
PO4
SiO3
Bac
picoeuk
Pro
2
0
−2
21
24
27
30
28
30
32
34
0
2
4
6
0
300
600
900
1200
0
5
10
0.0
0.2
0.4
0.6
0
5
10
15
20
0
1000
2000
3000
4000
5000
0
10
20
30
40
0
50
100
150

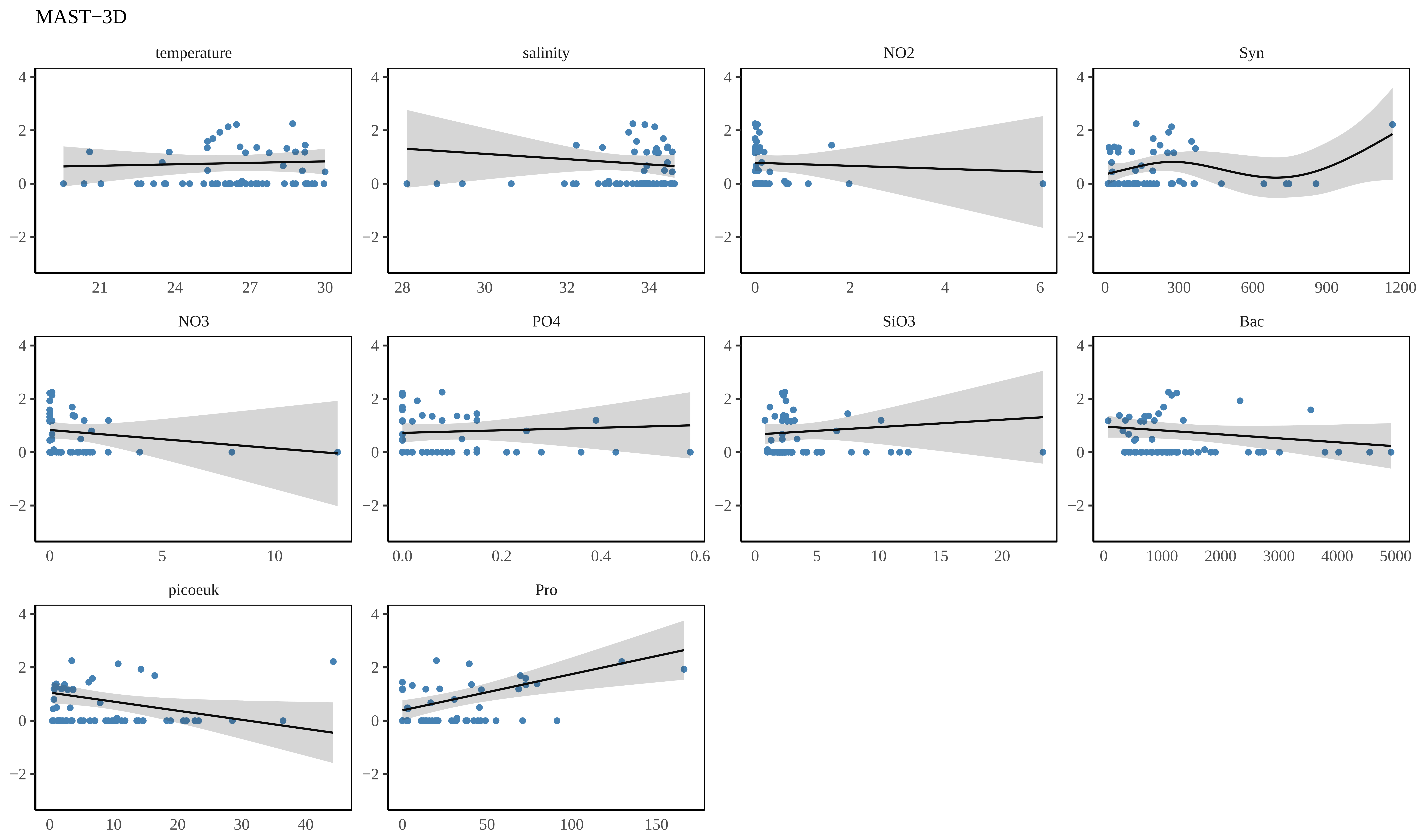

MAST−3D
temperature
salinity
NO2
Syn
NO3
PO4
SiO3
Bac
picoeuk
Pro

MAST−3E

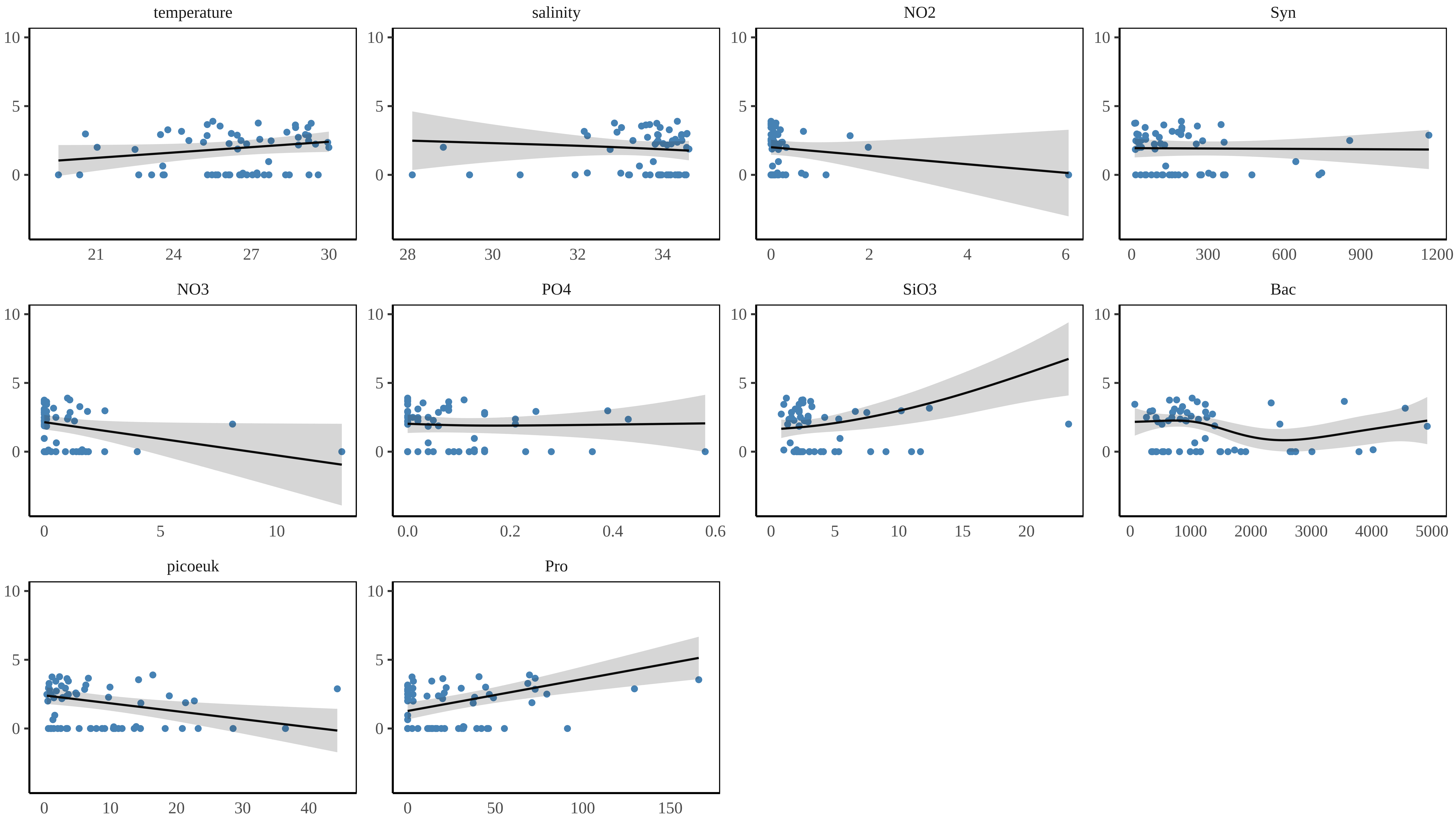
temperature
salinity
NO2
Syn
NO3
PO4
SiO3
Bac
picoeuk
Pro
10
5
0
21
24
27
30
28
30
32
34
0
2
4
6
0
300
600
900
1200
0
5
10
0.0
0.2
0.4
0.6
0
5
10
15
20
0
1000
2000
3000
4000
5000
0
10
20
30
40
0
50
100
150

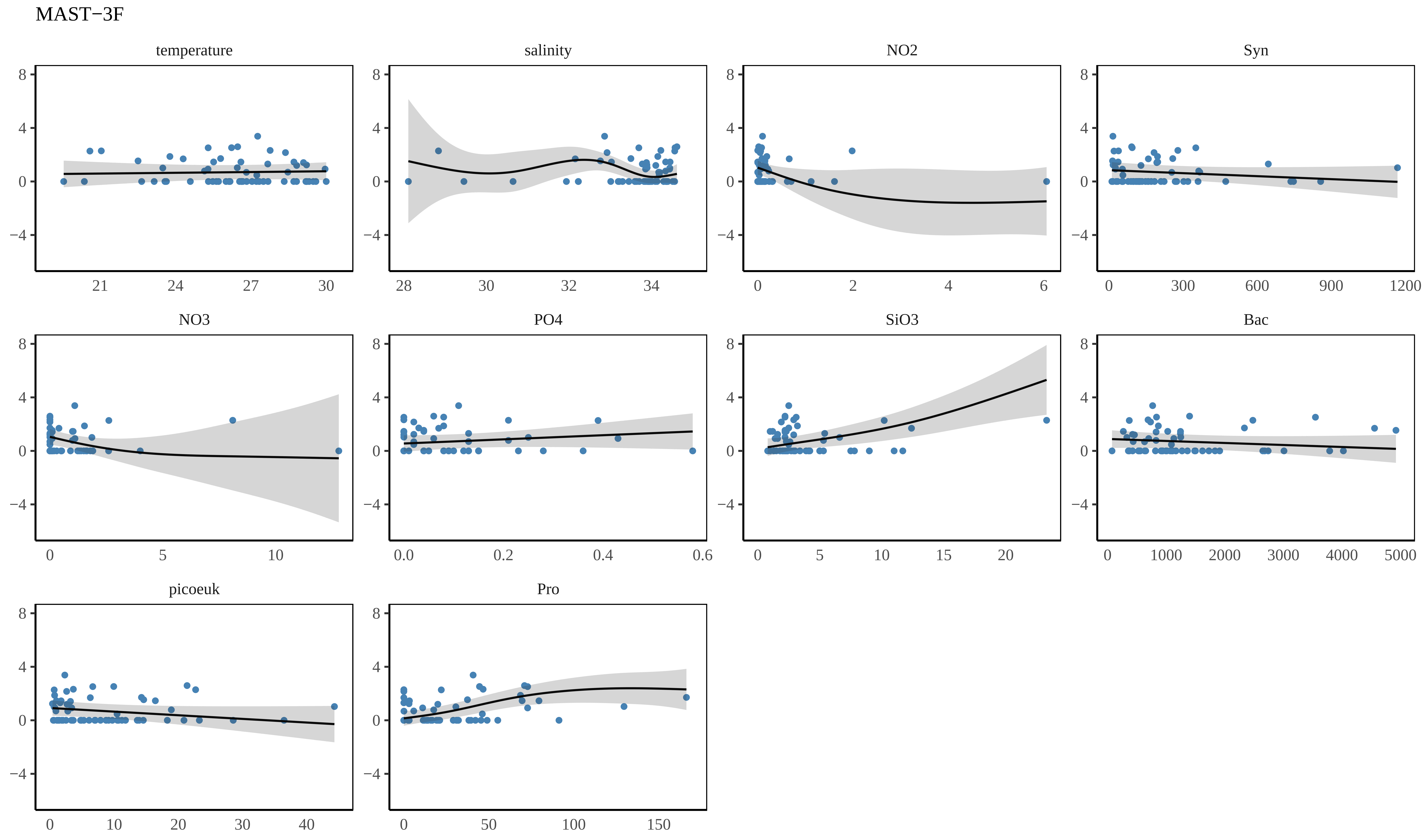

MAST−3F
temperature
salinity
NO2
Syn
NO3
PO4
SiO3
Bac
picoeuk
Pro
8
4
0
−4
21
24
27
30
28
30
32
34
0
2
4
6
0
300
600
900
1200
0
5
10
0.0
0.2
0.4
0.6
0
5
10
15
20
0
1000
2000
3000
4000
5000
0
10
20
30
40
0
50
100
150

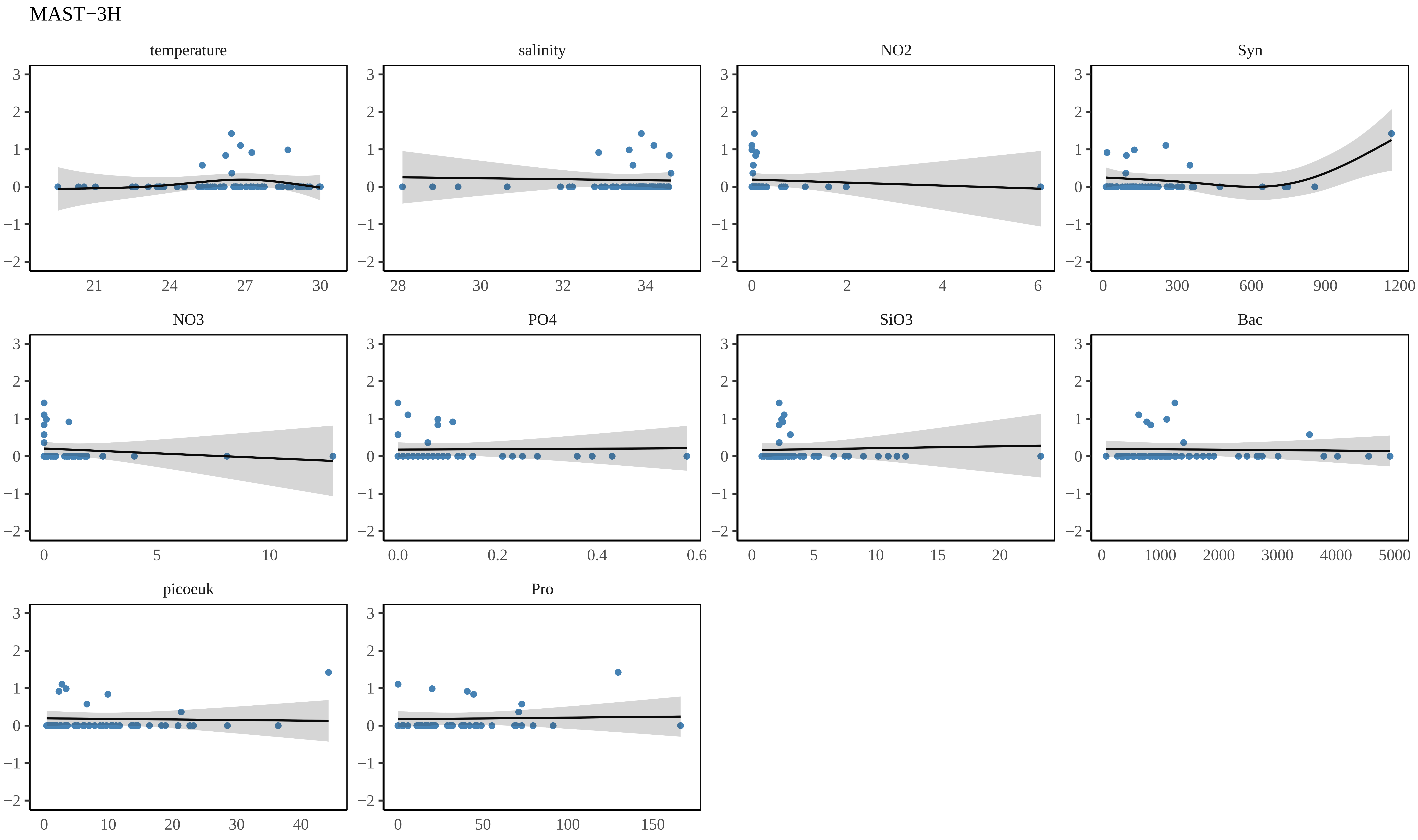
MAST−3H
temperature
salinity
NO2
Syn
NO3
PO4
SiO3
Bac
picoeuk
Pro

MAST−3I

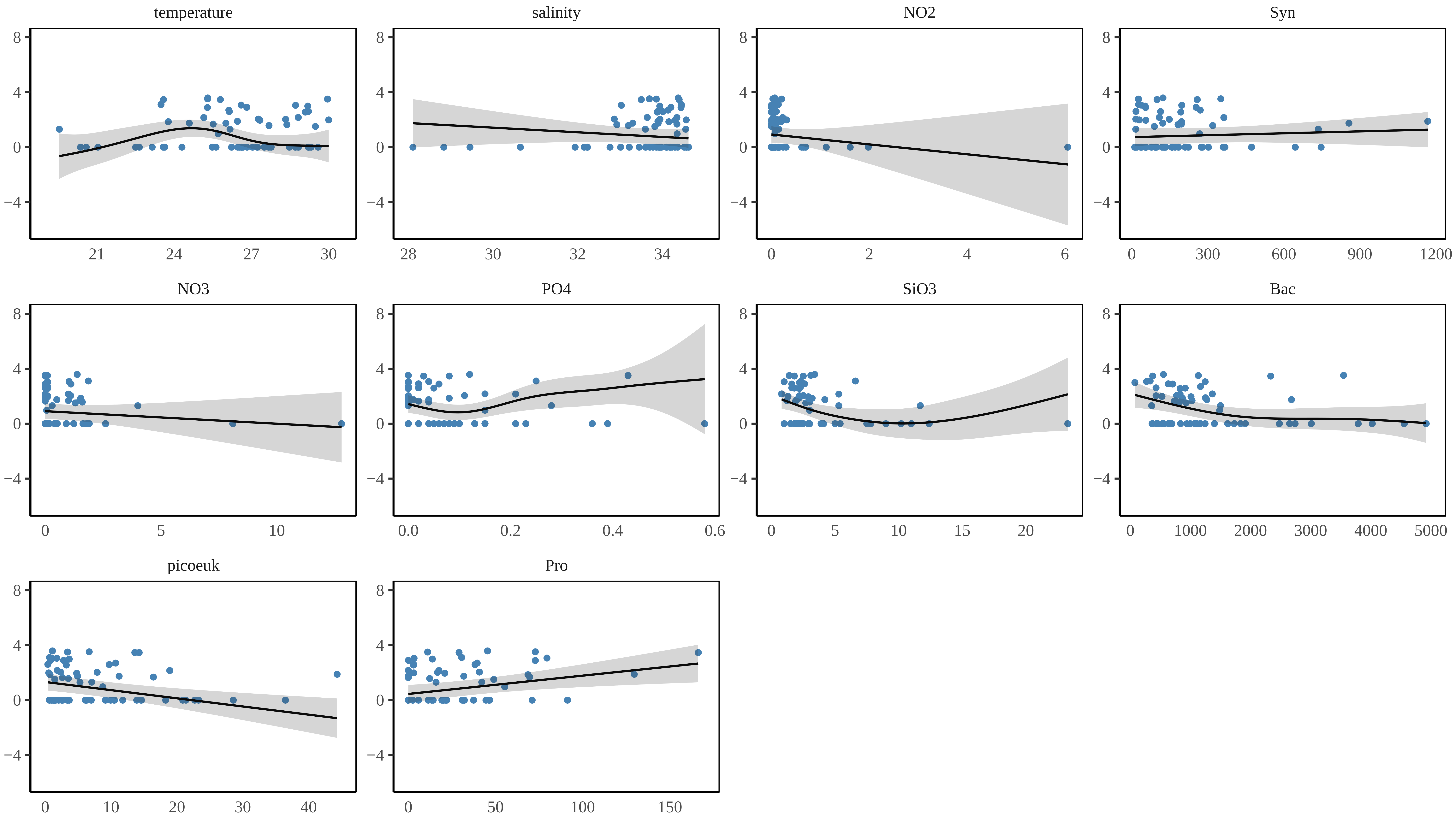

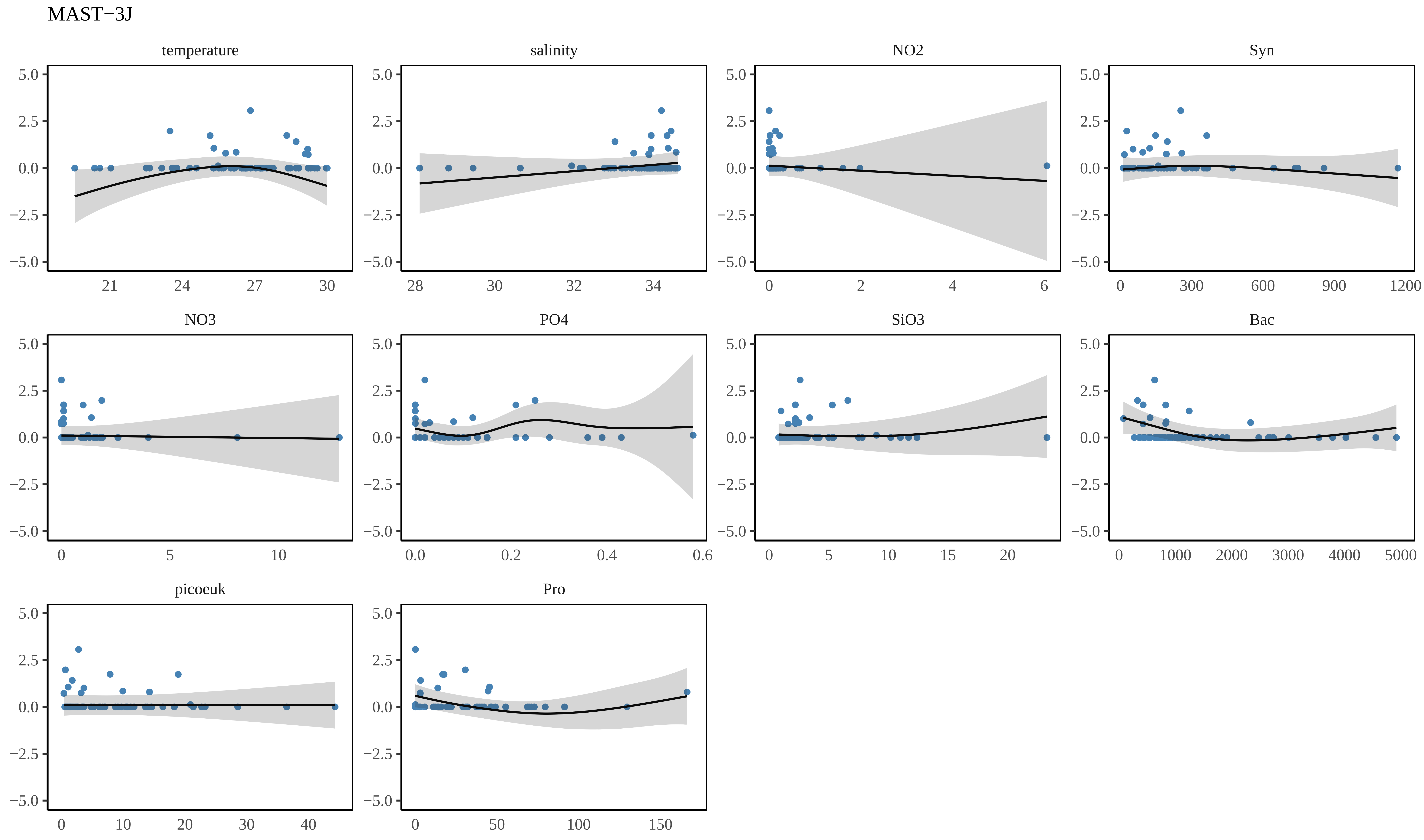
MAST−3J
temperature
salinity
NO2
Syn
NO3
PO4
SiO3
Bac
picoeuk
Pro
5.0
2.5
0.0
−2.5
−5.0
21
24
27
30
28
30
32
34
0
2
4
6
0
300
600
900
1200
0
5
10
0.0
0.2
0.4
0.6
0
5
10
15
20
0
1000
2000
3000
4000
5000
0
10
20
30
40
0
50
100
150

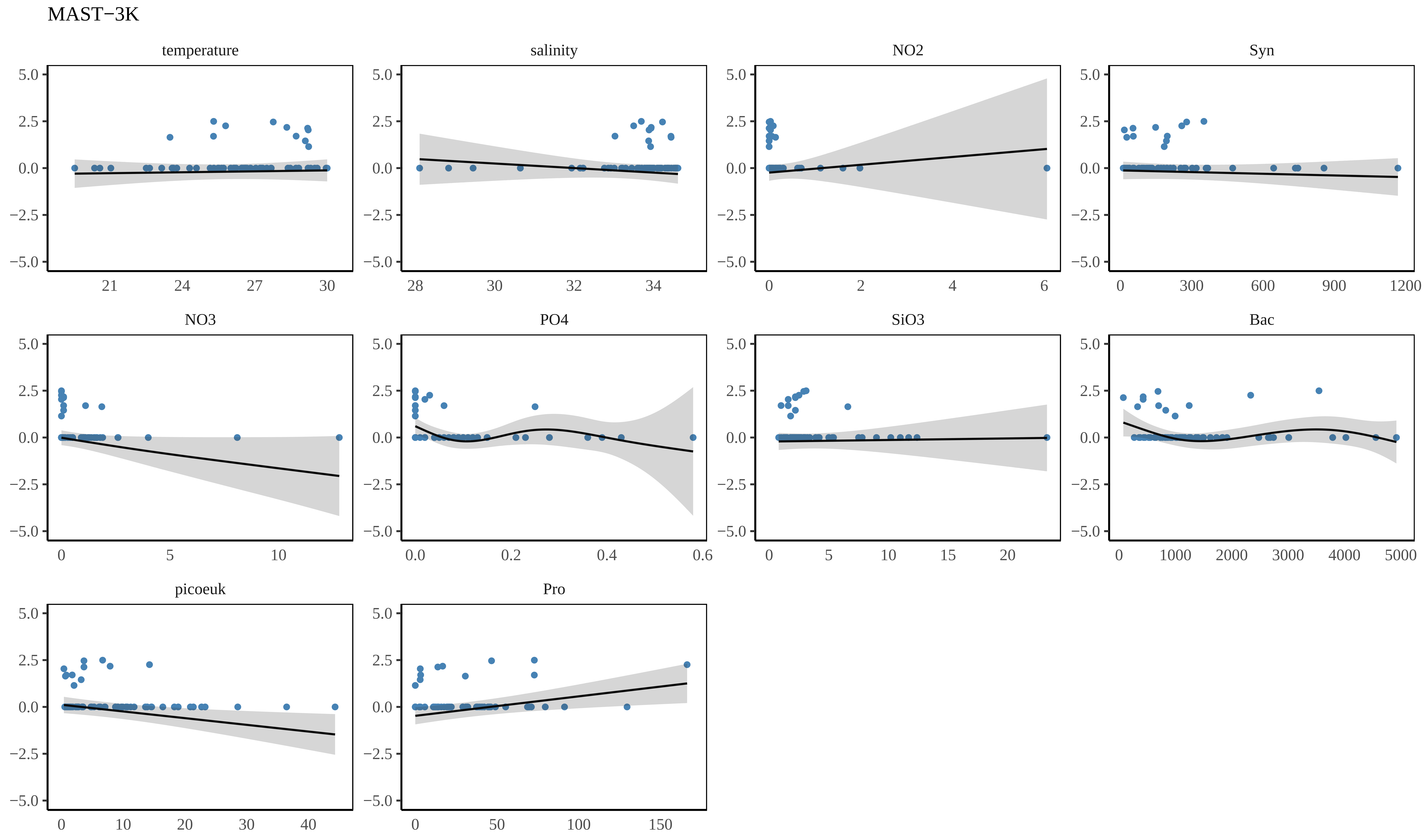
MAST−3K
temperature
salinity
NO2
Syn
NO3
PO4
SiO3
Bac
picoeuk
Pro
5.0
2.5
0.0
−2.5
−5.0
21
24
27
30
28
32
34
0
2
4
6
300
600
900
1200
5
10
0.2
0.4
0.6
15
20
1000
2000
3000
4000
5000
40
50
100
150

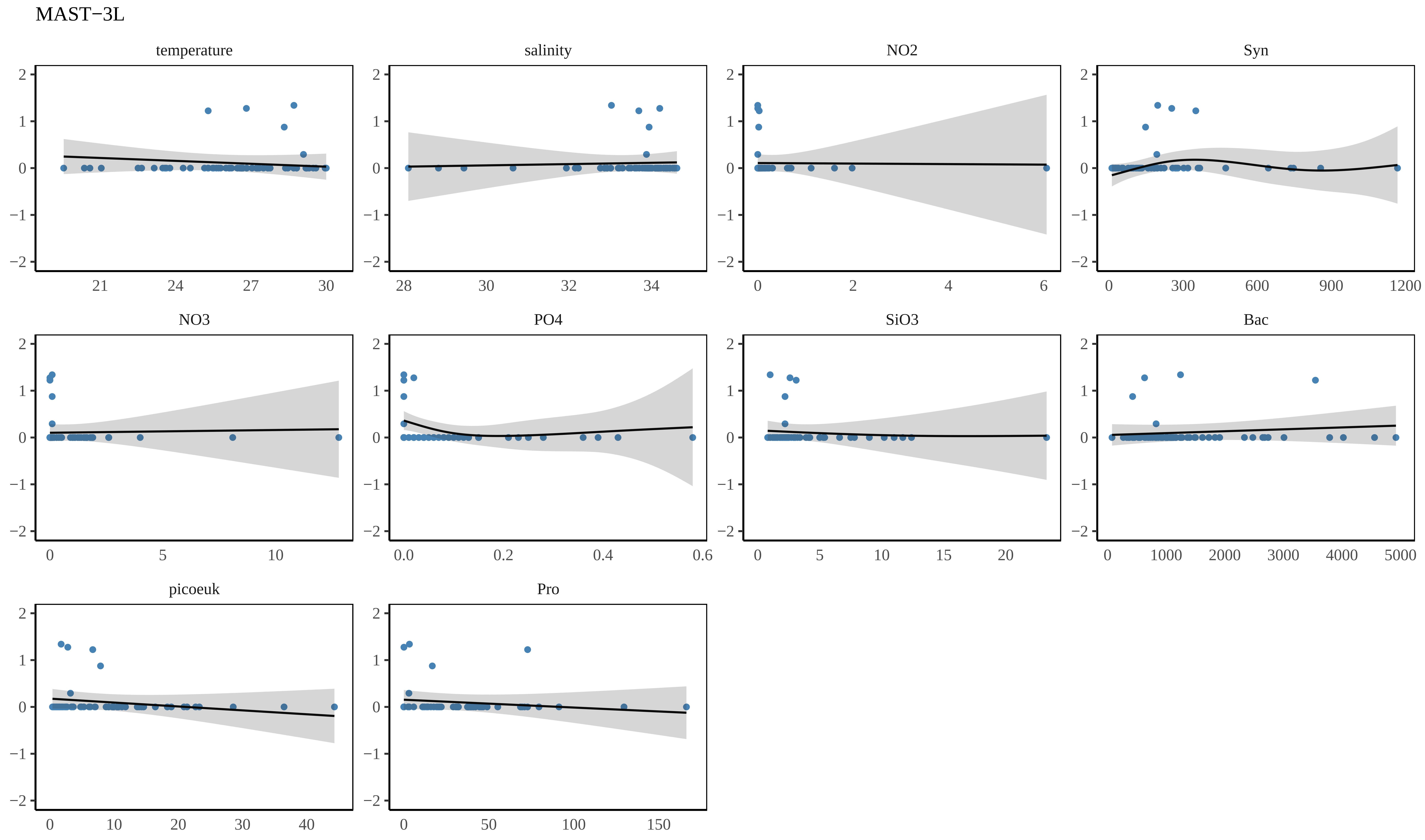

MAST−3L
temperature
salinity
NO2
Syn
NO3
PO4
SiO3
Bac
picoeuk
Pro

MAST−3

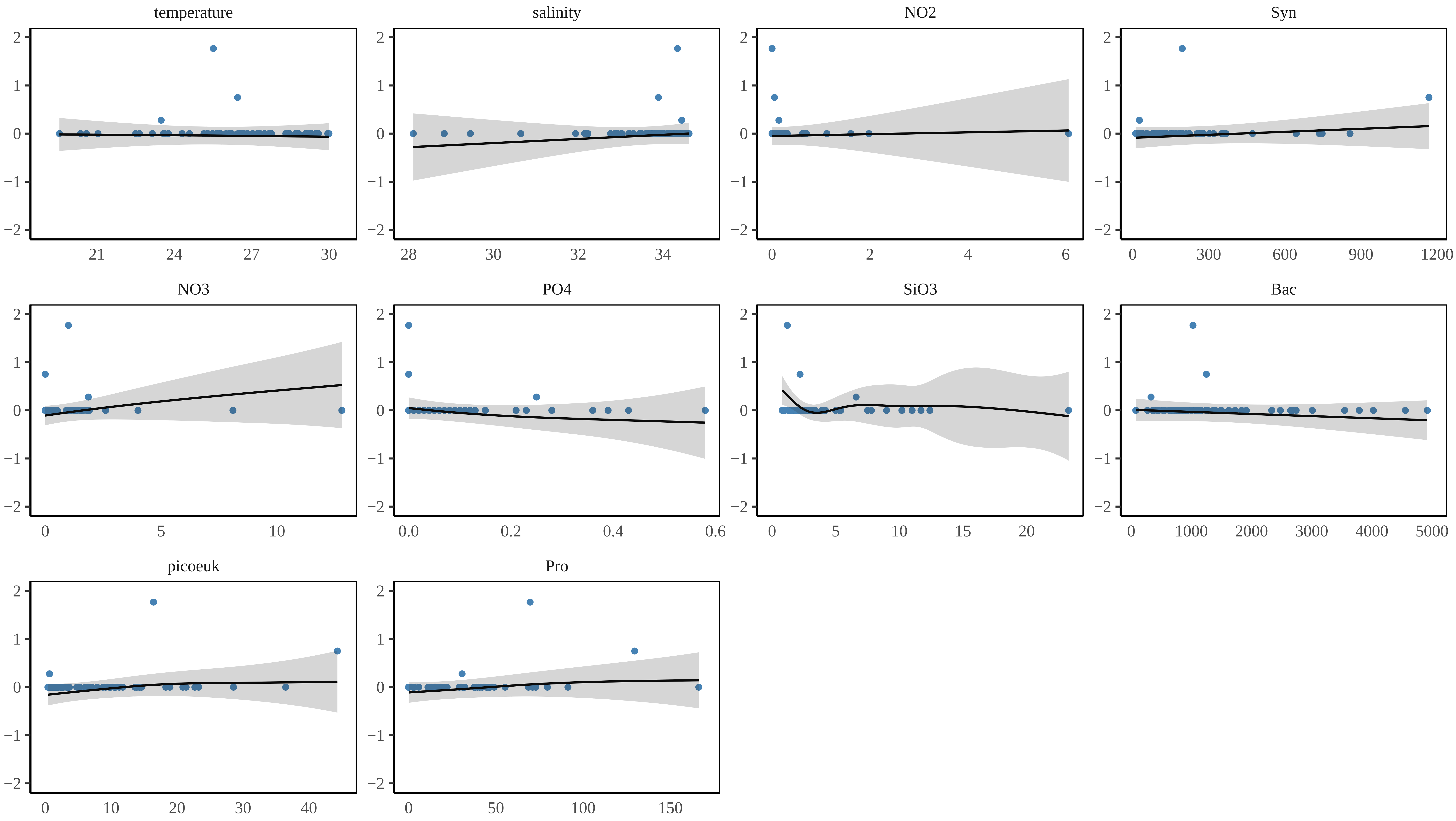
temperature
salinity
NO2
Syn
NO3
PO4
SiO3
Bac
picoeuk
Pro
2
1
0
−1
−2
21
24
27
30
28
30
32
34
0
2
4
6
0
300
600
900
1200
0
5
10
0.0
0.2
0.4
0.6
0
5
10
15
20
0
1000
2000
3000
4000
5000
0
10
20
30
40
0
50
100
150

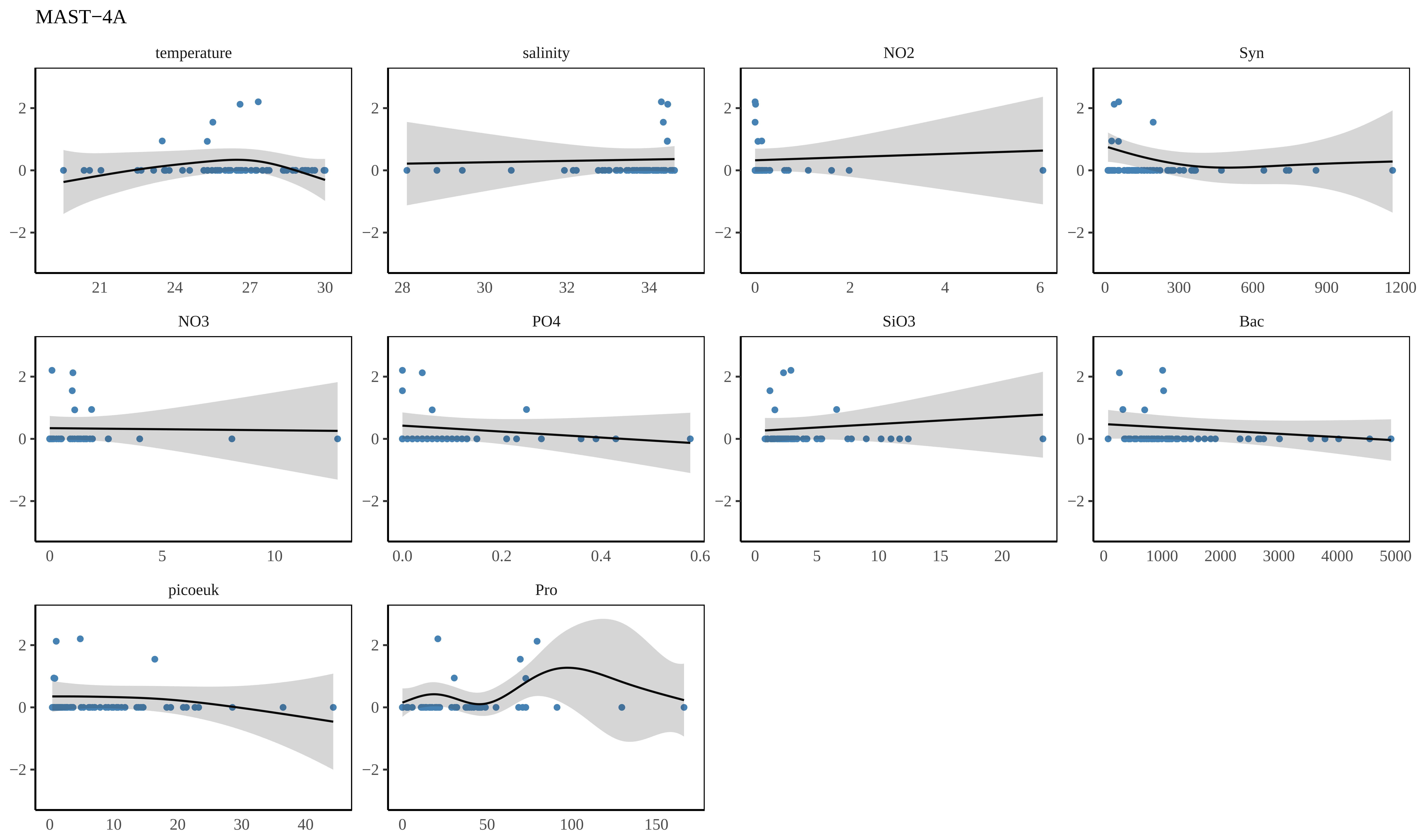
MAST−4A
temperature
salinity
NO2
Syn
NO3
PO4
SiO3
Bac
picoeuk
Pro

MAST−4B

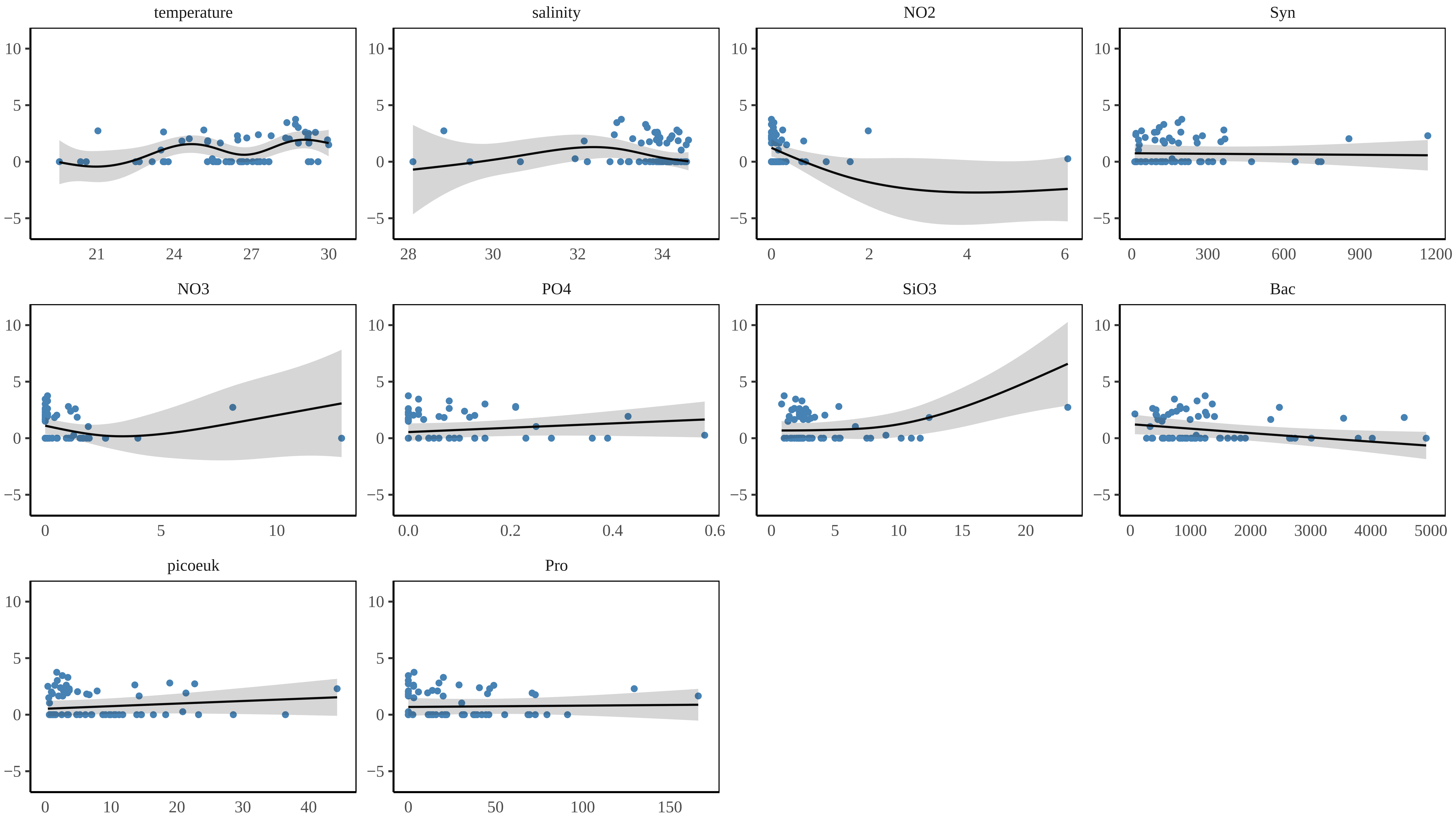

temperature
salinity
NO2
Syn
NO3
PO4
SiO3
Bac
picoeuk
Pro
10
5
0
−5
21
24
27
30
28
30
32
34
0
2
4
6
0
300
600
900
1200
0
5
10
0.0
0.2
0.4
0.6
0
5
10
15
20
0
1000
2000
3000
4000
5000
0
10
20
30
40
0
50
100
150

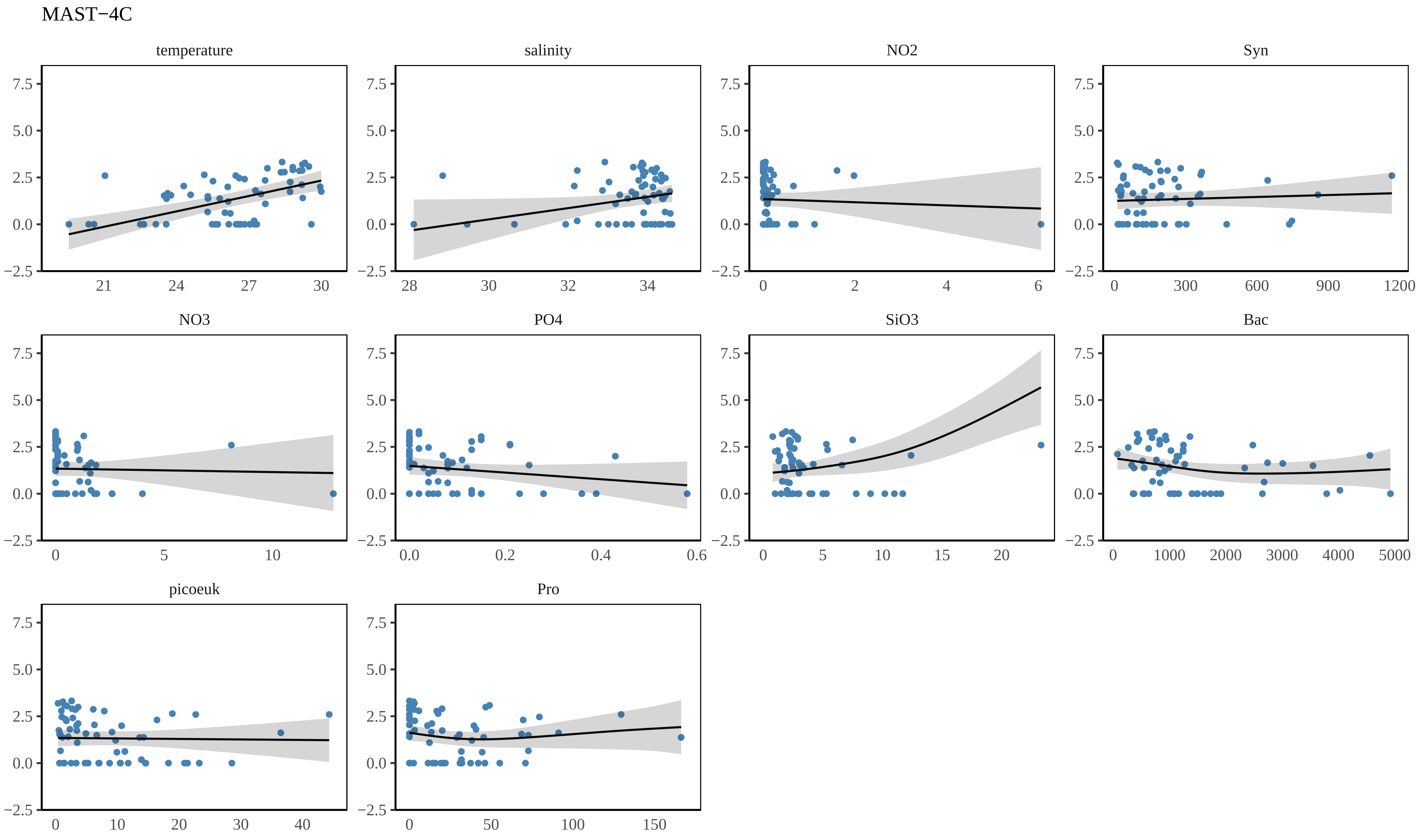
MAST−4C
temperature
salinity
NO2
Syn
NO3
PO4
SiO3
Bac
picoeuk
Pro

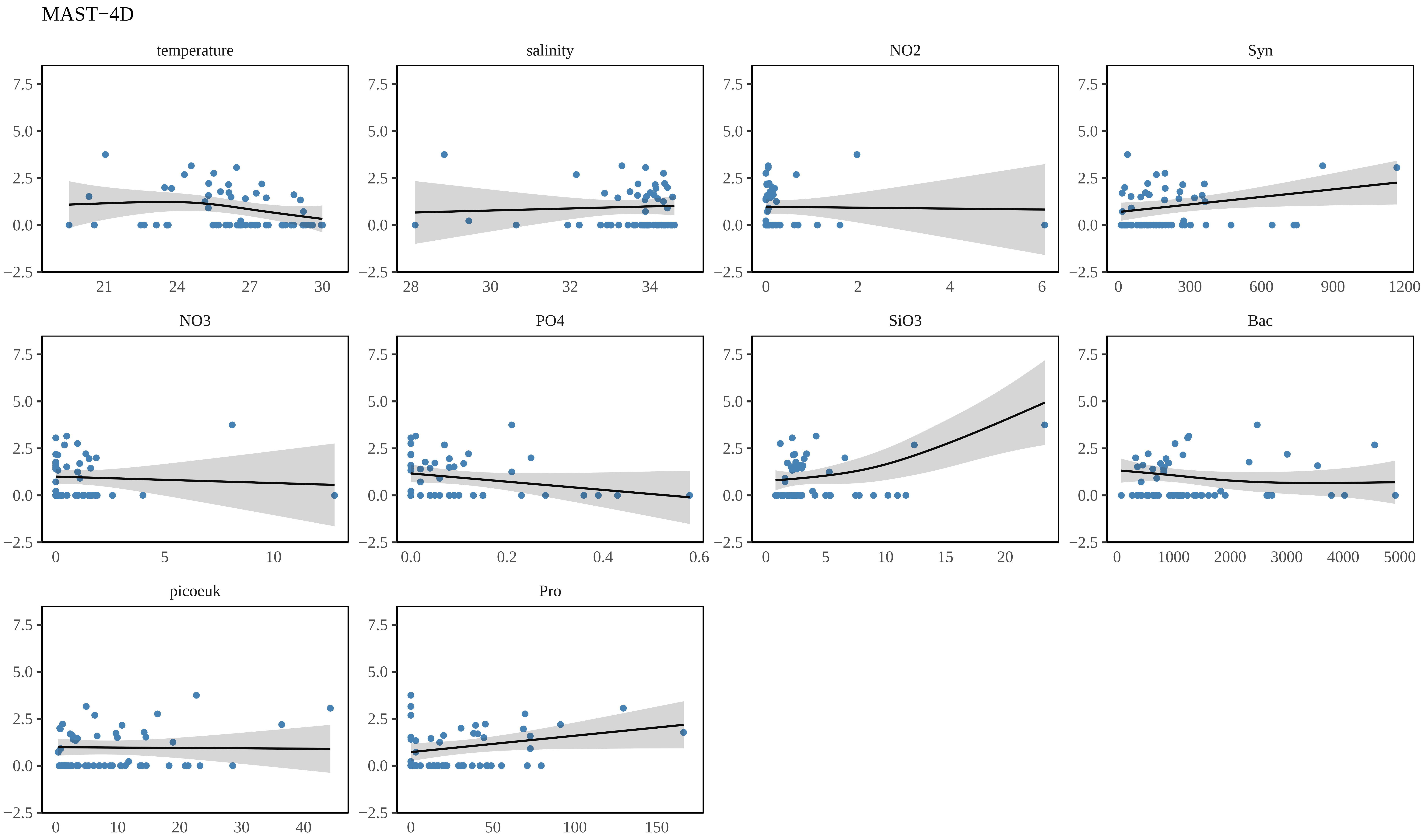

MAST−4D
temperature
salinity
NO2
Syn
NO3
PO4
SiO3
Bac
picoeuk
Pro
7.5
5.0
2.5
0.0
−2.5
21
24
27
30
28
30
32
34
0
2
4
6
0
300
600
900
1200
0
5
10
0.0
0.2
0.4
0.6
0
5
10
15
20
0
1000
2000
3000
4000
5000
0
10
20
30
40
0
50
100
150

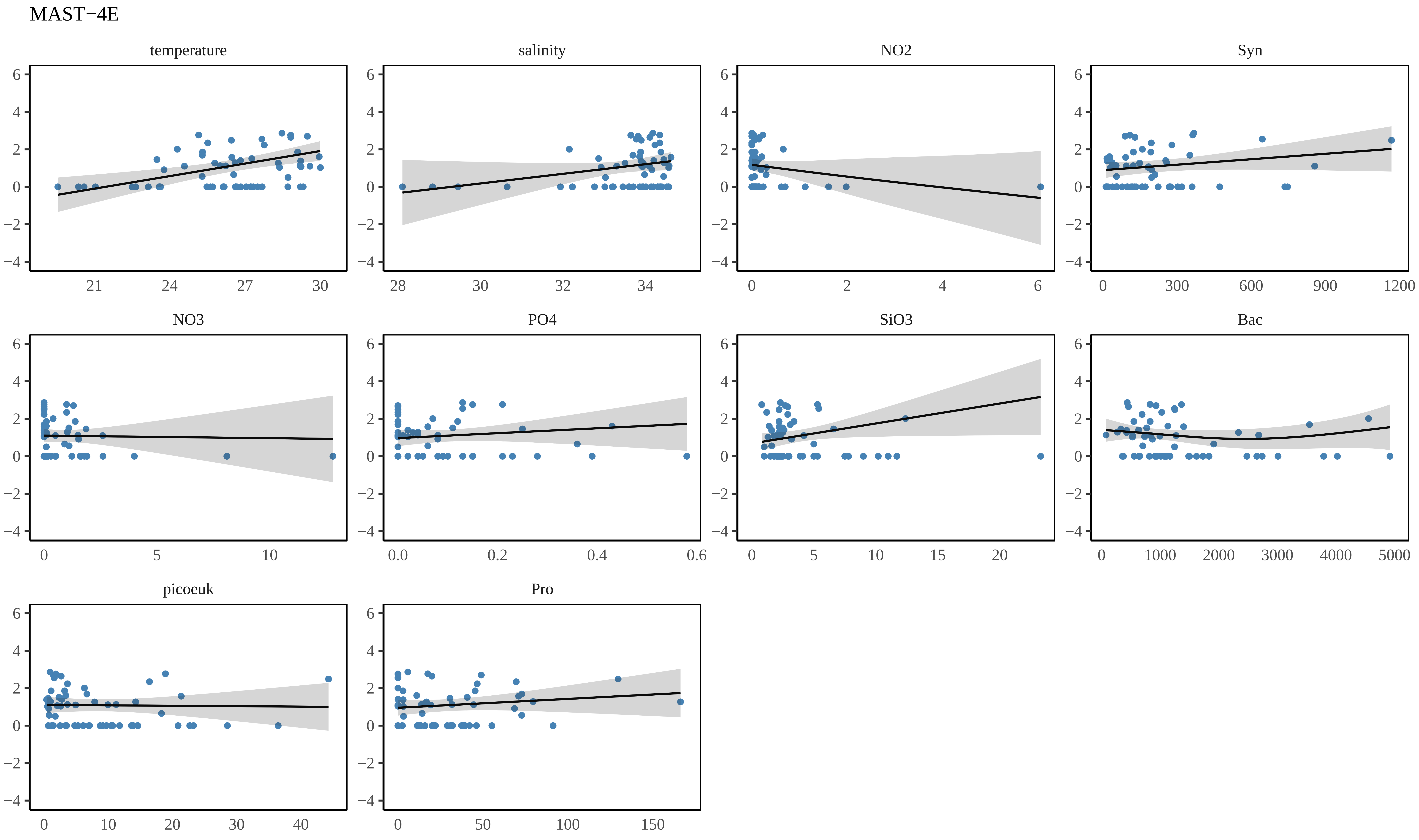
MAST−4E
temperature
salinity
NO2
Syn
NO3
PO4
SiO3
Bac
picoeuk
Pro

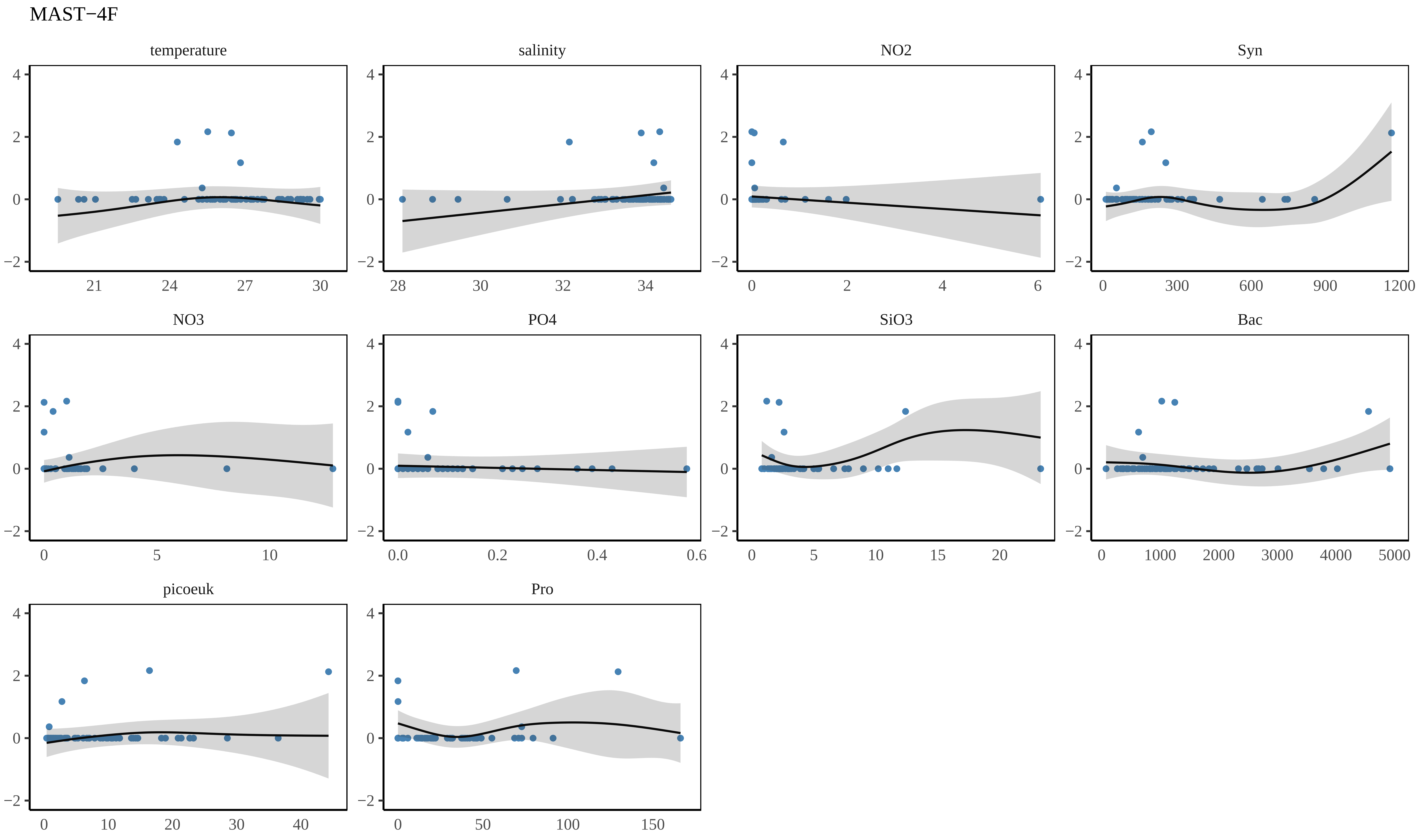

MAST−4F
temperature
salinity
NO2
Syn
NO3
PO4
SiO3
Bac
picoeuk
Pro

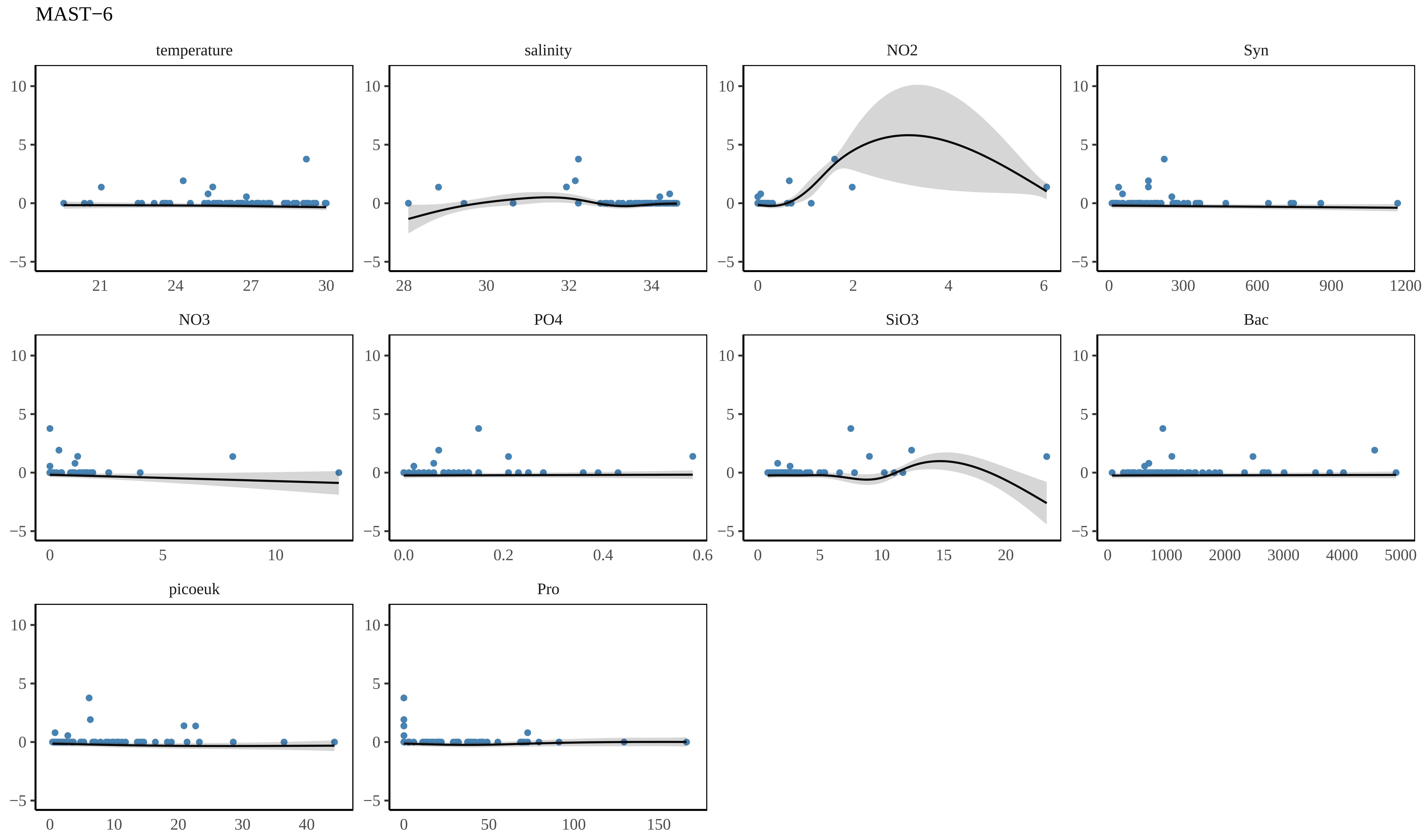
MAST−6
temperature
salinity
NO2
Syn
NO3
PO4
SiO3
Bac
picoeuk
Pro

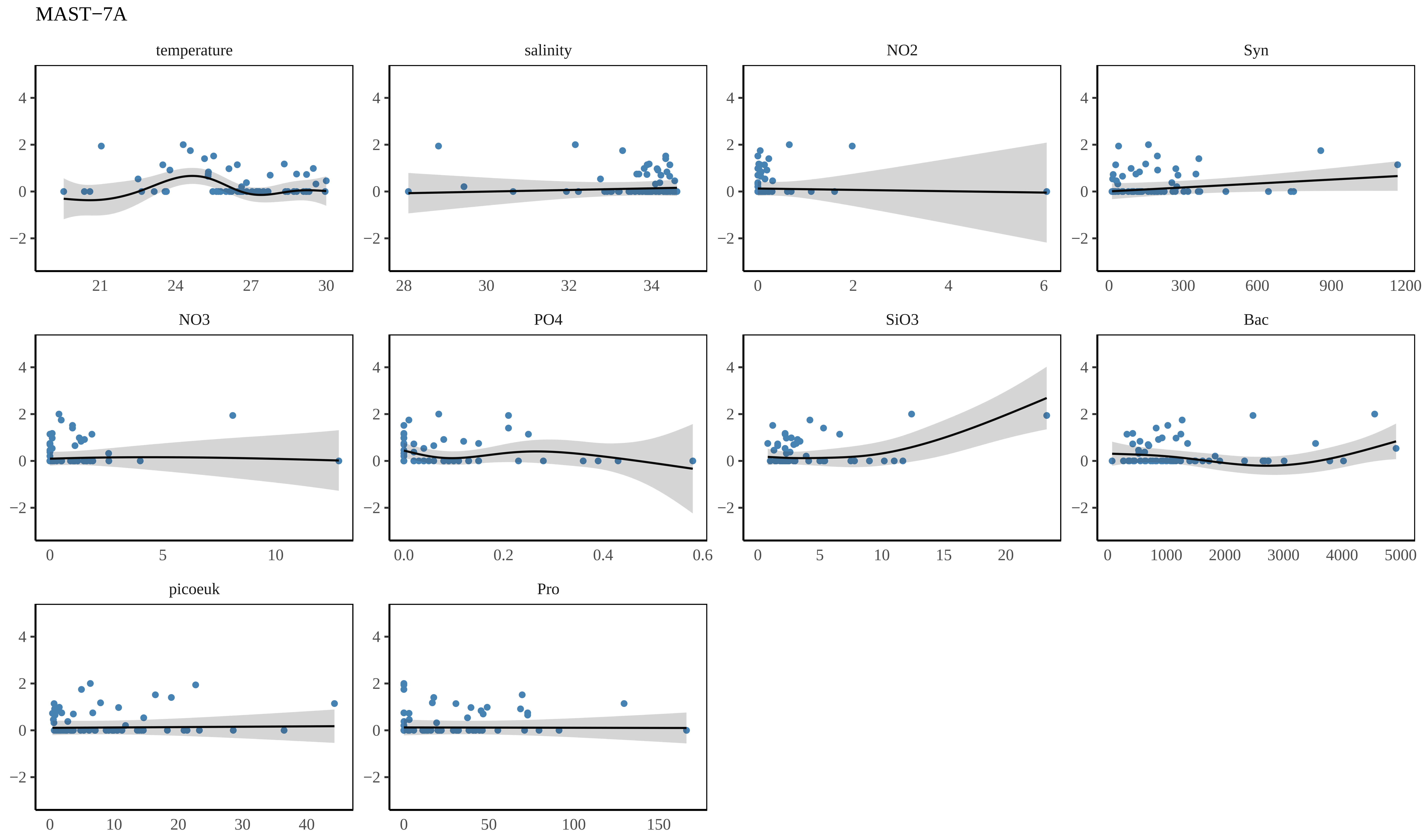

MAST−7A
temperature
salinity
NO2
Syn
NO3
PO4
SiO3
Bac
picoeuk
Pro

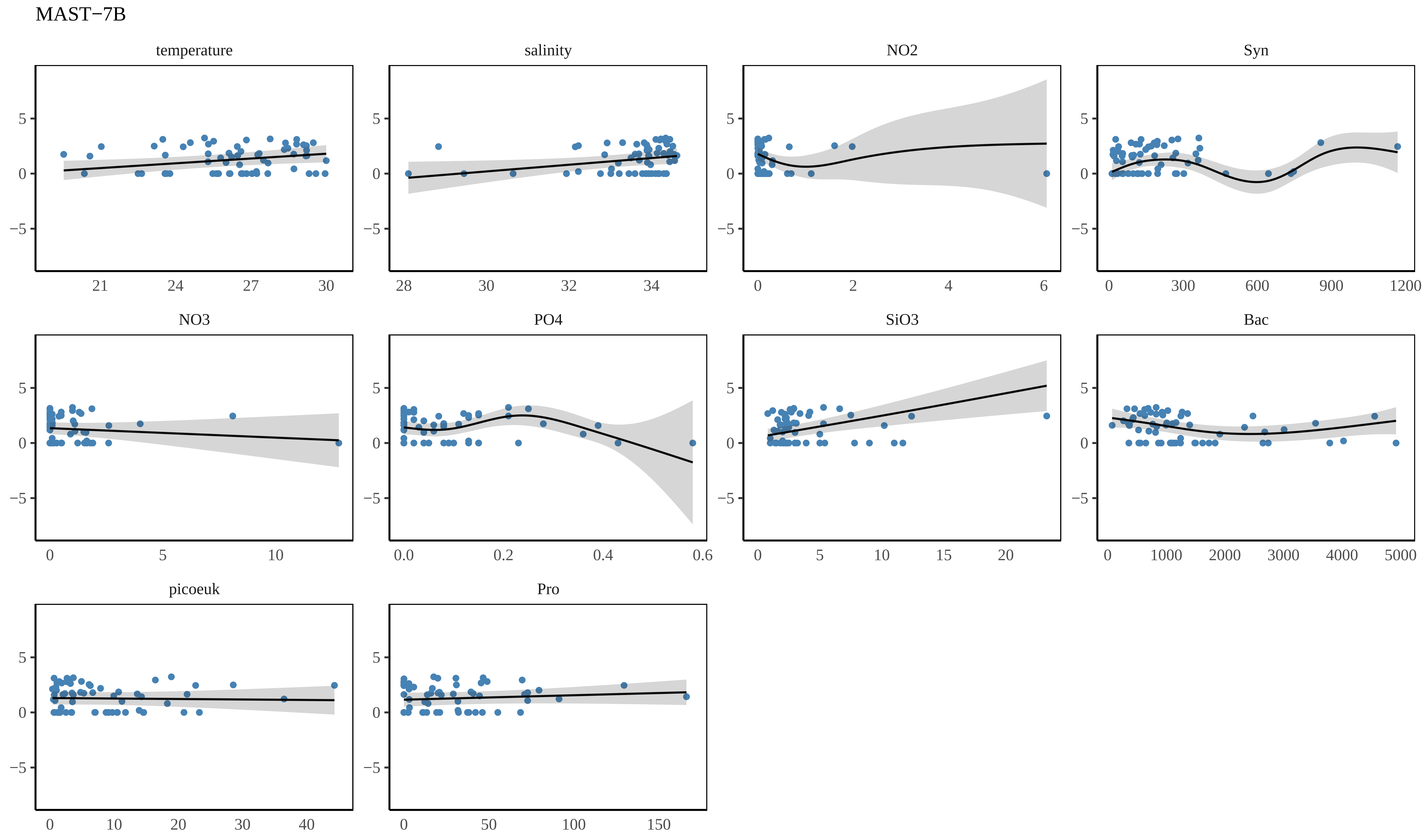

MAST−7B
temperature
salinity
NO2
Syn
NO3
PO4
SiO3
Bac
picoeuk
Pro

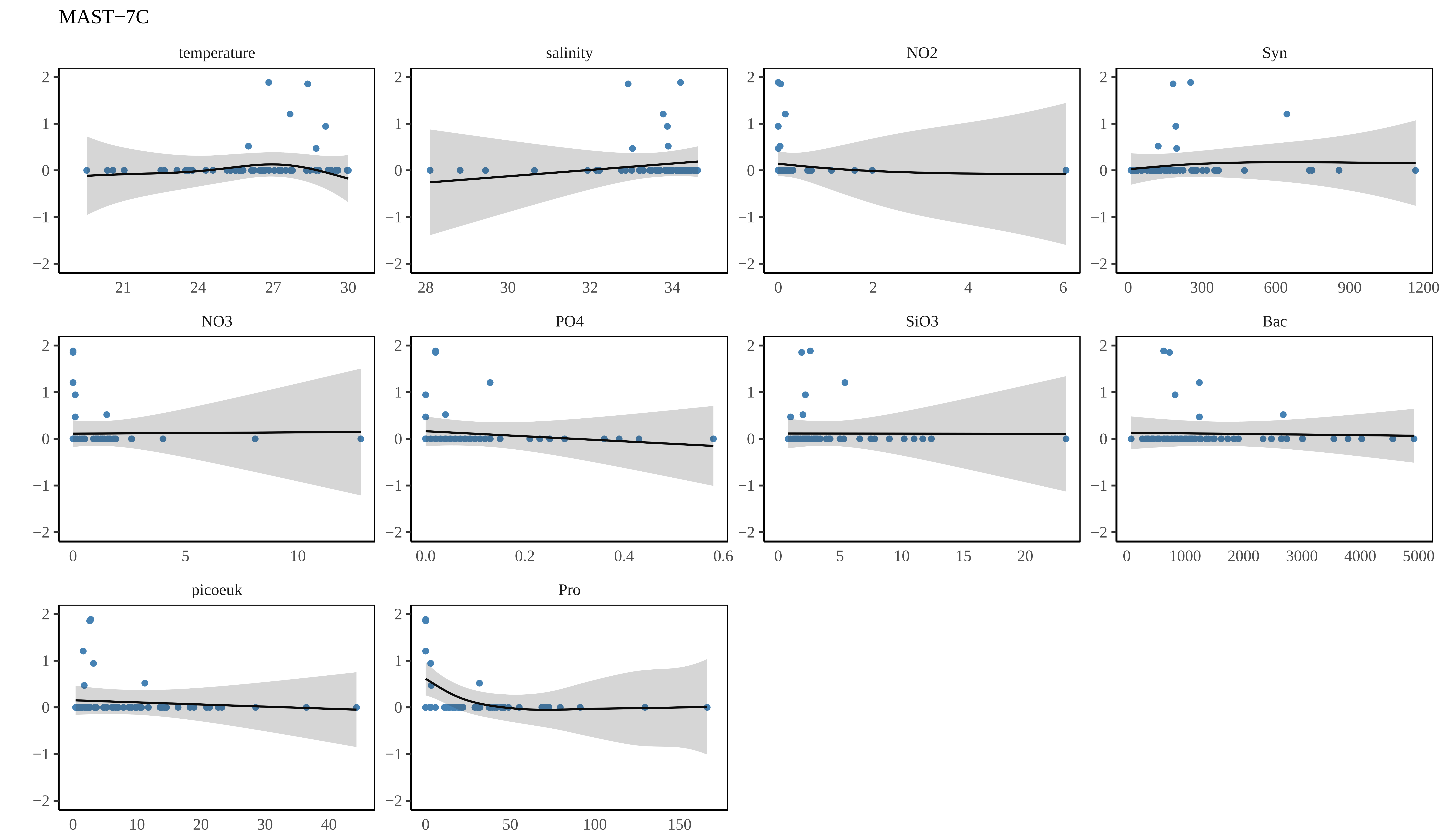
MAST−7C
temperature
salinity
NO2
Syn
NO3
PO4
SiO3
Bac
picoeuk
Pro

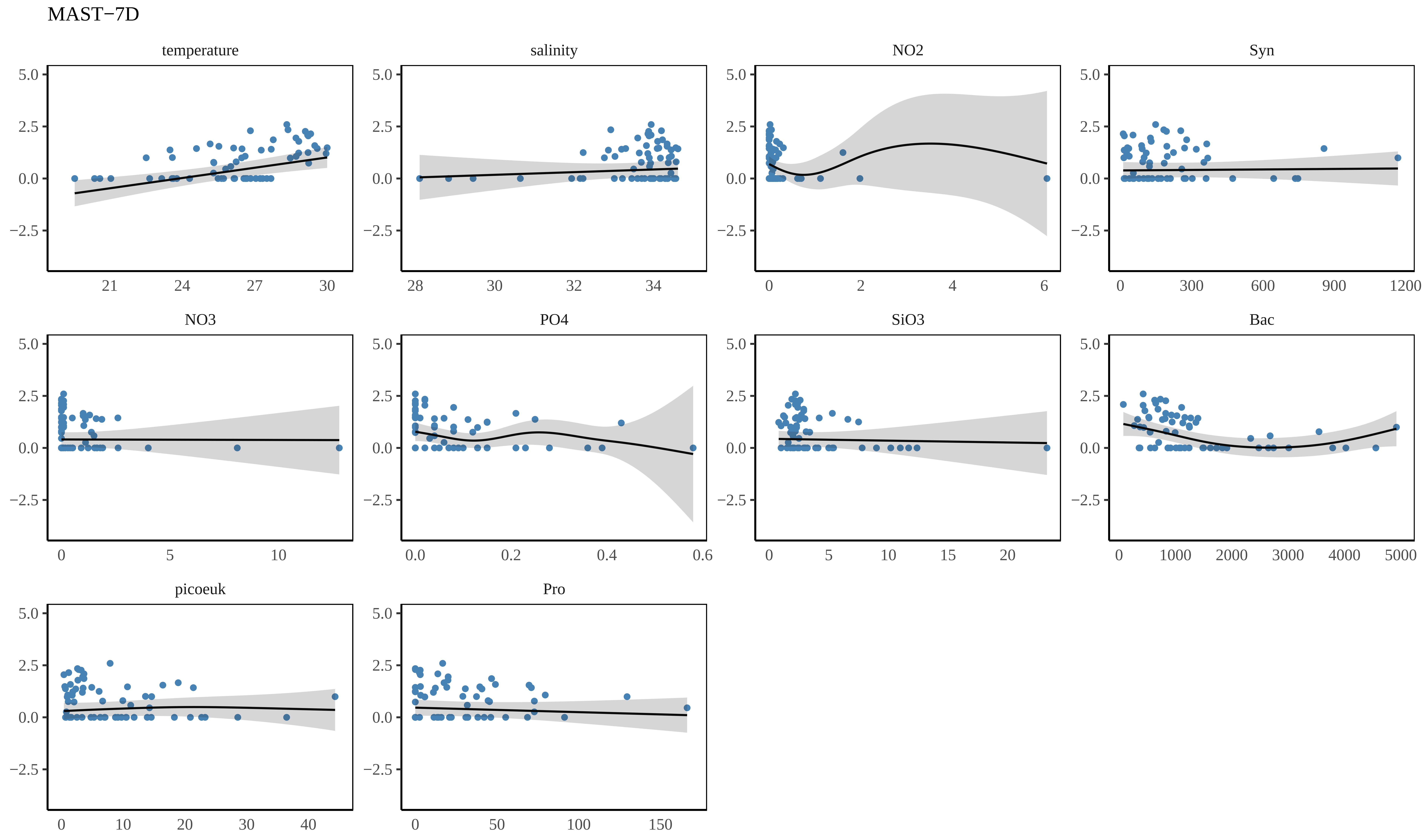
MAST−7D
temperature
salinity
NO2
Syn
NO3
PO4
SiO3
Bac
picoeuk
Pro
5.0
2.5
0.0
−2.5
21
24
27
30
28
30
32
34
0
2
4
6
0
300
600
900
1200
0
5
10
0.0
0.2
0.4
0.6
0
5
10
15
20
0
1000
2000
3000
4000
5000
0
10
20
30
40
0
50
100
150

MAST−7E

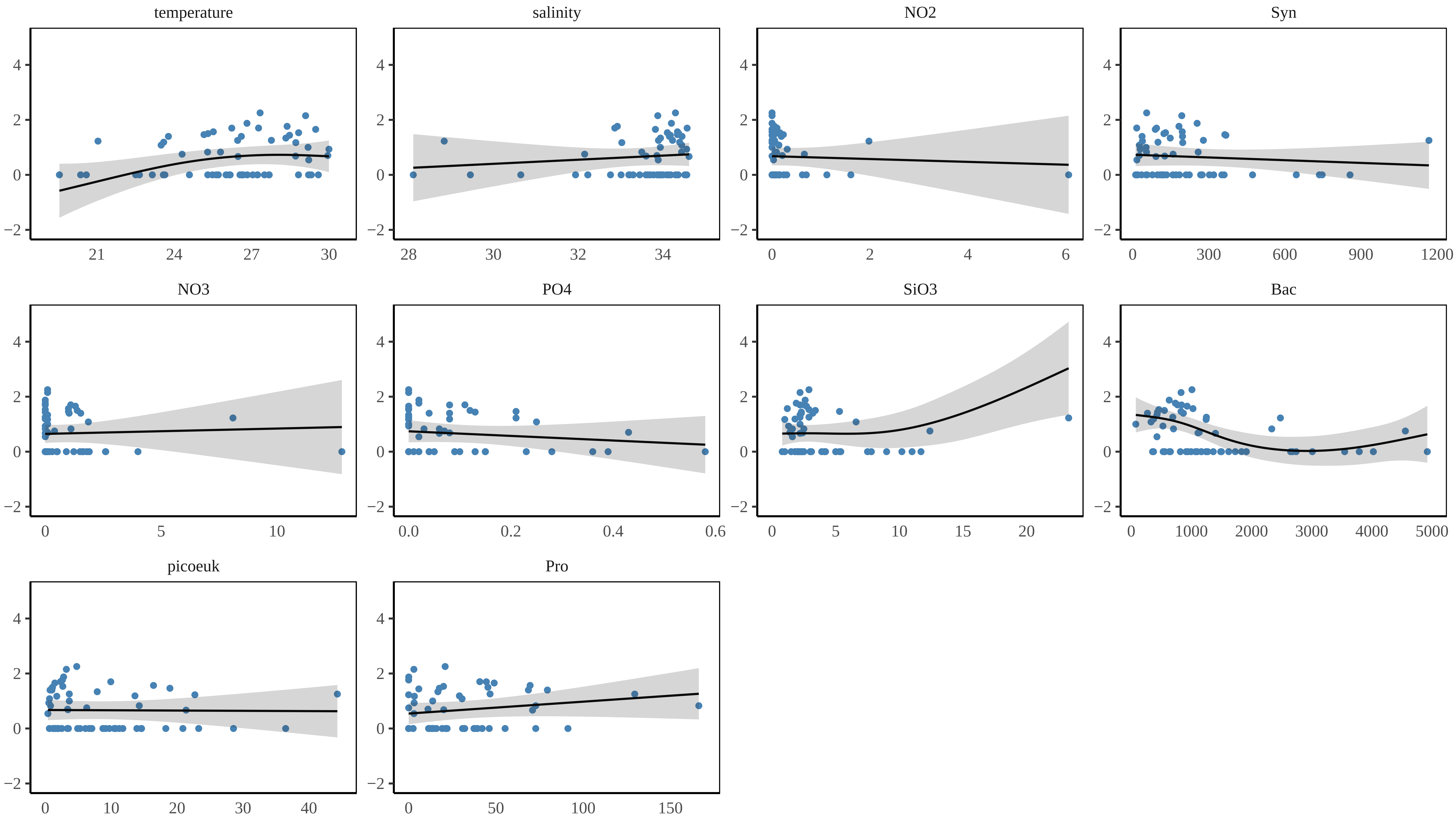
temperature
salinity
NO2
Syn
NO3
PO4
SiO3
Bac
picoeuk
Pro
4
2
0
−2
21
24
27
30
28
30
32
34
0
2
4
6
0
300
600
900
1200
0
5
10
0.0
0.2
0.4
0.6
0
5
10
15
20
0
1000
2000
3000
4000
5000
0
10
20
30
40
0
50
100
150

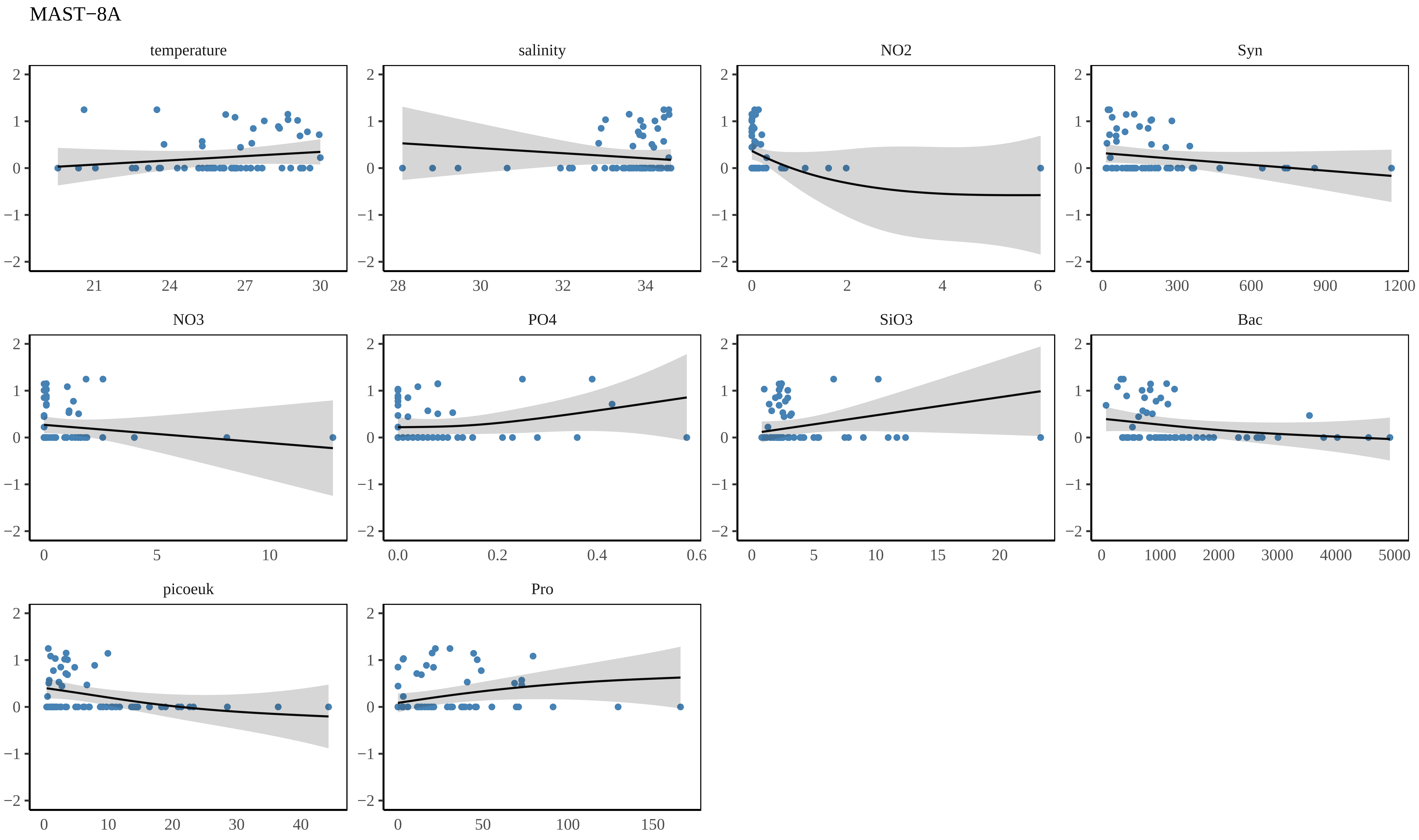
MAST−8A
temperature
salinity
NO2
Syn
NO3
PO4
SiO3
Bac
picoeuk
Pro

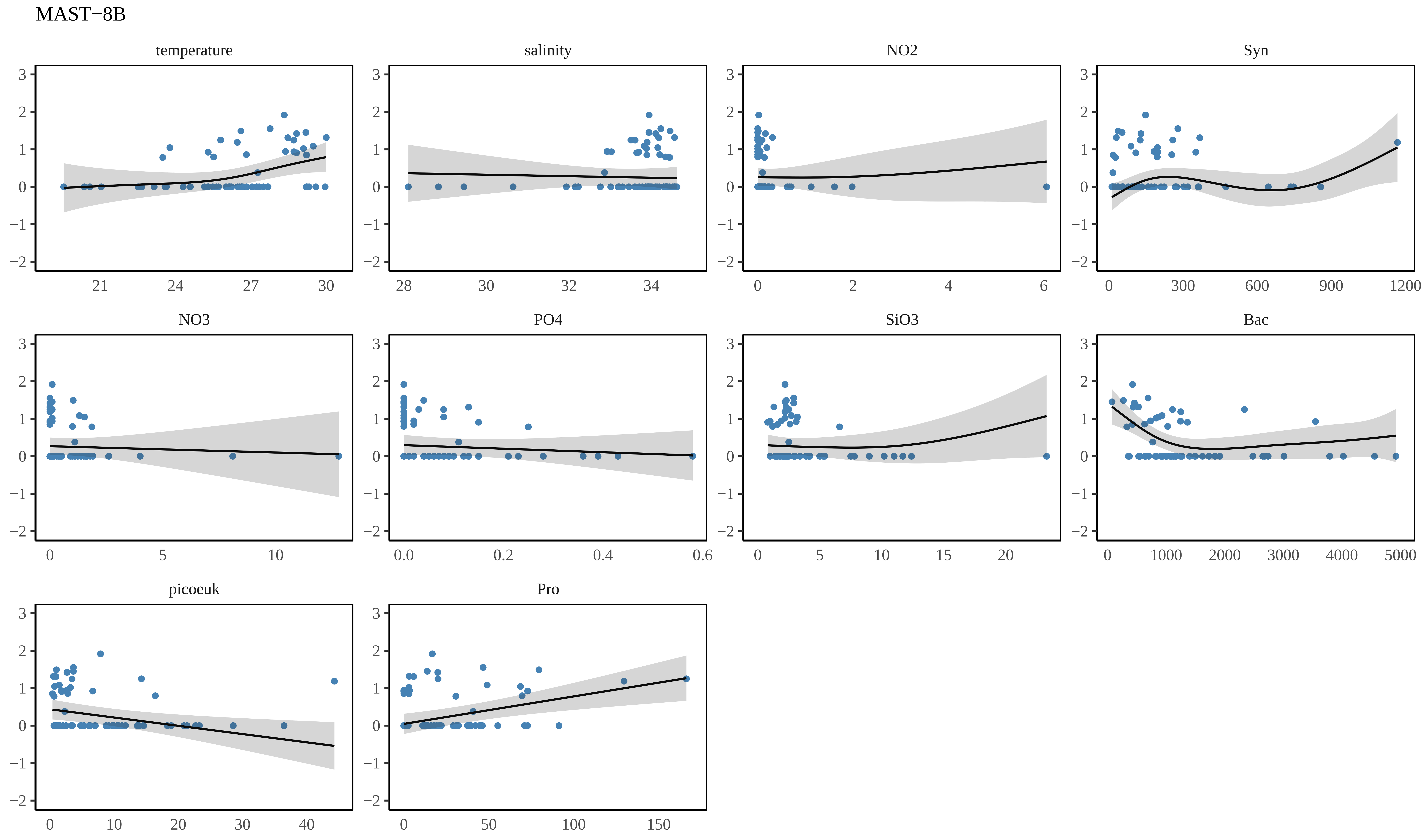
MAST−8B
temperature
salinity
NO2
Syn
NO3
PO4
SiO3
Bac
picoeuk
Pro

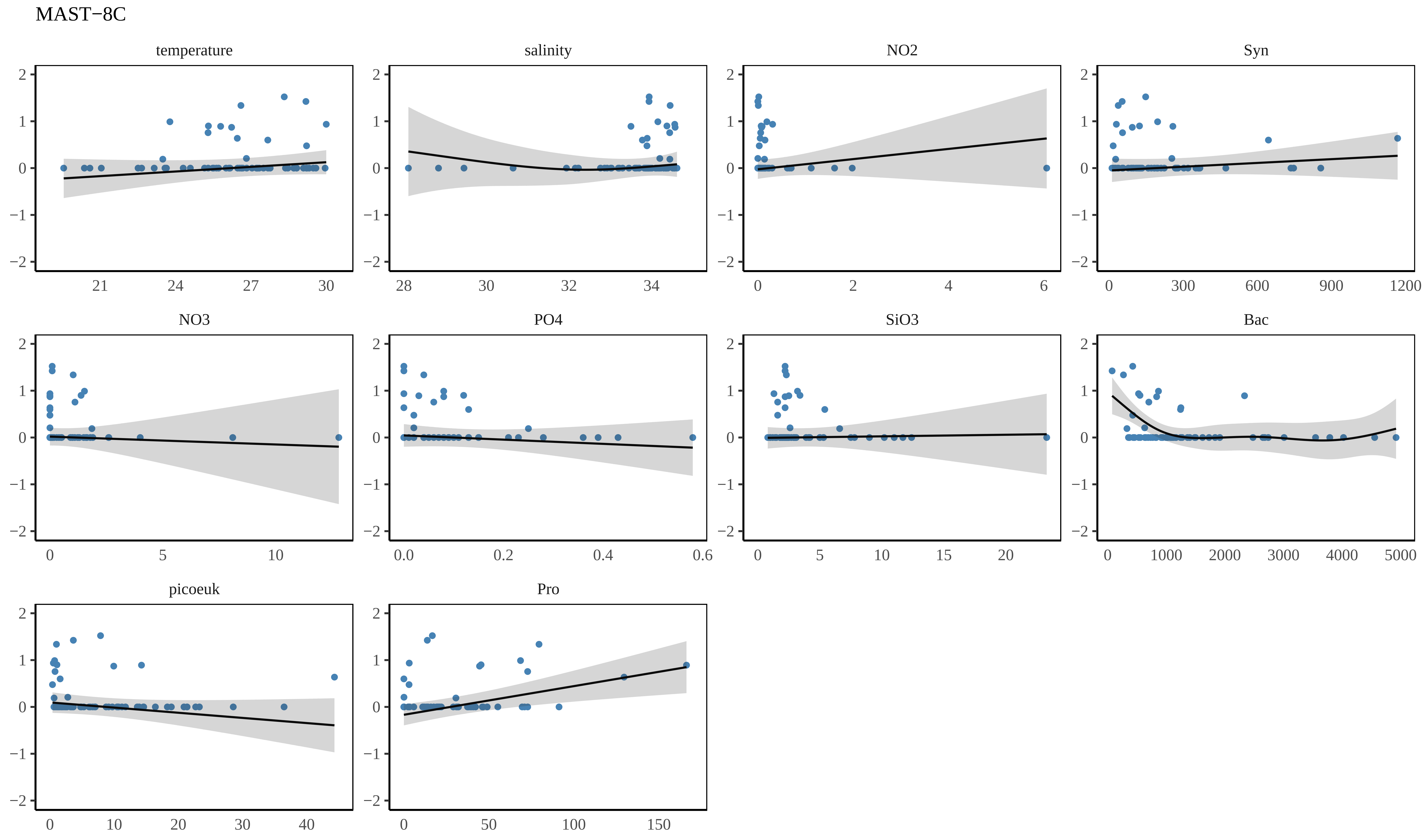
MAST−8C
temperature
salinity
NO2
Syn
NO3
PO4
SiO3
Bac
picoeuk
Pro

MAST−8E

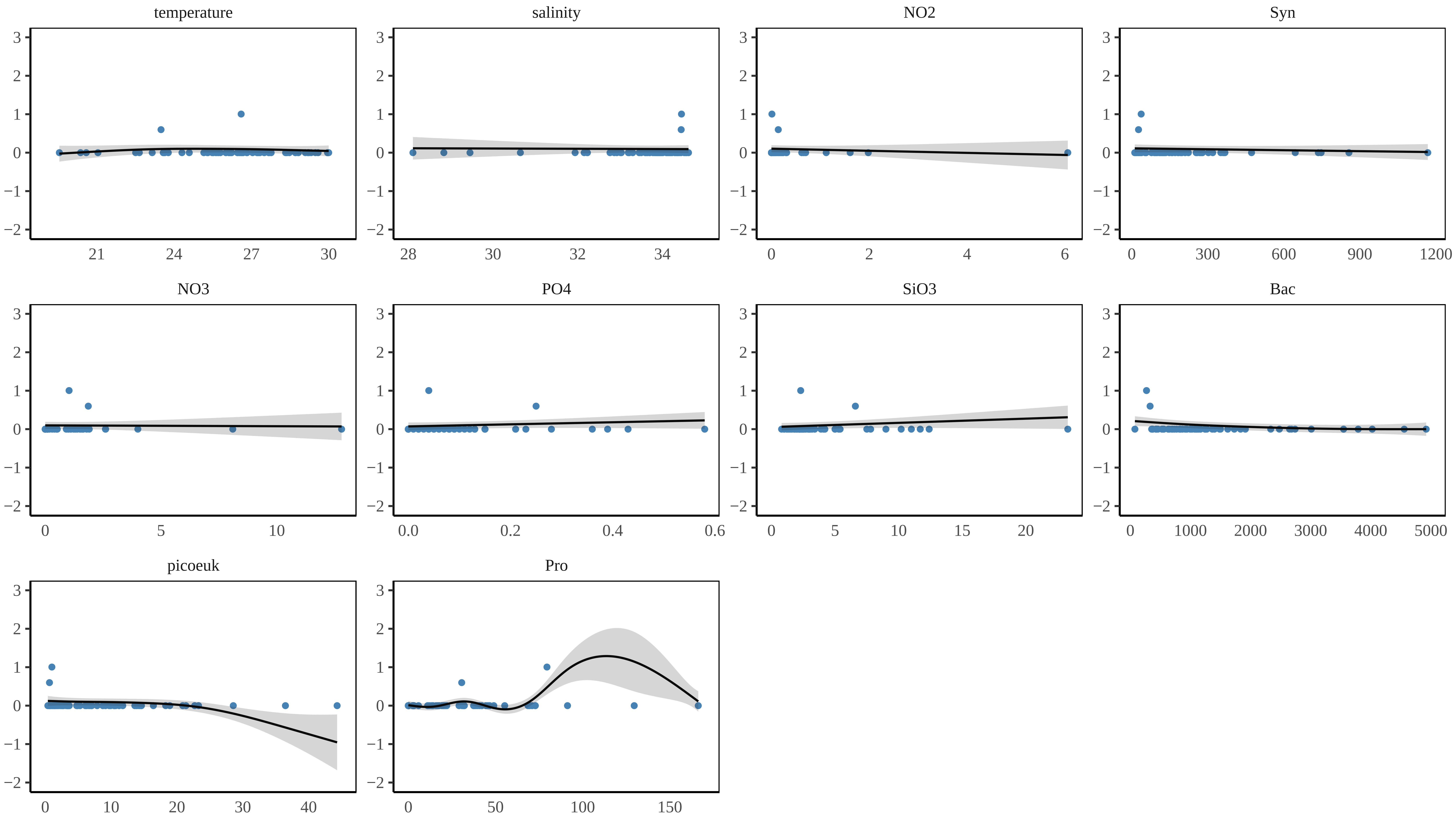

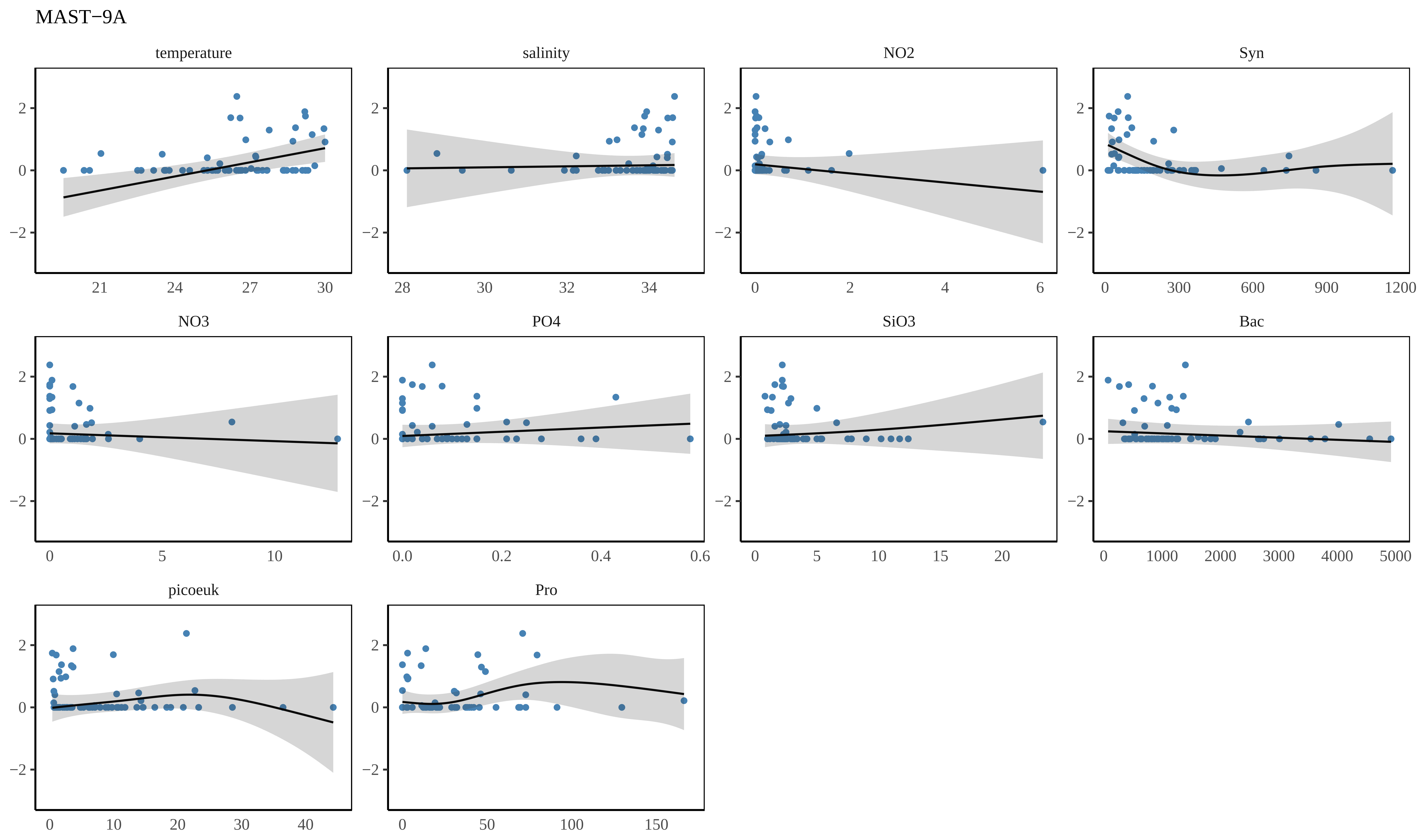
MAST−9A
temperature
salinity
NO2
Syn
NO3
PO4
SiO3
Bac
picoeuk
Pro
2
0
−2
21
24
27
30
28
32
34
4
6
300
600
900
1200
5
10
0.0
0.2
0.4
0.6
15
20
1000
2000
3000
4000
5000
40
50
100
150

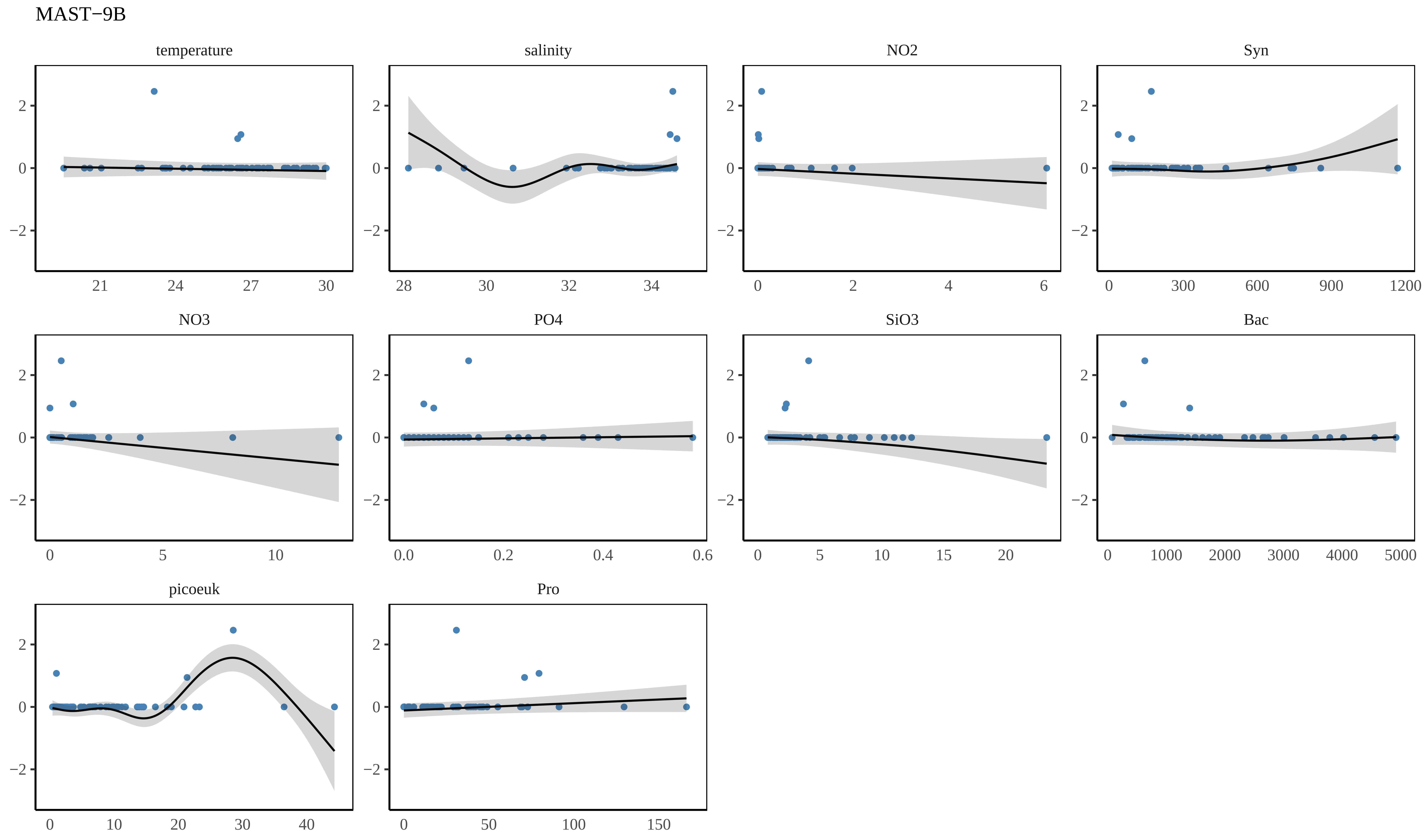
MAST−9B
temperature
salinity
NO2
Syn
NO3
PO4
SiO3
Bac
picoeuk
Pro

MAST−9C

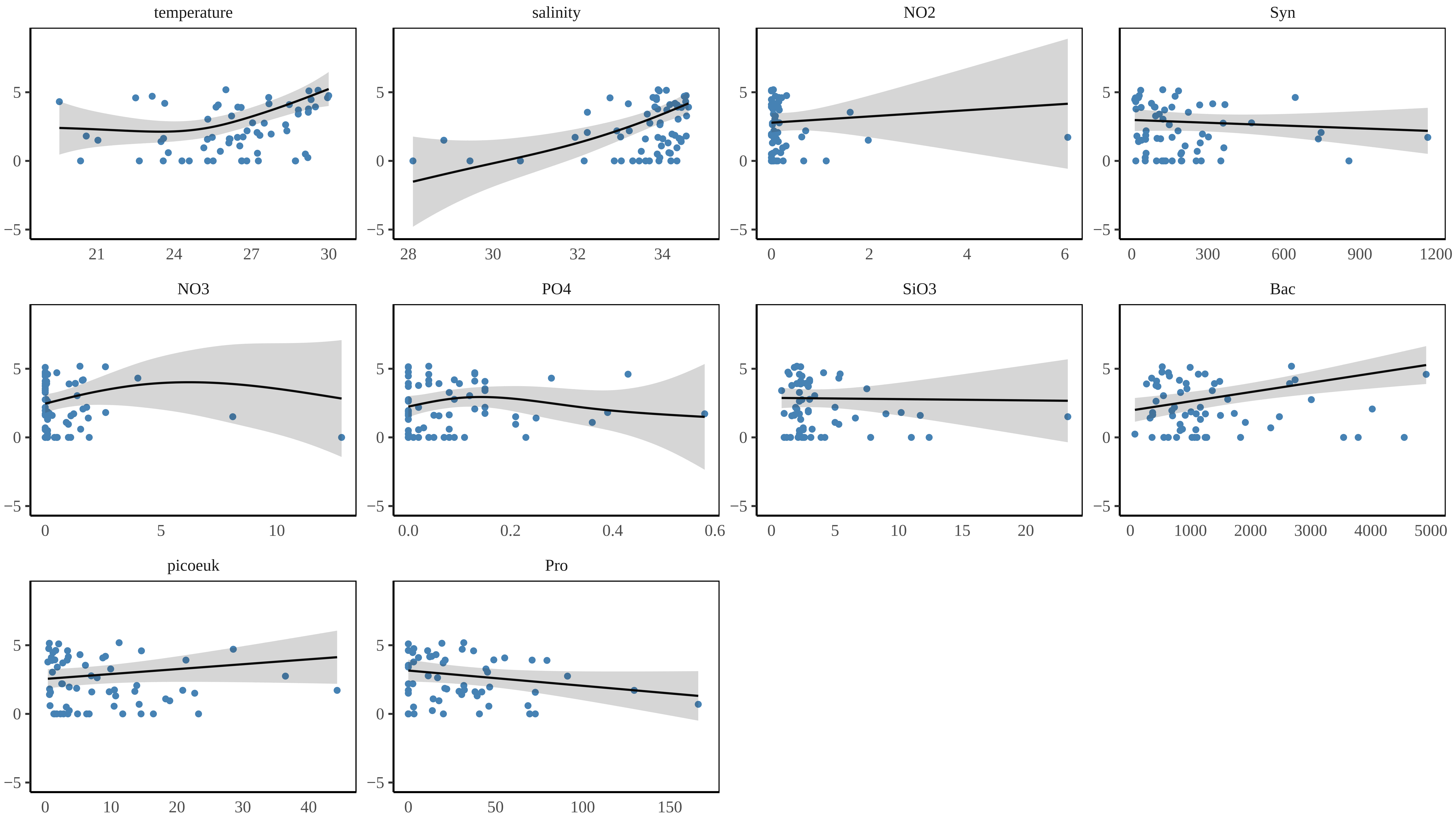

temperature
salinity
NO2
Syn
NO3
PO4
SiO3
Bac
picoeuk
Pro
5
0
−5
21
24
27
30
28
32
34
0
2
4
6
300
600
900
1200
10
0.0
0.2
0.4
0.6
15
20
1000
2000
3000
4000
5000
40
50
100
150

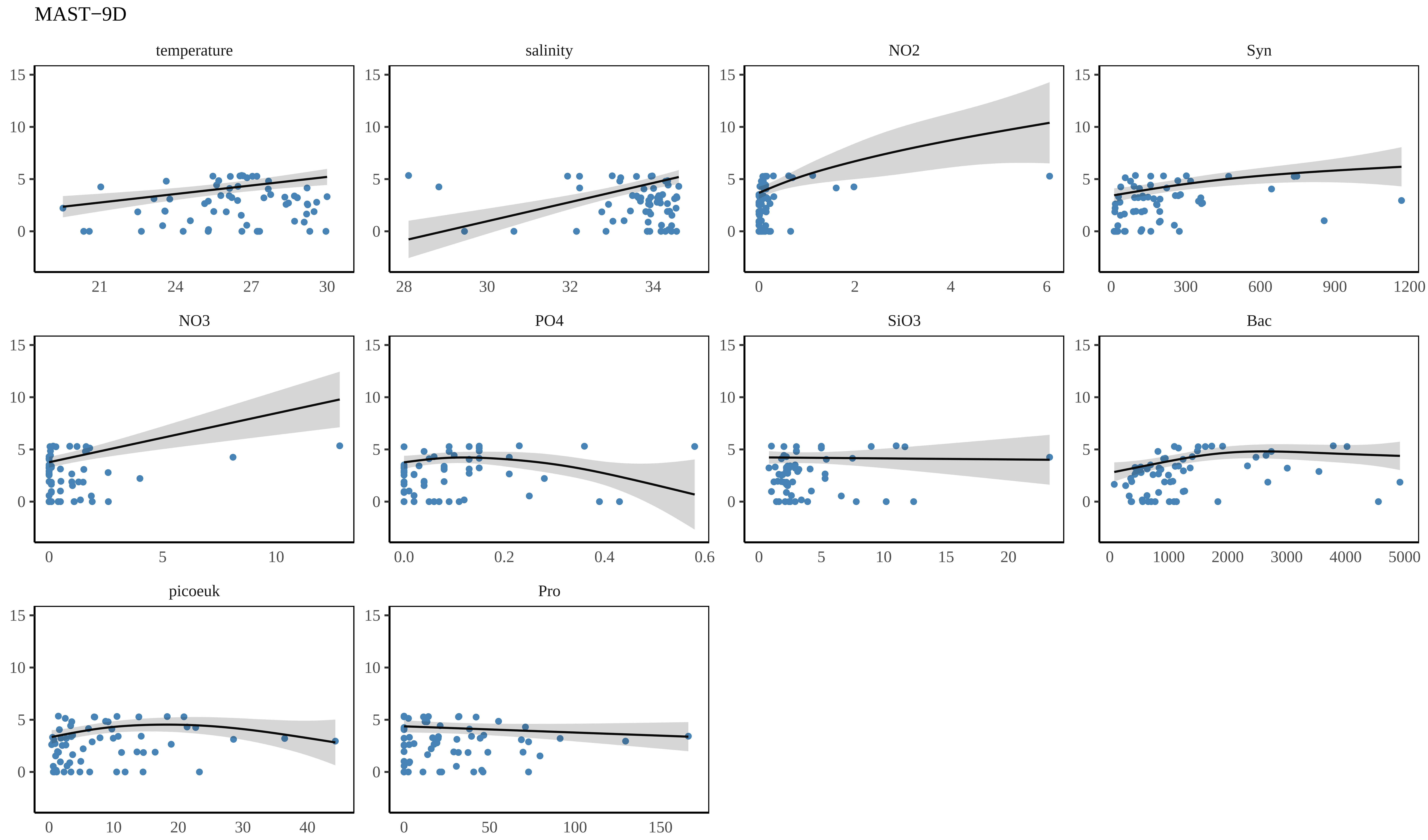
MAST−9D
temperature
salinity
NO2
Syn
NO3
PO4
SiO3
Bac
picoeuk
Pro
15
10
5
0
21
24
27
30
28
30
32
34
0
2
4
6
0
300
600
900
1200
0
5
10
0.0
0.2
0.4
0.6
0
5
10
15
20
0
1000
2000
3000
4000
5000
0
10
20
30
40
0
50
100
150

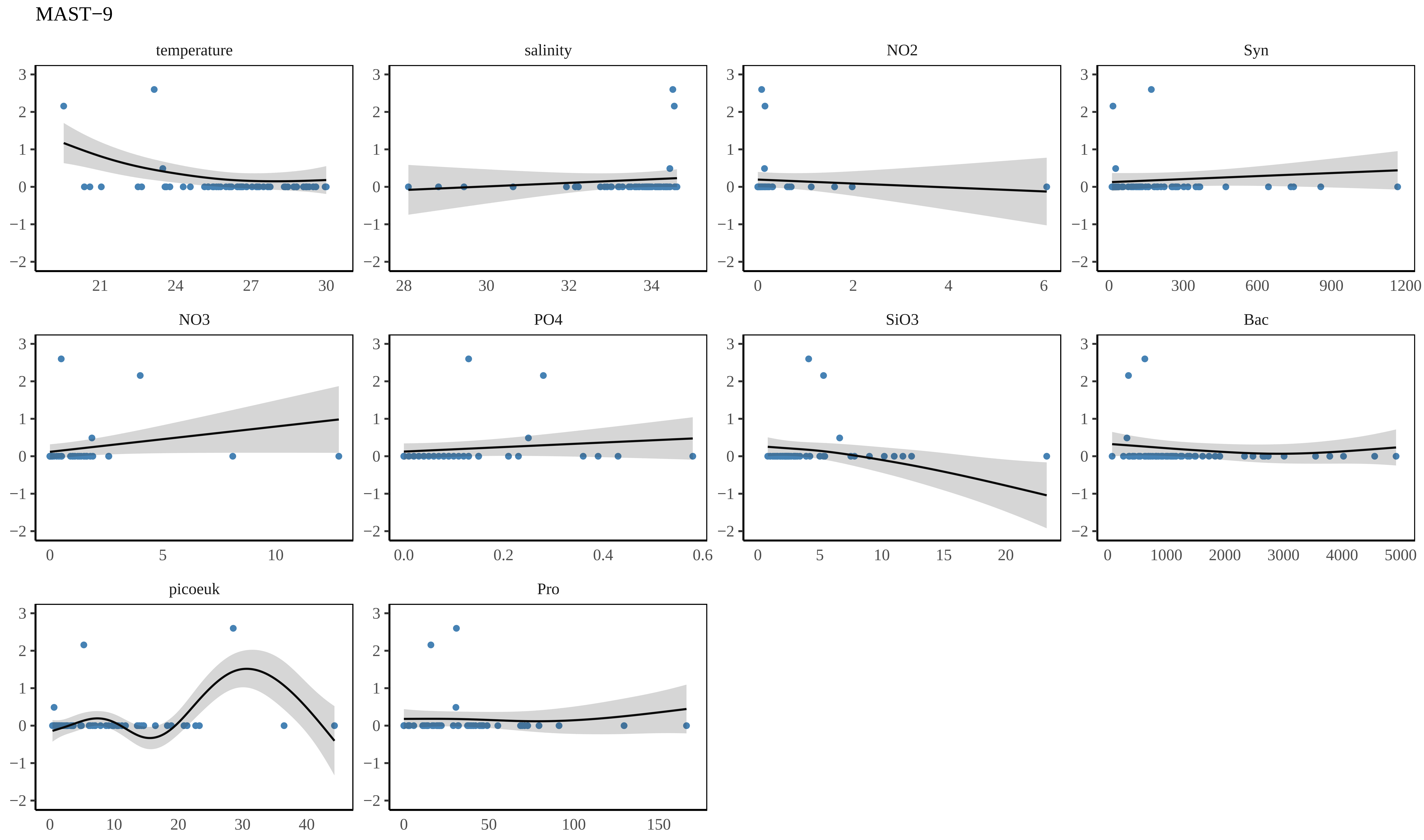
MAST−9
temperature
salinity
NO2
Syn
NO3
PO4
SiO3
Bac
picoeuk
Pro

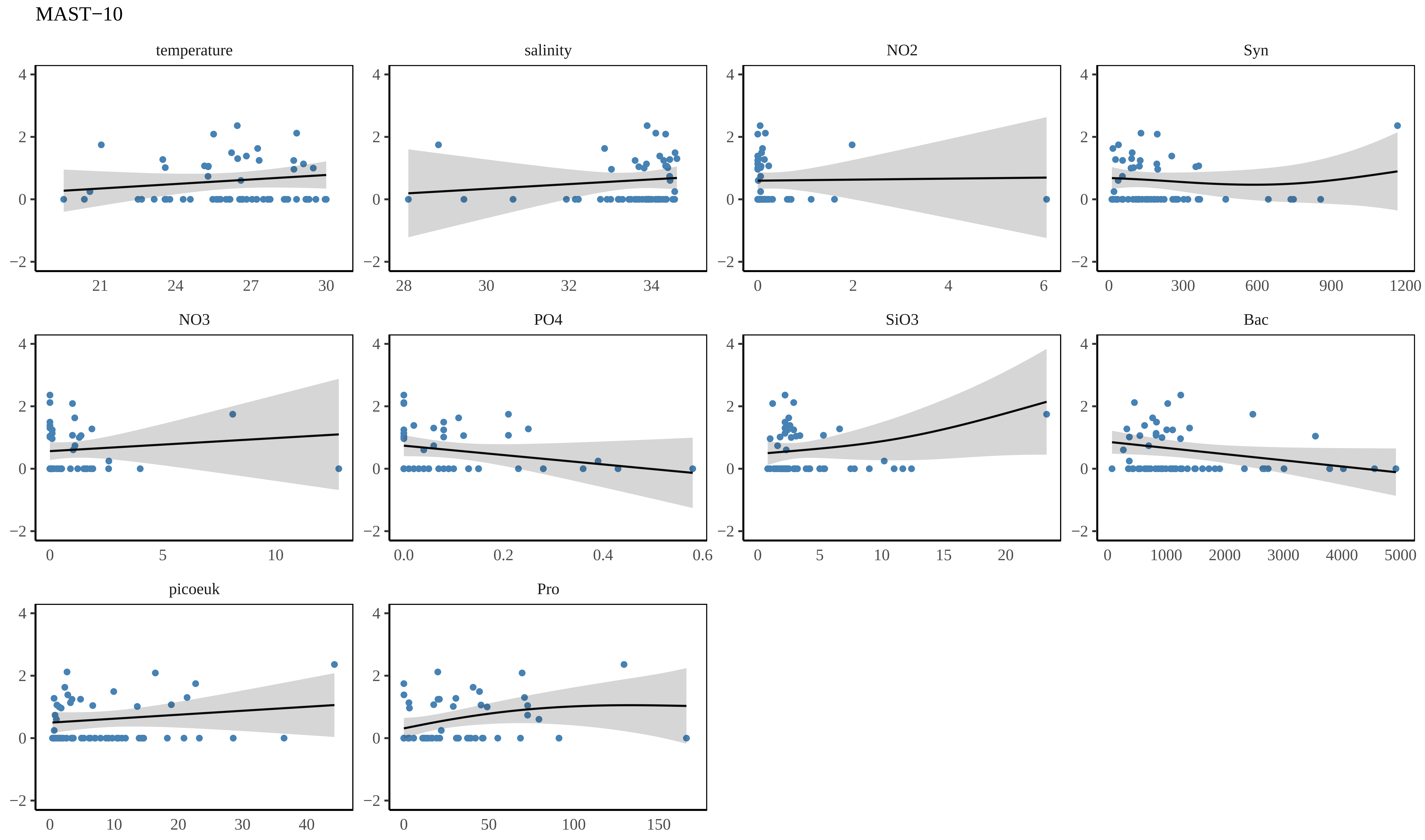
MAST−10
temperature
salinity
NO2
Syn
NO3
PO4
SiO3
Bac
picoeuk
Pro
4
2
0
−2
21
24
27
30
28
30
32
34
0
2
4
6
0
300
600
900
1200
5
10
0.0
0.2
0.4
0.6
15
20
1000
2000
3000
4000
5000
40
50
100
150

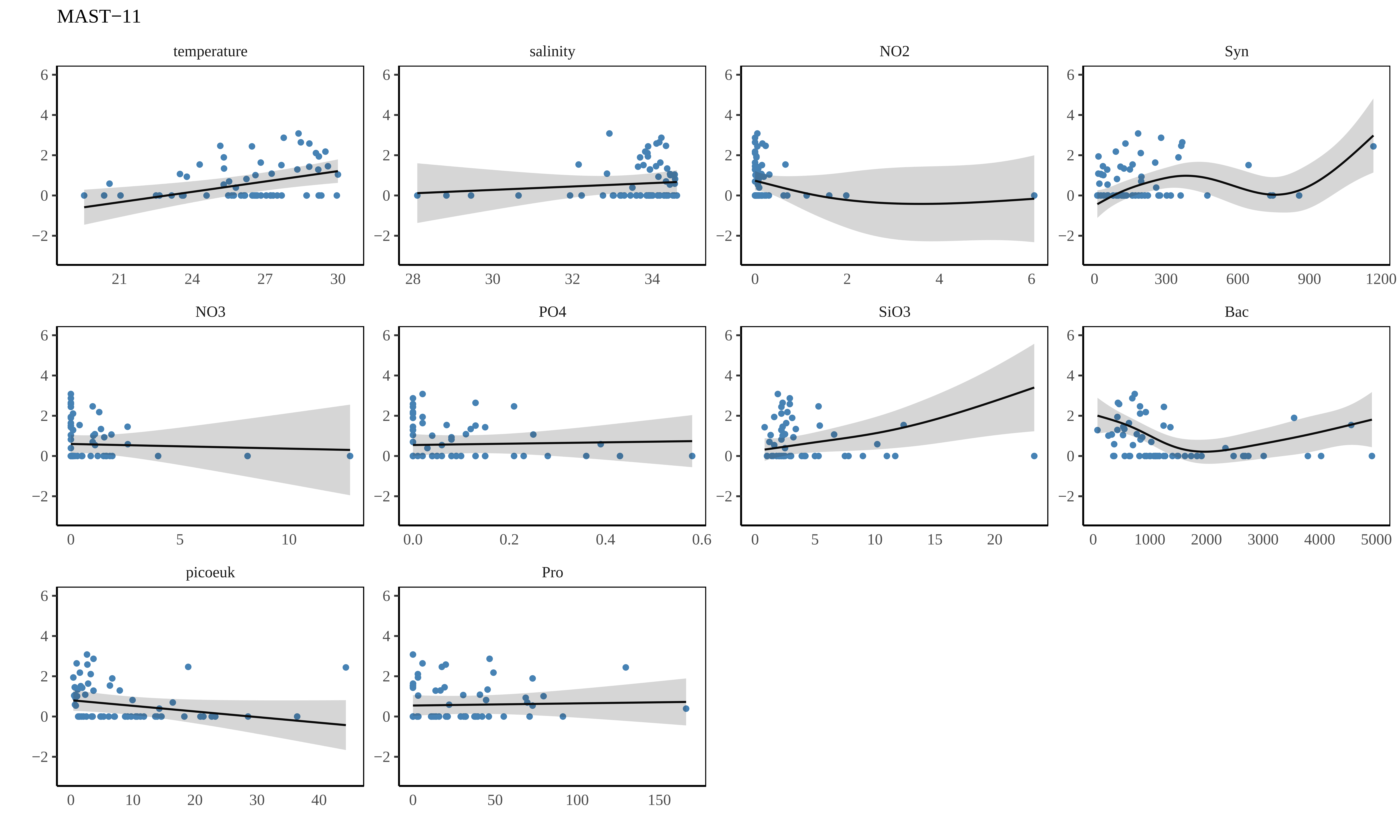

MAST−11
temperature
salinity
NO2
Syn
NO3
PO4
SiO3
Bac
picoeuk
Pro

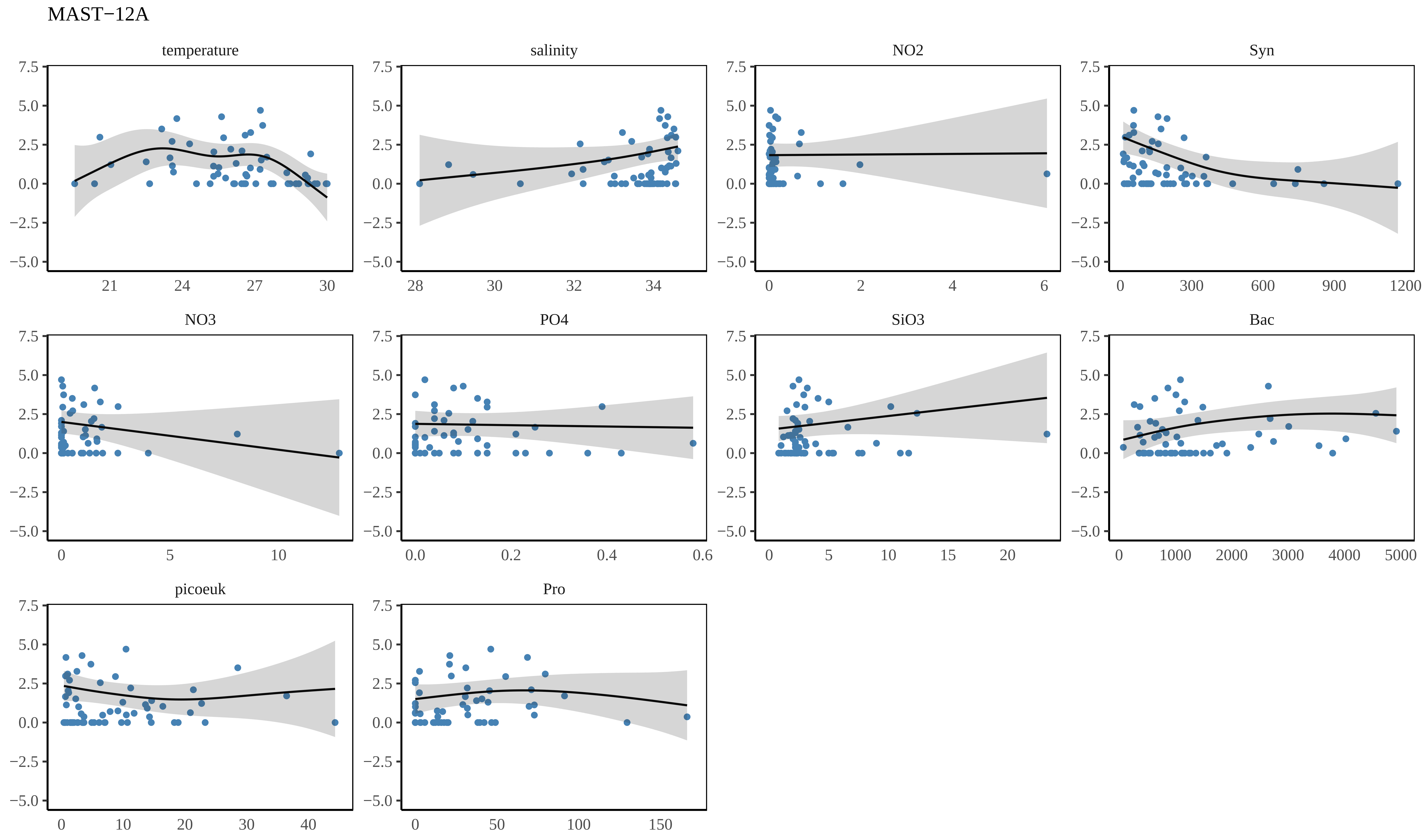
MAST−12A
temperature
salinity
NO2
Syn
NO3
PO4
SiO3
Bac
picoeuk
Pro

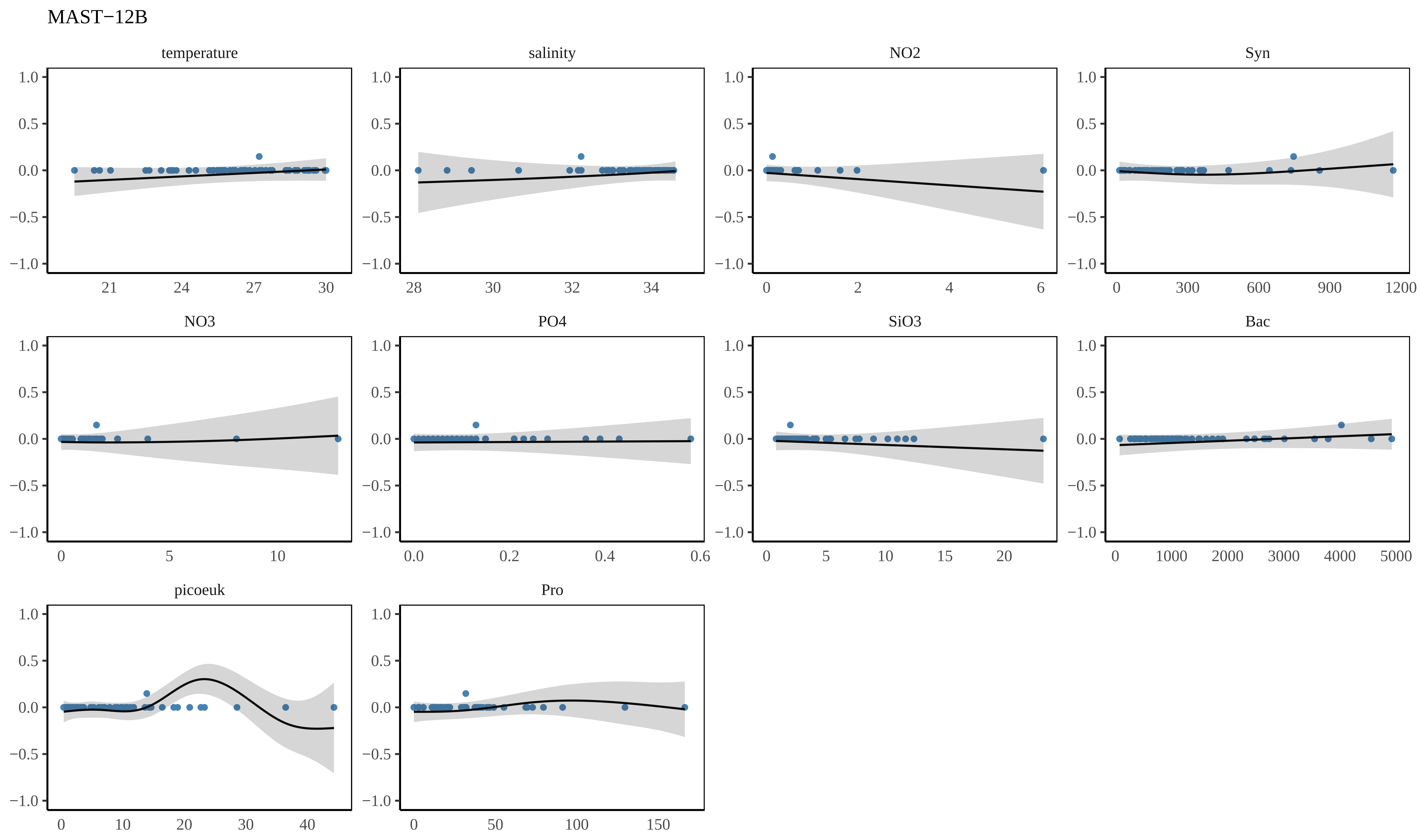
MAST−12B
temperature
salinity
NO2
Syn
NO3
PO4
SiO3
Bac
picoeuk
Pro

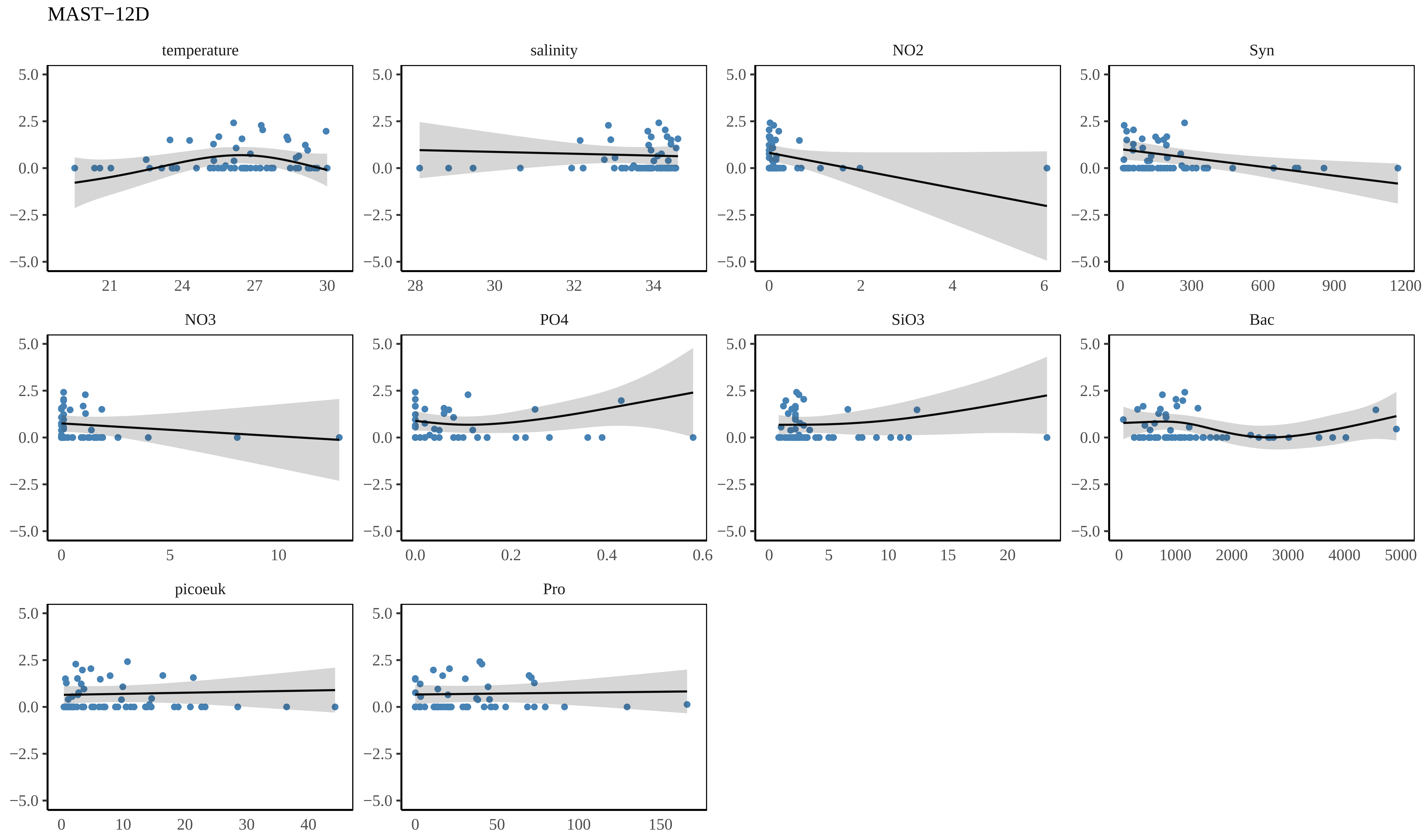
MAST−12D
temperature
salinity
NO2
Syn
NO3
PO4
SiO3
Bac
picoeuk
Pro
5.0
2.5
0.0
−2.5
−5.0
21
24
27
30
28
30
32
34
0
2
4
6
0
300
600
900
1200
0
5
10
0.0
0.2
0.4
0.6
0
5
10
15
20
0
1000
2000
3000
4000
5000
0
10
20
30
40
0
50
100
150

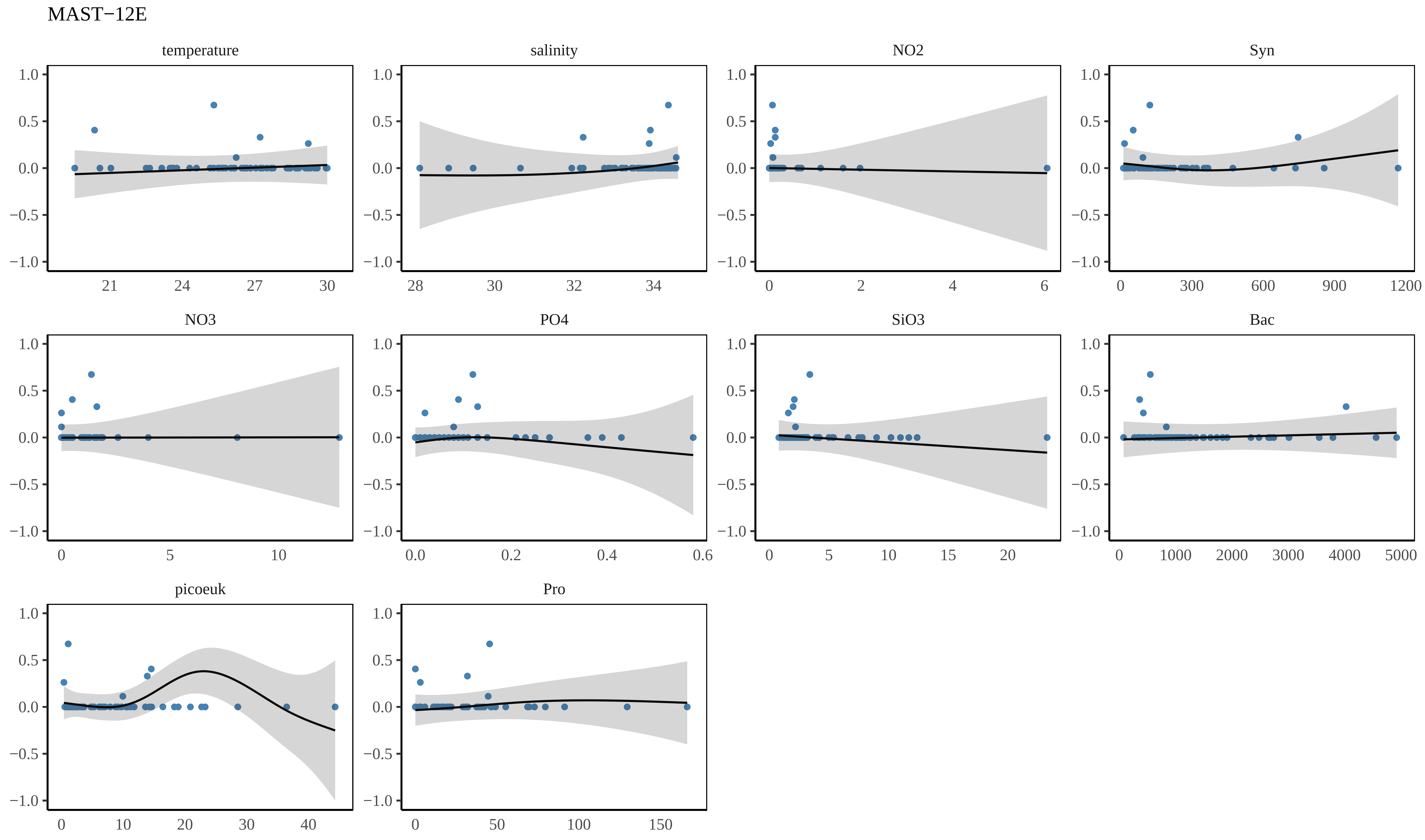
MAST−12E
temperature
salinity
NO2
Syn
NO3
PO4
SiO3
Bac
picoeuk
Pro

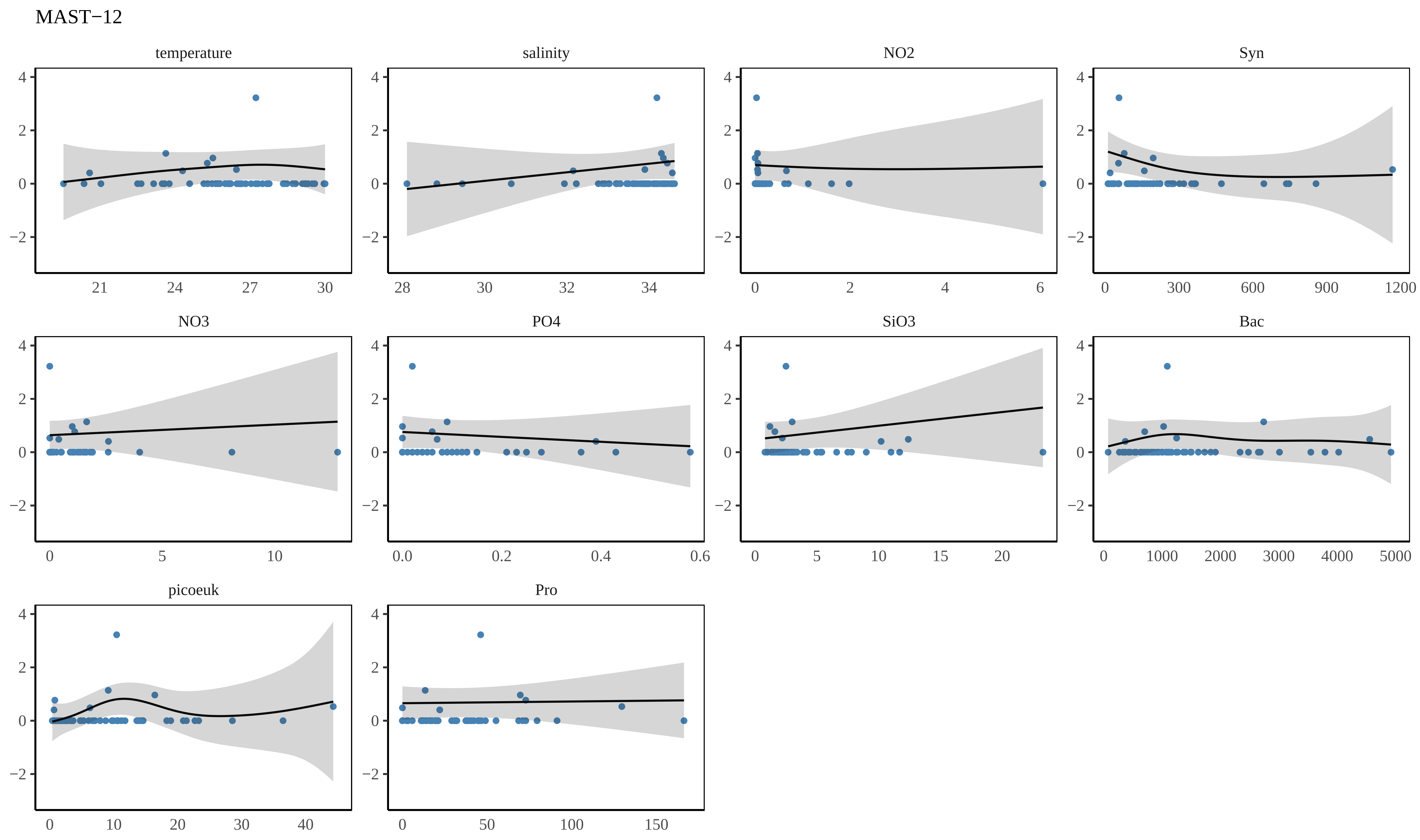

MAST−12
temperature
salinity
NO2
Syn
NO3
PO4
SiO3
Bac
picoeuk
Pro

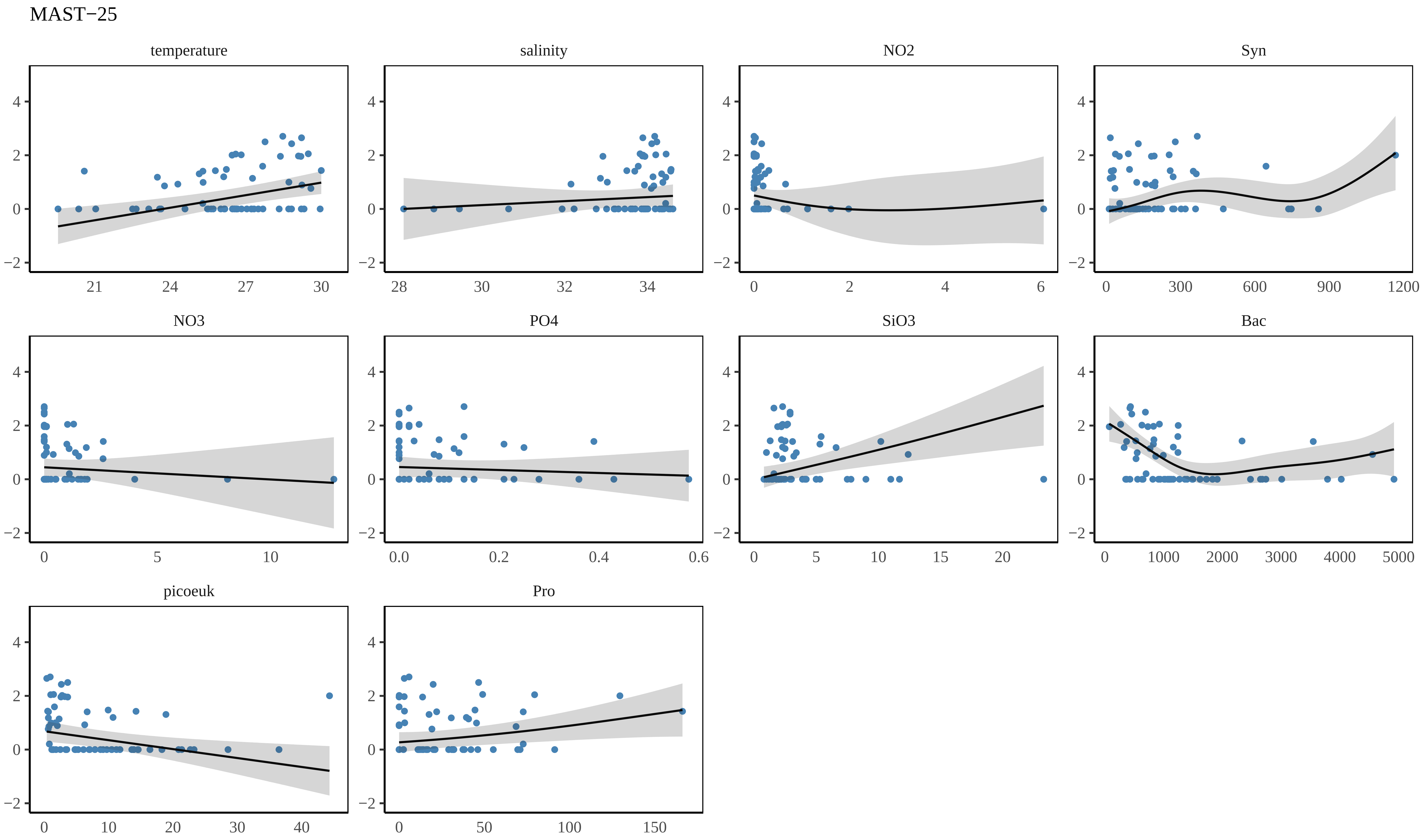
MAST−25
temperature
salinity
NO2
Syn
NO3
PO4
SiO3
Bac
picoeuk
Pro
