# Supplementary figures and images for: How Communities of Marine Stramenopiles Varied with Environmental and Biological Variables in the Subtropical Northwestern Pacific Ocean
Source: Microb Ecol. 2021 Jul 16;83(4):916–28. doi: 10.1007/s00248-021-01788-7 (PMC9015972; doi:10.1007/s00248-021-01788-7)

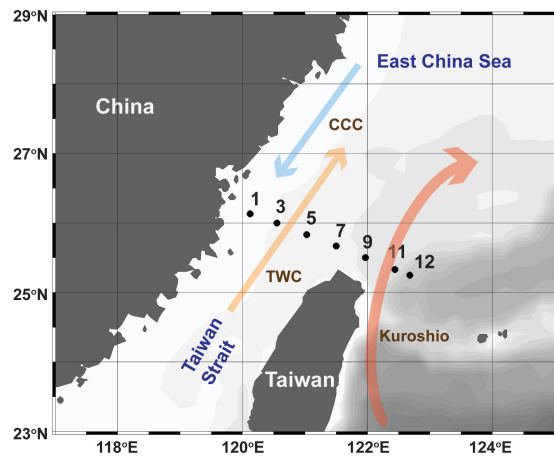

74 x 60 mm

Fig. S1

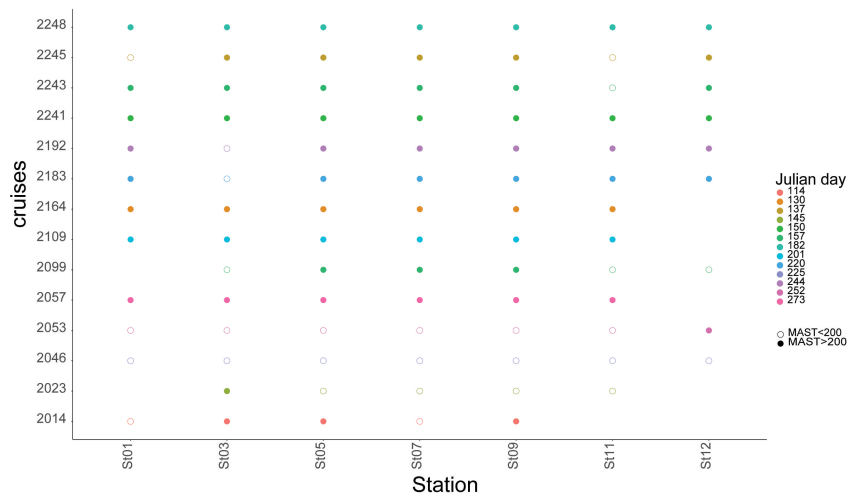

111 x 65 mm

Fig. S2

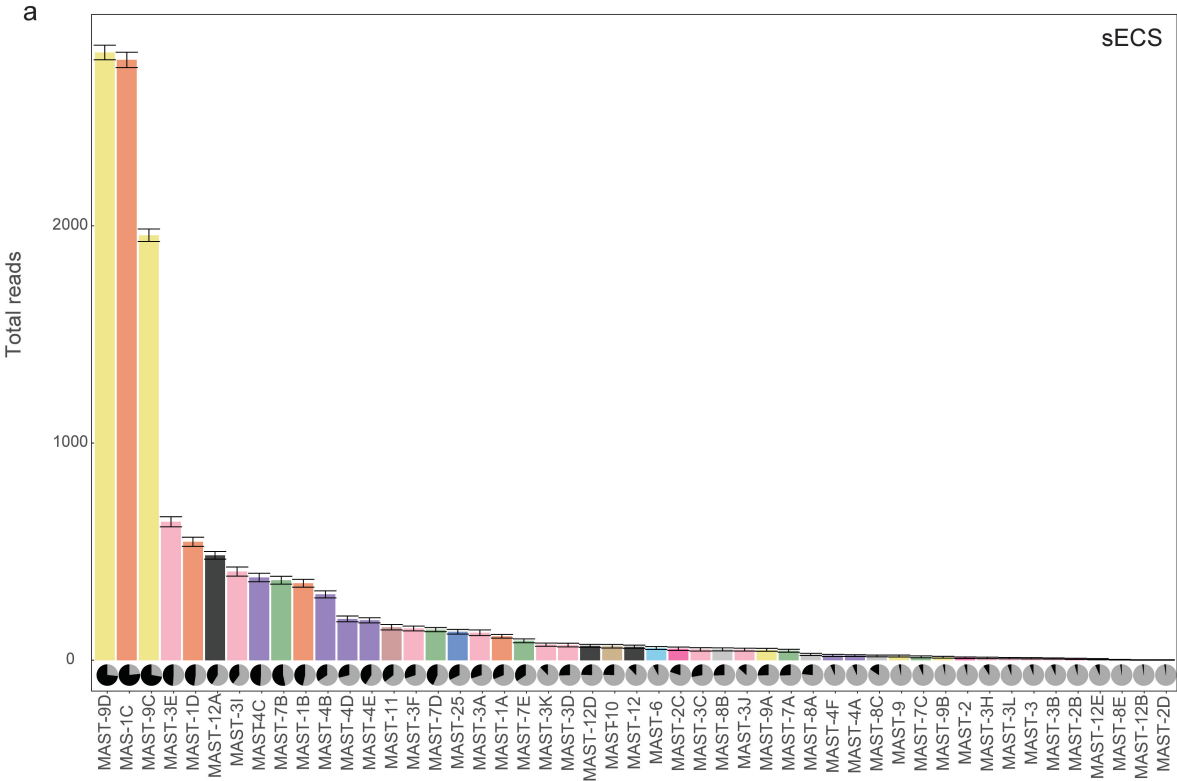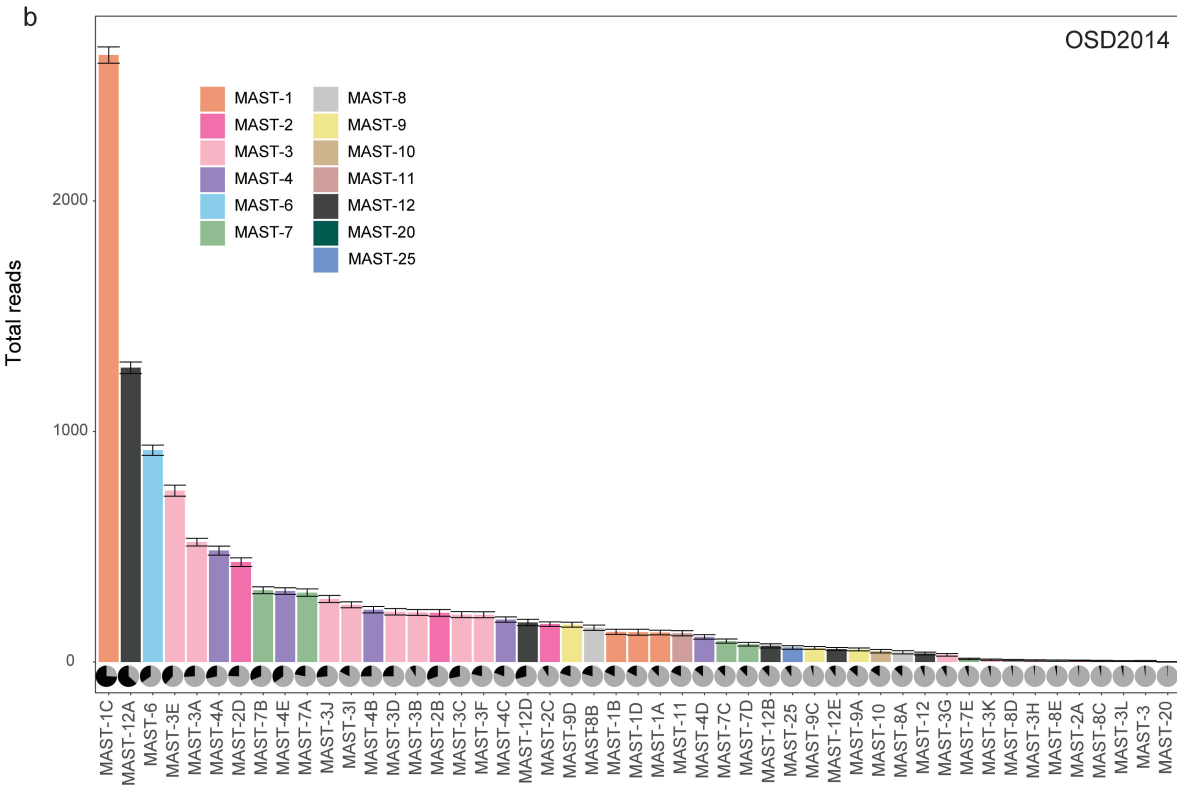

156 x 213 mm

Fig. S3

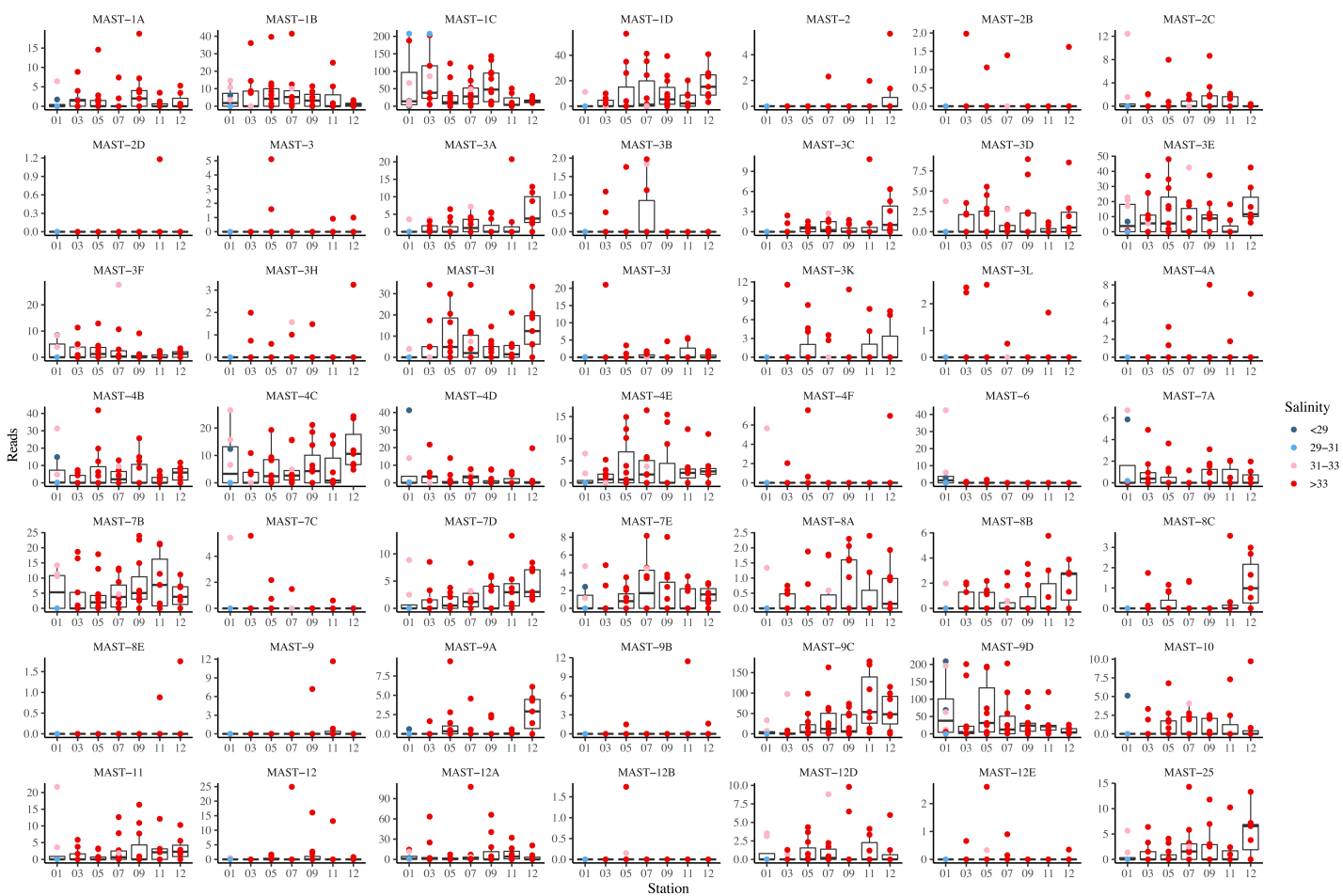

190 x 127 mm

Fig. S4

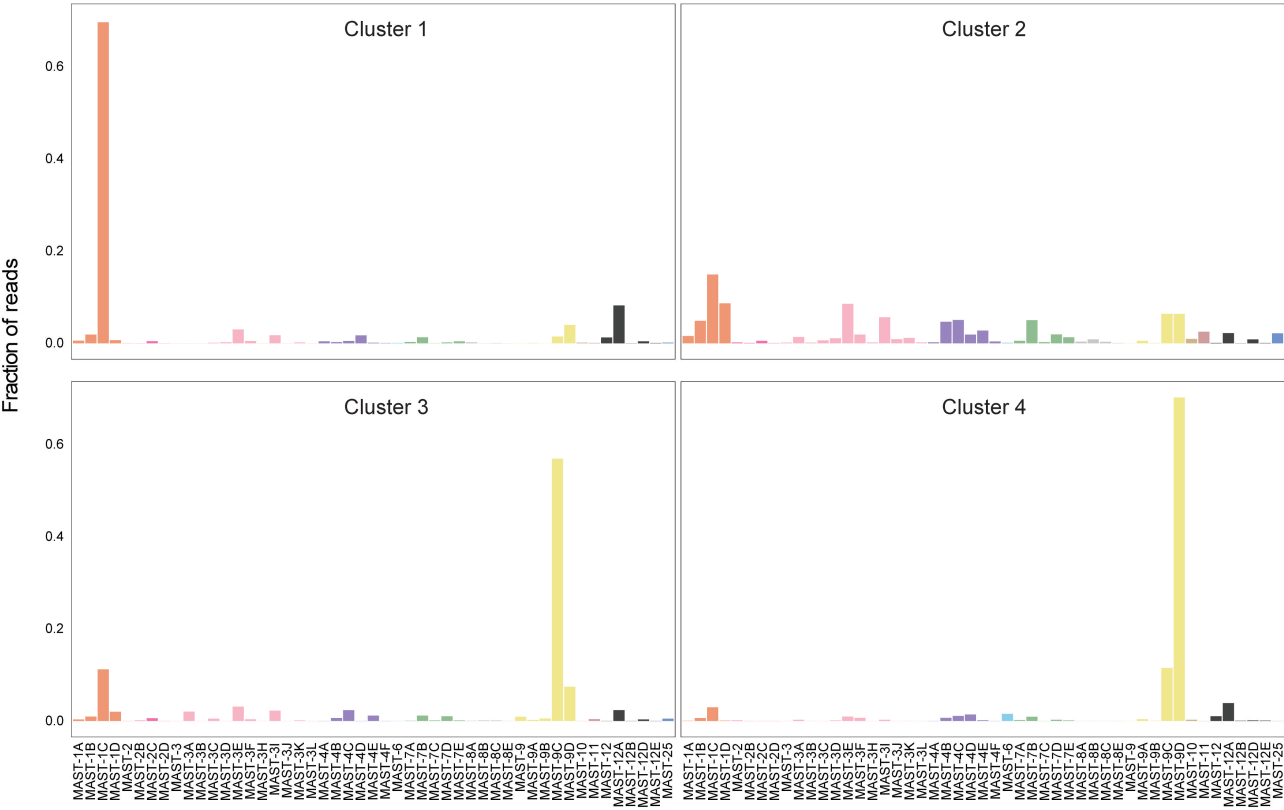

170 x 106 mm

Fig. S5

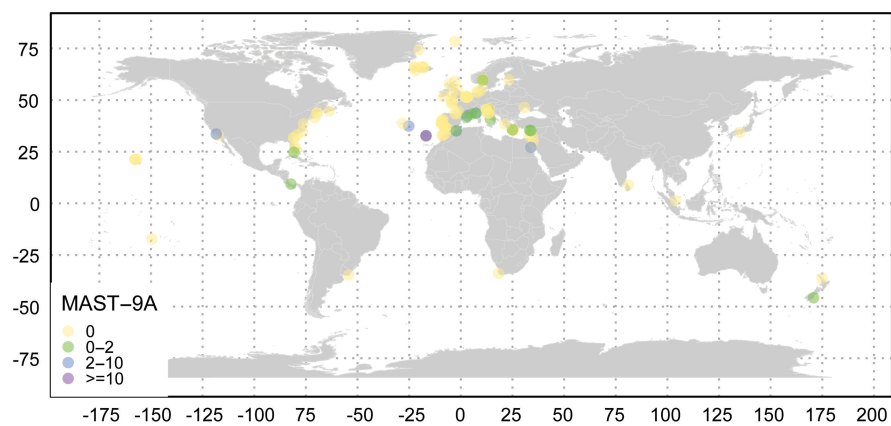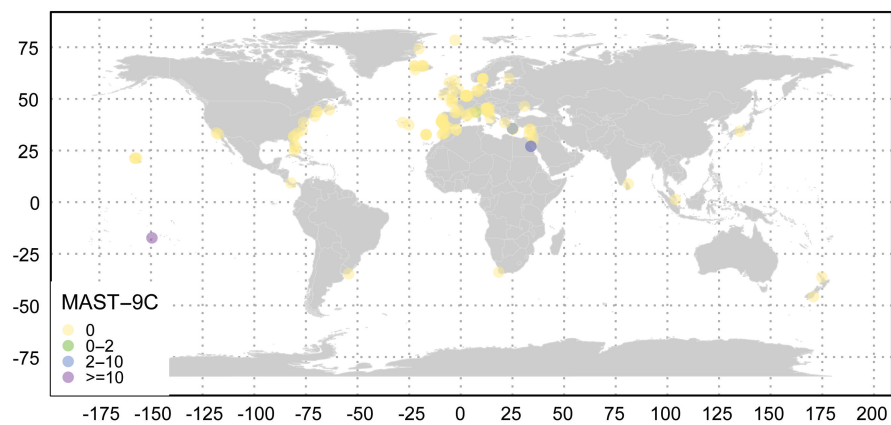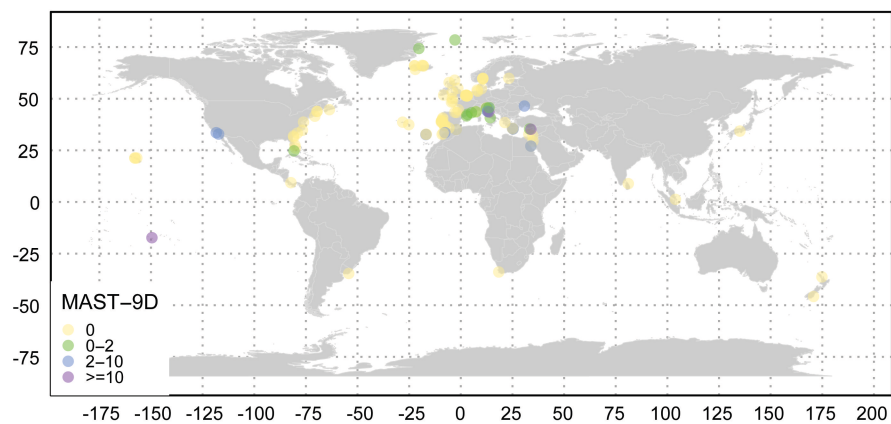

120 x 240 mm

Fig. S6

Supplement: Supplementary file 8 — Supplementary file8 (PDF 17144 KB) [file 248_2021_1788_MOESM8_ESM.pdf]
